# The association between epilepsy and COVID-19: analysis based on Mendelian randomization and FUMA

**DOI:** 10.3389/fnins.2023.1235822

**Published:** 2023-09-15

**Authors:** Mingyao You, Ping Yuan, Liangqian Li, Baoduo Li, Zijun Peng, Hongbei Xu

**Affiliations:** Department of Neurology, The Affiliated Hospital of Guizhou Medical University, Guizhou, China

**Keywords:** epilepsy, COVID-19, Mendelian randomization, causal relationship, FUMA

## Abstract

**Objective:**

A multitude of observational studies have underscored a substantial comorbidity between COVID-19 and epilepsy. This study was aimed at establishing a conclusive causal link between these two conditions.

**Methods:**

We employed Mendelian randomization (MR) to evaluate the causal link between COVID-19 and epilepsy, as well as its focal and generalized subtypes. The GWAS for epilepsy and its subtypes database were abstracted from both FinnGen consortium and ILAE. Additionally, we leveraged functional mapping and annotation (FUMA) to integrate information from genome-wide association studies (GWAS) results.

**Results:**

The MR analyses revealed that genetic liability to COVID-19 infection conferred a causal effect on epilepsy [FinnGen: OR: 1.5306; 95% confidence interval (CI): 1.1676–2.0062, *P*_*FDR*_ (false discovery rate) = 0.0076; ILAE: OR: 1.3440; 95% CI: 1.0235–1.7649, *P_*FDR*_* = 0.0429], and generalized epilepsy (FinnGen: OR: 2.1155; 95% CI: 1.1734–3.8139, *P_*FDR*_* = 0.0327; ILAE: OR: 1.1245; 95% CI: 1.0444–1.2108, *P_*FDR*_* = 0.0114). Genetic liability to COVID-19 hospitalization conferred a causal effect on epilepsy (FinnGen: OR: 1.0934; 95% CI: 1.0097–1.1841, *P_*FDR*_* = 0.0422; ILAE: OR: 1.7381; 95% CI: 1.0467–2.8862, *P_*FDR*_* = 0.0451), focal epilepsy (ILAE: OR: 1.7549; 95% CI: 1.1063–2.7838, *P_*FDR*_* = 0.0338), and generalized epilepsy (ILAE: OR: 1.1827; 95% CI: 1.0215–1.3693, *P_*FDR*_* = 0.0406). Genetic liability to COVID-19 severity conferred a causal effect on epilepsy (FinnGen consortium: OR: 1.2454; 95% CI: 1.0850–1.4295, *P_*FDR*_* = 0.0162; ILAE: OR: 1.2724; 95% CI: 1.0347–1.5647, *P_*FDR*_* = 0.0403), focal epilepsy (FinnGen: OR: 1.6818; 95% CI: 1.1478–2.4642, *P_*FDR*_* = 0.0231; ILAE: OR: 1.6598; 95% CI: 1.2572–2.1914, *P_*FDR*_* = 0.0054), and generalized epilepsy (FinnGen: OR: 1.1486; 95% CI: 1.0274–1.2842, *P_*FDR*_* = 0.0335; ILAE: OR: 1.0439; 95% CI: 1.0159–1.0728, *P_*FDR*_* = 0.0086). In contrast, no causal linkage of epilepsy on COVID-19 was observed. Further, FUMA analysis identified six overlapping genes, including *SMEK2*, *PNPT1*, *EFEMP1*, *CCDC85A*, *VRK2*, and *BCL11A*, shared between COVID-19 and epilepsy. Tissue-specific expression analyses revealed that the disease-gene associations of COVID-19 were significantly enriched in lung, ovary, and spleen tissue compartments, while being significantly enriched in brain tissue for epilepsy.

**Conclusion:**

Our study demonstrates that COVID-19 can be a contributing factor to epilepsy, but we found no evidence that epilepsy contributes to COVID-19.

## 1. Introduction

The global COVID-19 pandemic has left an indelible mark on the state of public health, economies, and societies. Although COVID-19 is primarily recognized as a respiratory disease, recent studies have emphasized that neurological conditions can be a significant consequence of the infection (Adhikari et al., 2020; Yusuf et al., 2021). At the outset of COVID-19, the most prevalent neurological manifestation involves symptoms such as headache, dizziness, and loss of smell or taste (Romoli et al., 2020). In severe COVID-19 cases, patients may develop prolonged neurological comorbidities such as sleep disturbances, seizures, encephalitis, stroke, Guillain-Barre syndrome, “brain fog,” or cognitive impairment, which can have long-lasting impacts on patient outcomes (Yu and Yu, 2020). This underscores the pressing need to fully comprehend the inherent causal association between COVID-19 and neurological disorders.

Epilepsy, a chronic neurological disorder characterized by recurring seizures, has emerged as a potential comorbidity of COVID-19. Nevertheless, the correlation between epilepsy and COVID-19 remains unclear and is currently under continuous research. Some studies have indicated a heightened risk of COVID-19 infection and more severe outcomes, such as hospitalization and death, for individuals with epilepsy, especially if accompanied by other underlying medical conditions (Siahaan et al., 2021; Muccioli et al., 2022). Conversely, other studies have failed to establish any significant link between COVID-19 and epilepsy (Granata et al., 2020; Kuroda, 2020).

Comprehending the causal correlation between epilepsy and COVID-19 is vital in order to devise efficacious interventions and enhance patient outcomes. Nevertheless, determining causation in observational studies is intricate owing to the existence of confounding factors, reverse causation, bias, and limiting causal inference. Moreover, since genes precede the onset of diseases, reverse causation issues are mitigated, and Mendelian randomization (MR) emerges as a potent technique to discern causal effects. MR is a statistical method that uses genetic variants as instrumental variables (IVs) through genome-wide association studies (GWAS) with both exposure and outcome to estimate causal effects in observational studies. Furthermore, by carefully controlling for potential confounding variables, MR facilitates informed clinical decision-making and evidence-based public health policies, which can effectively alleviate the burden of both conditions. Functional mapping and annotation (FUMA) offers a robust platform for examining the genetic architecture underpinning complex traits. The platform assists in delineating intricate relationships that exist between genetic variations and phenotypes (Liu et al., 2023). The objective of our investigation was to comprehensively examine the causal correlation between COVID-19 and epilepsy via a bidirectional two-sample MR analysis combined with FUMA analysis.

## 2. Materials and methods

### 2.1. Mendelian randomization

**FIGURE 1:**
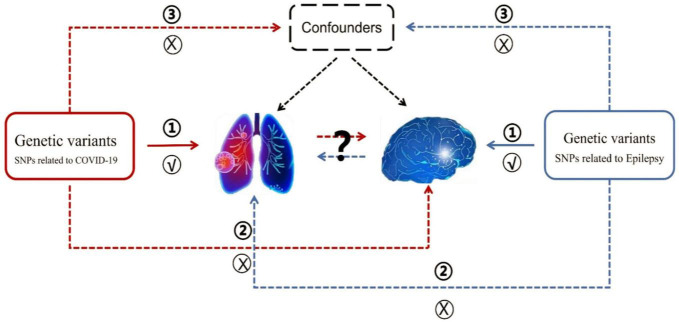
The overview of MR analysis in the present study. The forward MR analysis denoting the causal association of COVID-19 on epilepsy was represented by the red line label. Conversely, the reverse MR analysis denoting the causal association of epilepsy on COVID-19 was represented by the blue line label.

The summary datasets from GWAS were presented in Table 1. The COVID-19 GWAS dataset was provided by the FinnGen consortium, and the source of this valuable data is publicly available at https://www.finngen.fi/en/access results. The epilepsy GWAS datasets were from two sources: the FinnGen consortium (FinnGen released for R8) and the International League Against Epilepsy Consortium on Complex Epilepsies (2018). To determine causality, the MR analysis was required to meet three assumptions: (1) a strong correlation between genetic variation and the exposure factor (Association assumption), (2) the exposure factor is not directly related to the outcome (Exclusion assumption), and (3) genetic variation is independent of any confounding factors (Independence assumption). Given that the investigation employed publicly accessible GWAS summary data, ethical approval was not required (Carter et al., 2021). The MR framework design was shown in Figure 1.

**TABLE 1:** Detailed information of included GWAS datasets.

Phenotype	Sample size (cases/controls)	Population	Consortium	Year	Journal	References
COVID-19 infection	159,840/2,782,977	Mixed	FinnGen	2022	**–**	
COVID-19 hospitalization	44,986/2,356,386	Mixed	FinnGen	2022	**–**	
COVID-19 severity	18,152/1,145,546	Mixed	FinnGen	2022	**–**	**–**
Epilepsy	10,354/264,662	Mixed	FinnGen	2022	**–**	**–**
Focal epilepsy	1,132/332,145	Mixed	FinnGen	2022	**–**	**–**
Generalized epilepsy	1,988/332,145	Mixed	FinnGen	2022	**–**	**–**
Epilepsy	15,212/29,677	Mixed	ILAE (International League Against Epilepsy Consortium on Complex Epilepsies, 2014)	2018	Nat Commun	Abou-Khalil et al. (8) (PMID: **30531953)**
Focal epilepsy	9,671/29,677	Mixed	ILAE	2018	Nat Commun	Abou-Khalil et al. (8) (PMID: **30531953)**
Generalized epilepsy	3,769/29,677	Mixed	ILAE	2018	Nat Commun	Abou-Khalil et al. (8) (PMID: **30531953)**

### 2.2. Selection of IVs

This study conducted a Forward MR analysis using COVID-19 infection, hospitalization, and severity as exposures, with a *P*-value threshold of *P* < 5 × 10^–8^ using a screening variable tool. The reverse MR analysis utilized epilepsy and its subtypes as exposures, and to prevent potential bias resulting from a small number of SNPs generated by a *P*-value threshold of *P* < 5 × 10^–8^, a *P*-value threshold of *P* < 5 × 10^–6^ was set. SNPs in linkage disequilibrium (r^2^ threshold < 0.001 within 5,000 Kb) were excluded, and the Phenoscanner database was utilized to screen and remove SNPs that may act as confounding factors for the outcome^1^ (Staley et al., 2016).

The strength of the exposure proxy instrumental variables was verified by calculating the F statistic for each SNP and only retaining those with an F statistic greater than 10 to prevent bias from weak instrumental variables. The R^2^ value for each SNP was determined employing the formula *R*^2^ = 2 × EAF × (1-EAF) × β^2^, and formula for F statistic was *F* = ($\frac{N-K-1}{\text{K}}$) ($\frac{{\text{R}}^{2}}{1-{\text{R}}^{2}}$) (Brion et al., 2013).

### 2.3. Statistical analysis

The statistical analyses were carried out with R version 4.2.2 and the TwoSample MR (Hemani et al., 2018) and MR-PRESSO packages (Verbanck et al., 2018). To evaluate causality, five distinct methods were utilized: Inverse-variance weighted (IVW), MR-Egger, Weighted median, Simple mode, and Weighted mode. IVW, a meta-method that considers each SNP as a natural experiment and enforces the intercept in the regression slope to be zero, was chosen as the primary method to assess the correlation between exposure and outcome (Hartwig et al., 2017). The remaining four methods were used to complement the IVW results and provide more precise estimates under different conditions (Zheng et al., 2017). The degree of heterogeneity was evaluated by Cochran’s Q statistic and leave-one-out analysis. MR Egger and MR-PRESSO global tests were used to detect horizontal pleiotropy. The Benjamini-Hochberg method was utilized to adjust for multiple testing and compute the corrected P value for the false discovery rate (FDR). A raw *P* value < 0.05 and a *P*_*FDR*_ < 0.05 indicated a significant causal association, while a raw *P* value < 0.05 and a *P*_*FDR*_ > 0.05 suggested a potential association between exposure and outcome. The power calculations were carried out utilizing an online power calculator (mRnd), which can be found at https://shiny.cnsgenomics.com/mRnd/ (Brion et al., 2013).

### 2.4. Analysis of FUMA

Single nucleotide polymorphism-based tissue enrichment analysis, facilitated by FUMA, was employed to scrutinize phenotype related tissue specificity. This method utilizes gene-property analyses to investigate the correlation between GWAS hits and tissue-specific gene expression profiles (Watanabe et al., 2017). Risk loci are identified based on significant SNP associations, and the linkage disequilibrium (LD) structure is considered during gene prioritization using the SNP2GENE approach. Three distinct methodologies can be employed to map functionally annotated single nucleotide polymorphisms (SNPs) to genes. These encompass the utilization of positional mapping to evaluate the impact of SNPs on genes, conducting eQTL analysis to identify associations with gene expression, and employing chromatin interactions mapping in tissue type that are pertinent to the phenotype.

To investigate the overlap between COVID-19 and epilepsy gene sets, The GWAS findings for COVID-19 and epilepsy were procured from the reputable databases of FinnGen and ILAE Catalog, respectively. The resulting datasets were filtered to include only protein-coding genes, and the SuperExactTest R package was used to evaluate the overlap gene between the two GWAS datasets, with a total of 30,000 genes in the genome set (Hulleman and Olivers, 2017). Using SNP2GENE, positional mapping was performed within a maximum distance of 10 kb, along with 3D Chromatin Interaction mapping and cis-eQTL mapping using GTEx v8 of Brain and Lung, with default settings for both SNP2GENE and GENE2FUNC. A threshold of 5 × 10^–8^ was set for the maximum *P*-value of the lead SNPs, while an r^2^ threshold of 0.6 was used to identify independent significant SNPs. The gene-set enrichment analysis was adjusted for multiple testing using the FDR.

## 3. Results

### 3.1. The causal effect of COVID-19 on epilepsy

To investigate the causal linkage of COVID-19 on epilepsy, three COVID-19 traits (infection, hospitalization, and severity) were used as exposures, and epilepsy from FinnGen GWAS database acted as outcome, with 8, 15, and 6 SNPs included for each trait, respectively. The infection (OR 1.5306, 95% confidence interval [CI]: 1.1676–2.0062, *P* = 0.0021, power = 97%), hospitalization (OR 1.0934, 95% CI: 1.0097–1.1841, *P* = 0.0281, power = 63%), and severity (OR 1.2454, 95% CI: 1.0850–1.4295, *P* = 0.0018, power = 93%) of COVID-19 exhibited significant genetic correlation with epilepsy (Tables 2, 3). After further FDR adjustment by Benjamini-Hochberg method, there was still a significant *P*-value for the causal association of COVID-19 infection (*P_*FDR*_* = 0.0076), hospitalization (*P_*FDR*_* = 0.0422), and severity (*P_*FDR*_* = 0.0162) on epilepsy (Table 2). For the infection and severity of COVID-19, the power value was 97 and 93%, respectively, which were highly significant, suggesting that our MR research results are highly reliable (Table 3). Although the power value of the causal relationship between the hospitalization of COVID-19 and epilepsy was 63%, which was slightly less significant, the convincing power is still above the middle level. Leave-one-out was used to test the single SNP effect, and no SNP was found that could lead to significant changes in the results (Supplementary Figures 1A–C). The weighted median indicated consistent results for the causal association of COVID-19 infection (OR 1.4247, 95% CI: 1.0155–1.999, *P* = 0.0404) and COVID-19 severity (OR 1.2076, 95% CI: 1.0146–1.4374, *P* = 0.0338) on epilepsy (Supplementary Table 1).

**TABLE 2:** Main results of MR analysis.

Exposure	Outcome	nSNPs	Method	OR (95% CI)	*P*-value	P_FDR_
**Epilepsy and its subtypes database from FinnGen consortium**						
COVID-19 (infection)	Epilepsy	8	IVW	1.5306 (1.1676, 2.0062)	0.0021	0.0076
	Focal epilepsy	6	IVW	1.0161 (0.6269, 1.498)	0.1931	0.1931
	Generalized epilepsy	9	IVW	2.1155 (1.1734, 3.8139)	0.0127	0.0327
COVID-19 (hospitalization)	Epilepsy	15	IVW	1.0934 (1.0097, 1.1841)	0.0281	0.0422
	Focal epilepsy	9	IVW	1.5846 (1.0099, 2.4863)	0.0452	0.0542
	Generalized epilepsy	20	IVW	1.2281 (1.0513, 1.4346)	0.0621	0.0699
COVID-19 (severity)	Epilepsy	6	IVW	1.2454 (1.0850, 1.4295)	0.0018	0.0162
	Focal epilepsy	7	IVW	1.6818 (1.1478, 2.4642)	0.0077	0.0231
	Generalized epilepsy	20	IVW	1.1486 (1.0274, 1.2842)	0.0149	0.0335
**Epilepsy and its subtypes database from ILAE**						
COVID-19 (infection)	Epilepsy	3	IVW	1.3440 (1.0235,1.7649)	0.0334	0.0429
	Focal epilepsy	3	IVW	1.0869 (0.9715, 1.2159)	0.1455	0.1541
	Generalized epilepsy	4	IVW	1.1245 (1.0444, 1.2108)	0.0019	0.0114
COVID-19 (hospitalization)	Epilepsy	7	IVW	1.7381 (1.0467, 2.8862)	0.0326	0.0451
	Focal epilepsy	8	IVW	1.7549 (1.1063, 2.7838)	0.0169	0.0338
	Generalized epilepsy	8	IVW	1.1827 (1.0215, 1.3693)	0.0248	0.0406
COVID-19 (severity)	Epilepsy	8	IVW	1.2724 (1.0347, 1.5647)	0.0224	0.0403
	Focal epilepsy	6	IVW	1.6598 (1.2572, 2.1914)	0.0003	0.0054
	Generalized epilepsy	6	IVW	1.0439 (1.0159, 1.0728)	0.0019	0.0086

CI, confidence interval; FDR, false discovery rate; IVW, inverse-variance weighted; nSNPs, number of single-nucleotide polymorphisms; OR, odds ratio.

**TABLE 3:** Power calculation for two-sample MR analysis of COVID-19 on epilepsy.

Exposure	Outcome	Sample size	Proportion of cases	OR	R^2^	Power
**Epilepsy and its subtypes database from FinnGen consortium**						
COVID-19 (infection)	Epilepsy	275,016	0.0376	1.5306	0.0053	97%
	Focal epilepsy	333,277	0.0034	2.5161	0.0019	60%
	Generalized epilepsy	334,133	0.0059	2.1155	0.0056	96%
COVID-19 (hospitalization)	Epilepsy	275,016	0.0376	1.0934	0.0609	63%
	Focal epilepsy	333,277	0.0034	1.5846	0.0174	73%
	Generalized epilepsy	334,133	0.0059	1.2077	0.0795	74%
COVID-19 (severity)	Epilepsy	275,016	0.0376	1.2454	0.0200	93%
	Focal epilepsy	333,277	0.0034	1.6818	0.0234	94%
	Generalized epilepsy	334,133	0.0059	1.1486	0.1695	77%
**Epilepsy and its subtypes database from ILAE**						
COVID-19 (infection)	Epilepsy	44,889	0.3389	1.3440	0.0013	20%
	Focal epilepsy	39,348	0.2458	1.0869	0.0013	6%
	Generalized epilepsy	33,446	0.1127	1.1245	0.0025	6%
COVID-19 (hospitalization)	Epilepsy	44,889	0.3389	1.7381	0.0188	100%
	Focal epilepsy	39,348	0.2458	1.7549	0.0208	100%
	Generalized epilepsy	33,446	0.1127	1.1827	0.0147	24%
COVID-19 (severity)	Epilepsy	44,889	0.3389	1.2724	0.0419	100%
	Focal epilepsy	39,348	0.2458	1.6598	0.0334	100%
	Generalized epilepsy	33,446	0.1127	1.0439	0.0330	7%

The causal association was further verified by using the GWAS database of the ILAE consortium, and 3, 7, and 8 SNPs for the infection, hospitalization, and severity of COVID-19 were included, respectively. Consistent with the results from the FinnGen GWAS database, the IVW results showed statistically significant risk causal effects of the infection (OR 1.3440, 95% CI: 1.0235–1.7649, *P* = 0.0334, *P_*FDR*_* = 0.0429, power = 20%), hospitalization (OR 1.7381, 95% CI: 1.0467–2.8862, *P* = 0.0326, *P_*FDR*_* = 0.0451, power = 100%), and severity of COVID-19 (OR 1.2724, 95% CI: 1.0347–1.5647, *P* = 0.0224, *P_*FDR*_* = 0.0403, power = 100%) on epilepsy (Tables 2, 3). Moreover, no abnormal SNP was found using the leave-one-out sensitivity analysis (Supplementary Figures 2A–C). Furthermore, Table 3 did not reveal any evidence of heterogeneity or pleiotropy.

### 3.2. The causal effect of COVID-19 on focal epilepsy

Subsequently, we performed reverse MR to investigate the causal association of COVID-19 on focal epilepsy. In particular, 6 and 3 SNPs were included in the analysis for COVID-19 infection, 9 and 8 SNPs for COVID-19 hospitalization, and 7 and 6 SNPs for COVID-19 severity from FinnGen and ILAE GWAS, respectively. Results revealed a significant risk correlation between COVID-19 hospitalization and focal epilepsy for both FinnGen GWAS data (OR 1.5846, 95% CI: 1.0099–2.4863, *P* = 0.0452, *P_*FDR*_* = 0.0542, power = 73%) and ILAE GWAS data (OR 1.7549, 95% CI: 1.1063–2.7838, *P* = 0.0169, *P_*FDR*_* = 0.0338, power = 100%) (Tables 2, 3). Similarly, a significant risk correlation was found between COVID-19 severity and focal epilepsy for both FinnGen GWAS data (OR 1.6818, 95% CI: 1.1478–2.4642, *P* = 0.0077, *P_*FDR*_* = 0.0231, power = 94%) and ILAE GWAS data (OR 1.6598, 95% CI: 1.2572–2.1914, *P* = 0.0003, *P_*FDR*_* = 0.0054, power = 100%) (Tables 2, 3). The study utilized leave-one-out to test the single SNP effect and confirmed that no SNP led to significant changes in the results (Supplementary Figures 3A–C, 4A–C). There was no supporting evidence found to suggest a causal relationship between COVID-19 infection and focal epilepsy, as observed in both FinnGen GWAS data (OR 1.0161, 95% CI: 0.6269–1.498, *P* = 0.1931, *P_*FDR*_* = 0.1931, power = 60%) and ILAE GWAS data (OR 1.0869, 95% CI: 0.9715–1.2159, *P* = 0.1455, *P_*FDR*_* = 0.1541, power = 6%) (Tables 2, 3). No heterogeneity or pleiotropy was assessed in Table 4.

**TABLE 4:** Heterogeneity and pleiotropy tests for the associations of COVID-19 and epilepsy.

Exposure	Outcome	Cochrane’s *Q* test		MR-Egger intercept test		MRPRESSO global test
		*Q*-value	PQ	Intercept	P intercept	*P*-value
**Epilepsy and its subtypes database from FinnGen consortium**						
COVID-19 (infection)	Epilepsy	4.4546	0.7262	0.0059	0.5916	0.779
	Focal epilepsy	0.6799	0.9840	−0.1019	0.4834	0.991
	Generalized epilepsy	6.1334	0.6322	−0.0113	0.6139	0.733
COVID-19 (hospitalization)	Epilepsy	10.9421	0.6157	−0.0013	0.8485	0.758
	Focal epilepsy	3.1552	0.9242	0.0089	0.8860	0.932
	Generalized epilepsy	14.2134	0.7151	−0.0139	0.3521	0.785
COVID-19 (severity)	Epilepsy	2.9090	0.8202	0.0010	0.9623	0.846
	Focal epilepsy	3.8239	0.7005	0.0327	0.6886	0.726
	Generalized epilepsy	21.0657	0.3332	−0.0097	0.5188	0.419
**Epilepsy and its subtypes database from ILAE**						
COVID-19 (infection)	Epilepsy	1.2279	0.5412	0.0720	0.7376	-
	Focal epilepsy	4.3663	0.1127	−0.0133	0.3073	-
	Generalized epilepsy	1.0169	0.7972	−0.0001	0.9898	0.89
COVID-19 (hospitalization)	Epilepsy	3.1847	0.7853	−0.0341	0.6057	0.81
	Focal epilepsy	2.8059	0.9024	0.0031	0.7369	0.937
	Generalized epilepsy	12.7852	0.0775	−0.0073	0.7136	0.267
COVID-19 (severity)	Epilepsy	11.8500	0.1056	0.0102	0.7780	0.107
	Focal epilepsy	3.0074	0.6989	0.0147	0.8210	0.801
	Generalized epilepsy	3.4664	0.6285	−0.0025	0.6096	0.807

### 3.3. The causal effect of COVID-19 on generalized epilepsy

We investigated the causal effect of COVID-19 on generalized epilepsy, and used epilepsy from the FinnGen GWAS database as the outcome. A total of 9, 20, and 20 SNPs for COVID-19 infection, hospitalization, and severity were incorporated, respectively. The results indicated risk causal effects of COVID-19 infection (OR 2.1155, 95% CI: 1.1734–3.8139, *P* = 0.0127, *P_*FDR*_* = 0.0327, power = 96%) and severity (OR 1.1486, 95% CI: 1.0274–1.2842, *P* = 0.0149, *P_*FDR*_* = 0.0335, power = 77%) on generalized epilepsy. No evidence showed causal link between COVID-19 hospitalization and generalized epilepsy (OR 1.2281, 95% CI: 1.0513–1.4346, *P* = 0.0621, *P_*FDR*_* = 0.0699, power = 74%) (Tables 2, 3).

To validate our findings, an MR analysis using ILAE GWAS data as conducted. For the three COVID-19 traits, we incorporated 4, 8, and 6 SNPs correspondingly. It was identified that the genetically determined liability to COVID-19 infection (OR 1.1245, 95% CI: 1.0444–1.2108, *P* = 0.0019, *P_*FDR*_* = 0.0114, power = 6%), hospitalization (OR 1.1827, 95% CI: 1.0215–1.3693, *P* = 0.0248, *P_*FDR*_* = 0.0406, power = 24%) and severity (OR 1.0439, 95% CI: 1.0159–1.0728, *P* = 0.0019, *P_*FDR*_* = 0.0086, power = 7%) confers causal effects on generalized epilepsy (Tables 2, 3). Moreover, by using Leave-one-out to test single SNP effect, no SNP that significantly altered the results was found (Supplementary Figures 5A–C, 6A–C). Additionally, neither heterogeneity nor pleiotropy was assessed as shown in Table 3.

### 3.4. The causal effect of epilepsy and its subtypes on COVID-19

For the reverse MR analysis, there is no evidence to suggest any causal relationship of epilepsy and its subtypes on the three traits of COVID-19 outcome (Supplementary Tables 2, 3). In the sensitivity analysis using epilepsy data from the FinnGen GWAS to assess the causal association between generalized epilepsy and COVID-19 severity, heterogeneity was observed (Cochrane’s Q test: *P*_*Q*_ = 0.0428) (Supplementary Table 4). The power calculation was available in Supplementary Table 5. The specific characteristics and F value of selected SNPs were presented in Supplementary Tables 6, 7. In addition, by using Scatterplot, Forest plot and Funnel plot to test single SNP effect (Supplementary Figures 7–24).

### 3.5. The results of post-genome-wide association study annotation by functional mapping and annotation

As a professional epilepsy database, the ILAE GWAS database is more proficient in disease diagnosis and classification. Despite having a smaller total sample size than the FinnGen consortium, the case accounts for a larger proportion of the total population sample size. Therefore, we believe that the FUMA analysis should be conducted using the ILAE consortium database to obtain more accurate results.

**FIGURE 2:**
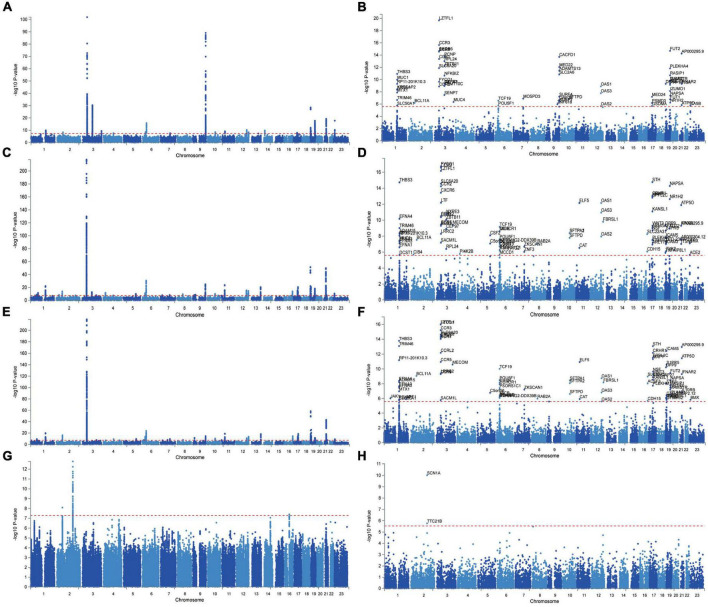
Manhattan Plot of GWAS summary statistics and Gene-based testing using MAGMA based on input GWAS summary statistics for essential hypertension. Red lines indicate levels of genome-wide significance (−log10P). COVID-19 infection **(A,B)**, COVID-19 hospitalization **(C,D)**, COVID-19 severity **(E,F)**, epilepsy **(G,H)**.

**FIGURE 3:**
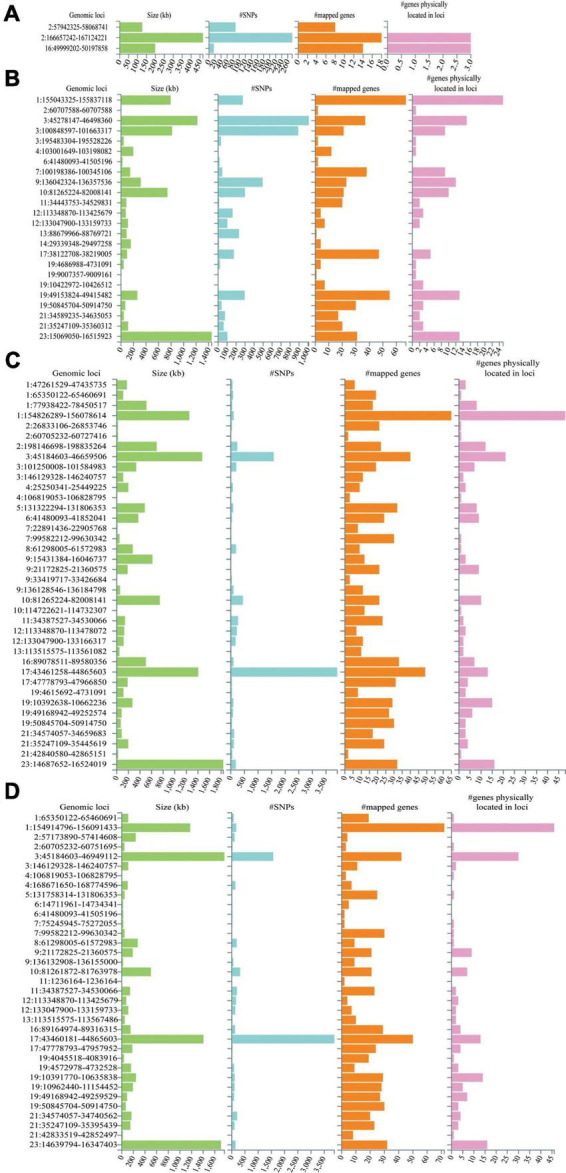
Genetic risk loci identified by FUMA analysis. **(A)** Genetic risk loci for epilepsy. **(B)** Genetic risk loci for COVID-19 infection. **(C)** Genetic risk loci for COVID-19 hospitalization. **(D)** Genetic risk loci for COVID-19 severity. Genomic risk loci are displayed in the format of “chromosome:start position–end position.” Each genomic locus is represented by a series of histograms, arranged from left to right to display the size of the locus, the number of candidate SNPs, the number of mapped genes, and the number of known genes located within it.

**FIGURE 4:**
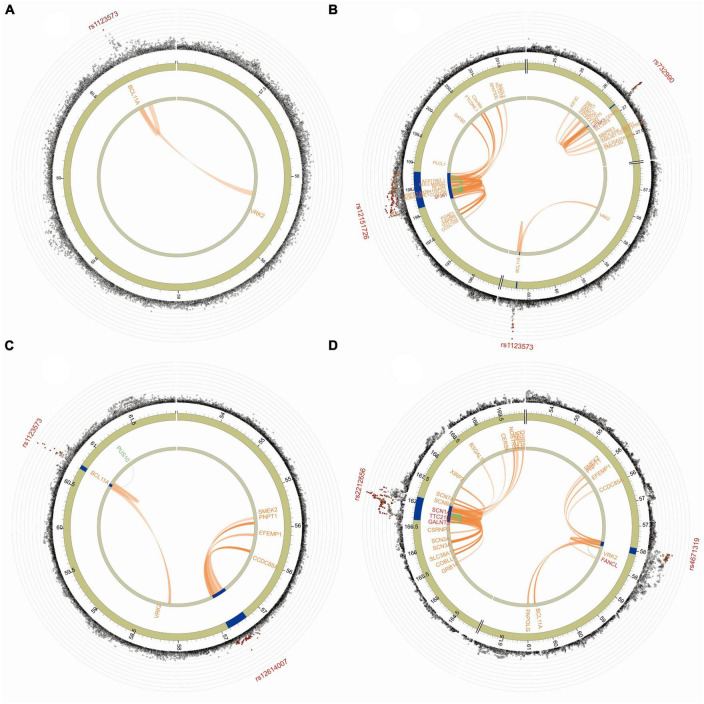
The functional annotation of three COVID-19 traits. **(A)** The functional annotation of COVID-19 infection. **(B)** The functional annotation of COVID-19 hospitalization. **(C)** The functional annotation of COVID-19 severity. **(D)** The functional annotation of epilepsy. The outer circle of the GWAS plot displays SNP associations (gray circles) with -log10(*p*-value). The lead SNP is labeled, with rs1123573, rs732990, rs12151726, and rs12614007 in COVID-19 and rs2212656 and rs4671319 in epilepsy. In the Manhattan plot, only SNPs with *P* < 0.05 are displayed. SNPs in genomic risk loci are color-coded based on their maximum r2 to the independent significant SNPs in the locus, with red (*r*^2^ > 0.8), orange (*r*^2^ > 0.6), green (*r*^2^ > 0.4), and blue (*r*^2^ > 0.2). SNPs that are not in linkage disequilibrium with any of the independent significant SNPs (with *r*^2^ ≤ 0.2) are gray. The chromosome ring, the second layer of the Circos plot, highlights genomic risk loci in blue. The third layer displays mapped genes by chromatin interactions or eQTLs, colored orange or green, respectively. The same is true of the second layer, but without coordinates to align the position of genes with genomic coordinate. Links colored orange represent chromatin interactions, green are eQTLs, and blue if both a chromatin interaction and an eQTL.

The Manhattan plot of the input GWAS summary statistics and gene-based test as computed by MAGMA were provided in Figure 2. A total of 581 independent SNPs with significance and 182 lead SNPs were identified, along with 97 genomic risk loci (Figures 3B–D; Supplementary Table 8), for COVID-19. Similarly, 10 independent SNPs with significance, 4 lead SNPs, and 3 genomic risk loci (Figure 3A; Supplementary Table 9) were identified for epilepsy. Additionally, 1,849 protein-coding risk genes were identified for COVID-19 (Supplementary Table 10) and 40 for epilepsy (Supplementary Table 11). Of these, 6 genes were found to be shared between COVID-19 and epilepsy, including *SMEK2*, *PNPT1*, *EFEMP1*, *CCDC85A*, *VRK2*, and *BCL11A*, all located on chromosome 2 (Figure 4). It was identified that *PNPT1*, *EFEMP1*, *CCDC85A*, *VRK2*, and *BCL11A* were expressed in the brain, with the down-regulation of *EFEMP1* and *VRK2*, and the up-regulation of *PNPT1*, *CCDC85A*, and *BCL11A*.

**FIGURE 5:**
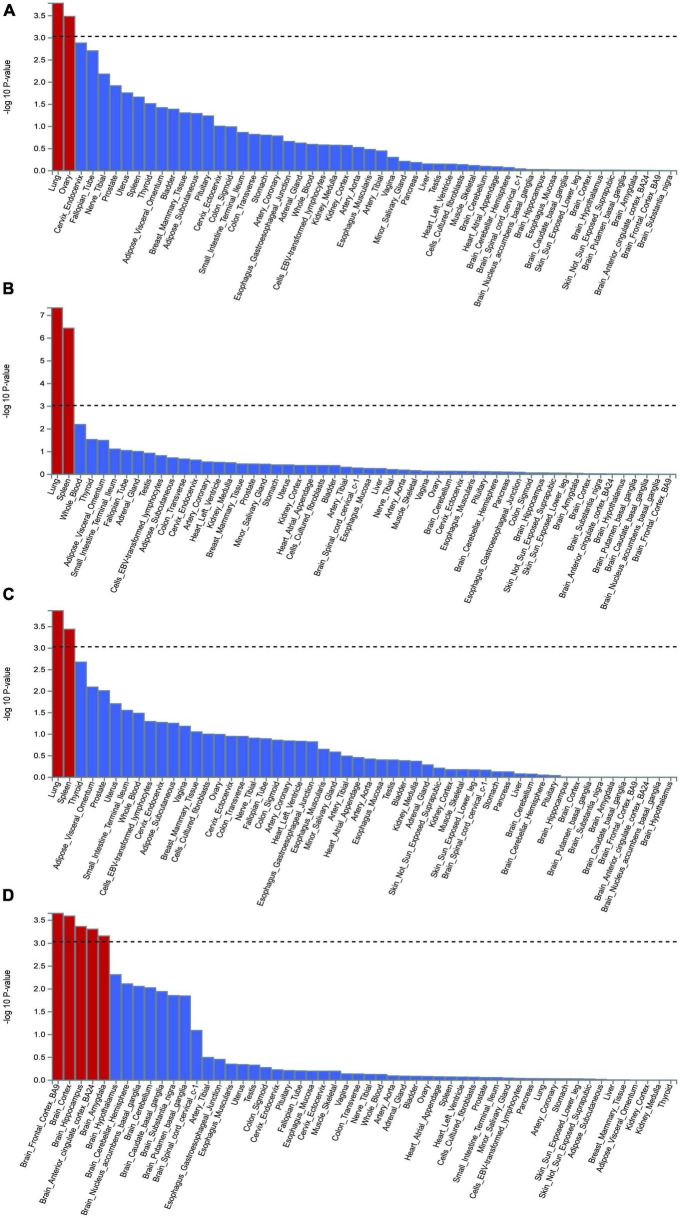
The analysis of tissue enrichment by FUMA using GTEx v8 (*n* = 54 tissues). **(A)** Tissue enrichment for COVID-19 infection. **(B)** Tissue enrichment for COVID-19 hospitalization. **(C)** Tissue enrichment for COVID-19 severity. **(D)** Tissue enrichment for epilepsy.

MAGMA gene-based testing and GWAS summary statistics were used to identify significant genes, which were plotted on a Manhattan map based on the COVID-19 and epilepsy GWAS databases. The analysis of tissue-specific gene expression demonstrated that the disease-gene associations of COVID-19 infection were notably concentrated in the lung and ovary tissue compartments, while COVID-19 hospitalization and severity showed significant enrichment in the lung and spleen tissue compartments. Although no substantial enrichment was detected in the brain tissue, our findings revealed that the disease-gene associations for COVID-19 infection were enriched in other regions of the brain, including the pituitary, brain cerebellum, brain cerebellar hemisphere, brain-spinal-cord-cervical-c-1, brain hippocampus, brain cortex, and brain hypothalamus (Figures 5A–C). Furthermore, the disease-gene associations linked to epilepsy were considerably enriched in numerous brain regions such as the brain frontal cortex-BA9, brain hippocampus, brain cortex, brain amygdala, and brain anterior cingulate cortex-BA24 (Figure 5D).

## 4. Discussion

Our study utilized a rigorous and sophisticated statistical methodology, known as bidirectional MR analysis, to comprehensively investigate the potential causal association between COVID-19 and epilepsy. Our findings revealed that infection, hospitalization, and severity of COVID-19 exhibited a causal relationship with an increased risk of epilepsy. This highlights the potential significance of these traits in the development of epilepsy following COVID-19 infection. To further validate our results, we conducted a series of sensitivity analyses based on distinct underlying assumptions, which consistently yielded similar results. These findings demonstrate the robustness and reliability of our results and contribute to a better understanding of the potential neurological sequelae of COVID-19 infection.

The results of a multicenter retrospective cohort study conducted by Harvard Medical School showed that electrographic seizures can serve as autonomous predictors of in-hospital mortality among COVID-19 patients, and epileptiform abnormalities could act as robust indicators of a prolonged hospital stay (Lin et al., 2021). Subclinical or electrographic seizures were frequently observed in patients who are hospitalized with critical illnesses (Claassen et al., 2004), and have been revealed to be linked to unfavorable outcomes (Abend et al., 2013). Nonetheless, it is crucial to consider that such results could be confounded by the severity of COVID-19, which is frequently characterized by hypoxia, multi-organ failure, metabolic disruptions, and brain injury resulting from cerebrovascular events or anoxic brain injury, all of which may have influenced the outcomes observed in the study (Lin et al., 2021). Currently, some studies have provided the conflicting results. A retrospective multicenter study conducted in China reported no instances of acute symptomatic clinical seizures or status epilepticus in 304 COVID-19 patients without a past history of epilepsy (Lu et al., 2020). However, absence of EEG detection in that study warrants a cautious interpretation of the results. Meanwhile, a meta-analysis of 39 studies involving 68,362 COVID-19 patients indicated that approximately 21% of patients had neurological symptoms, while epileptic seizures occurred in only 0.7% of patients (Yoo et al., 2022). To mitigate the impact of potential confounding variables, we employed the MR approach by considering the infection, hospitalization, and severity of COVID-19 as exposures, which allowed us to elucidate the association between these three traits of COVID-19 and epilepsy, as well as the subtypes of focal and generalized epilepsy. Our study yielded more robust findings than prior retrospective studies, demonstrating a positive causal relationship between increased COVID-19 infection, hospitalization, and severity, and a higher incidence of epilepsy and generalized epilepsy outcome. Although we did not detect a causal link between COVID-19 infection and focal epilepsy, our analysis of data from both the ILAE and FinnGen Consortium revealed that increased COVID-19 hospitalization and severity were positively associated with high risks of focal epilepsy. Given the demonstrated effectiveness of vaccination in reducing the incidence of severe COVID-19 (Rahmani et al., 2022), our findings have the potential to inform recommendations for vaccination strategies aimed at mitigating the risk of developing epilepsy.

It is essential to note that COVID-19 is not the only infectious disease that has been correlated with epilepsy. Historically, various infectious diseases, including acute bacterial meningitis, intracranial abscesses, intracranial empyemas, CNS tuberculosis, neurocysticercosis, cerebral malaria, onchocerciasis have been associated with an increased risk of epilepsy (Vezzani et al., 2016). The exact mechanisms through which these infections lead to epilepsy can vary, but they often involve direct brain infection or post-infectious immune responses. The underlying mechanism of epilepsy complication of COVID-19 is far from known. Several potential mechanisms have been suggested for the occurrence of epilepsy following COVID-19, including neuroinflammation, blood-brain barrier breakdown, abnormal coagulation, stoke, mitochondria disorder, and electrolytes imbalance (Nikbakht et al., 2020). FUMA analysis identified six genomic risk loci containing overlapping protein-coding genes for both COVID-19 and epilepsy, indicating a potential shared pathophysiology between these two diseases. Among the six shared genes, *SMEK2*, *PNPT1*, *EFEMP1*, *CCDC85A*, *VRK2*, and *BCL11A*, all but *SMEK2* were found to be expressed in the brain. *PNPT1*, which encodes a key enzyme in mitochondrial RNA metabolism, is an interesting candidate as loss of its activity can result in mitochondrial dysfunction, triggering a Type I interferon response and an initial inflammatory response (Dhir et al., 2018). The *VRK2* gene, primarily expressed in the brain, functions as a mediator of signaling pathways that regulate tumor cell growth and apoptosis. Mutations in *VRK2* have been associated with genetic generalized epilepsy (Steffens et al., 2012; International League Against Epilepsy Consortium on Complex Epilepsies, 2014). Since apoptosis plays a crucial role in the development of both COVID-19 and epilepsy (Henshall and Simon, 2005; Aghagoli et al., 2021), *VRK2* may contribute to the disease mechanism through its apoptosis mechanism. The leucocyte differentiation gene *BCL11A* encodes for a regulatory zinc-finger protein that can form a protein complex with *CASK* to regulate axon outgrowth and branching (Kuo et al., 2009; Kuo et al., 2010). *BCL11A* is regarded as a potential therapeutic target for epilepsy (Wang et al., 2022)and was identified in a study in the UK as an independent variant that significantly predisposes individuals to critical COVID-19 (Kousathanas et al., 2022). While the impact of *SMEK2*, *PNPT1*, *EFEMP1*, *CCDC85A*, *VRK2*, and *BCL11A* risk genes on the causal relationship between COVID-19 and epilepsy remains uncertain, this study’s findings offer valuable insights for future mechanistic research, as these genes could be involved in the shared genetic and etiological basis of the two conditions.

The susceptibility of individuals with epilepsy to COVID-19 and the potential for them to experience worse outcomes remains ambiguous. A study reported that only 14 patients were tested positive for COVID-19 among 5,700 patients with epilepsy, and Out of the 2,122 patients who were admitted to COVID-19 units, none of them experienced seizures as an early symptom (Granata et al., 2020). According to a large-scale study conducted in Iran that involved 37,968 COVID-19 patients, only a scant 0.2% of patients had pre-existing epilepsy, and no significant disparities between patients with and without pre-existing epilepsy concerning intubation, ICU care, or mortality were detected, suggesting that individuals with epilepsy may not be at a greater risk of contracting COVID-19 or experiencing more severe illness or worse prognosis (Asadi-Pooya et al., 2021). However, these findings should be interpreted with caution, as they may be influenced by the substantial obstacles that patients with epilepsy face in obtaining medication and care in various ways, despite the implementation of telehealth guidance during the pandemic (Rasmusson and Hartshorn, 2005; Mueller et al., 2021; Pellinen and Holmes, 2022). Additionally, the constrained availability of Electroencephalogram (EEG), particularly in epilepsy monitoring units, during the pandemic has compelled the exploration of alternative diagnostic methods, which may have implications for the precise diagnosis of epilepsy and its subtypes (Freund and Feyissa, 2022). In this study, we utilized epilepsy and its subtypes, including focal and generalized epilepsy, as the exposure variables to investigate the potential causal effect of epilepsy on COVID-19 using the MR method. Our findings suggest that epilepsy does not have a significant causal association with the infection, hospitalization, and severity of COVID-19. Conversely, one study reported that the cumulative incidence of COVID-19 in active epilepsy patients was higher compared to population without epilepsy, and active epilepsy was identified as an independent risk factor for mortality in COVID-19 hospitalized patients (Cabezudo-García et al., 2020). Noteworthy, the results interpretation in that study should only be accepted with caution as the patients with active epilepsy included 12 possible/probable cases, accounting for 57% (Cabezudo-García et al., 2020). Additionally, patients with epilepsy have a higher prevalence of risk factors associated with more severe disease, such as age, comorbidities with hypertension, stroke, diabetes and obesity, as well as immunosuppression, respiratory issues, institutionalized living (Asadi-Pooya et al., 2021), which could elucidate the reason behind the increased prevalence of COVID-19 among individuals with epilepsy.

Despite the significant contributions of our study, several limitations should be acknowledged. Foremost, our study primarily focused on individuals of European ancestry, necessitating careful consideration when extrapolating our findings to other ethnic groups. Additionally, to maintain the independence assumption, we implemented rigorous selection criteria to eliminate SNPs that may be linked with potential confounding factors of both COVID-19 and epilepsy, potentially compromising the statistical power of our analysis. Furthermore, heterogeneity in generalized epilepsy severity in the ILAE dataset could be attributed to its diverse population. Since both Finngen and ILAE datasets included mixed populations, the frequency variations of genetic variants used as IVs across different ethnic or geographic populations may impact the validity of our results due to population stratification. Further, due to the lack of individual-level data, we encountered difficulties in precisely addressing the likelihood of sample overlap between COVID-19 and epilepsy GWAS datasets, raising the possibility of bias in the overall estimates. Lastly, we assessed genetic liability alone without accounting for environmental influences, which play a crucial role in both epilepsy and COVID-19.

## 5. Conclusion

This study stands out as the first to employ bidirectional MR analysis, and identified causal effects of COVID-19 infection, hospitalization and severity on a higher risk of epilepsy. Regardless of the precise mechanism underlying the associations through a genetic background, our research offers new understanding into the causal relationship between COVID-19 and epilepsy.

## Data availability statement

The original contributions presented in this study are included in the article/Supplementary material, further inquiries can be directed to the corresponding author.

## Author contributions

MY and PY drafted the manuscript. LL, BL, and ZP performed data analysis and data interpretation. HX provided useful advice on the design of this study and supervised the competition of this work. All authors approved the manuscript for publication.

## Footnotes

1
http://www.phenoscanner.medschl.cam.ac.uk/
