# Supplementary material for: The association between epilepsy and COVID-19: analysis based on Mendelian randomization and FUMA
Source: Front Neurosci. 2023 Sep 15;17:1235822. doi: 10.3389/fnins.2023.1235822 (PMC10540302; doi:10.3389/fnins.2023.1235822)
Supplement: Supplementary file 1 [file Data_Sheet_1.ZIP › Supplementary Materials:Fig. S1-24.docx]

**Figure. S1** Leave one out plot. The causal effect of COVID-19 infection (A), COVID-19 hospitalization (B), COVID-19 severity(C) on epilepsy (FinnGen).


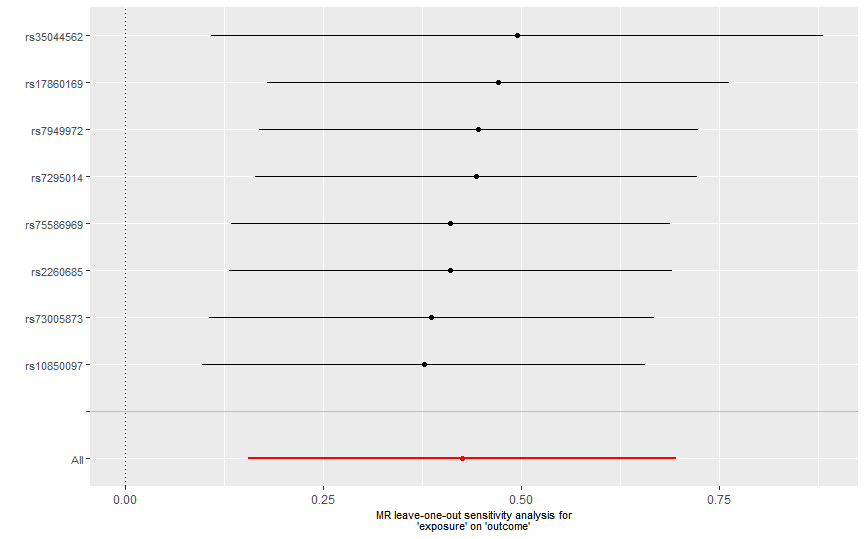

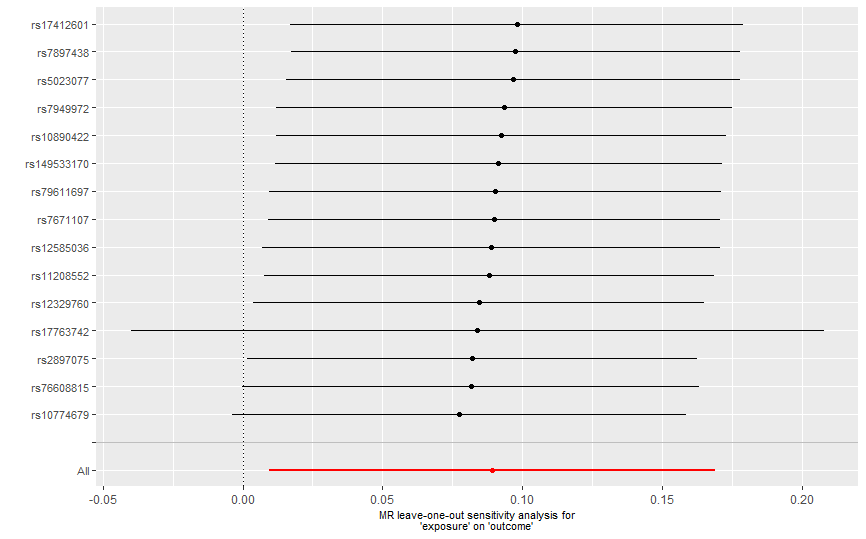

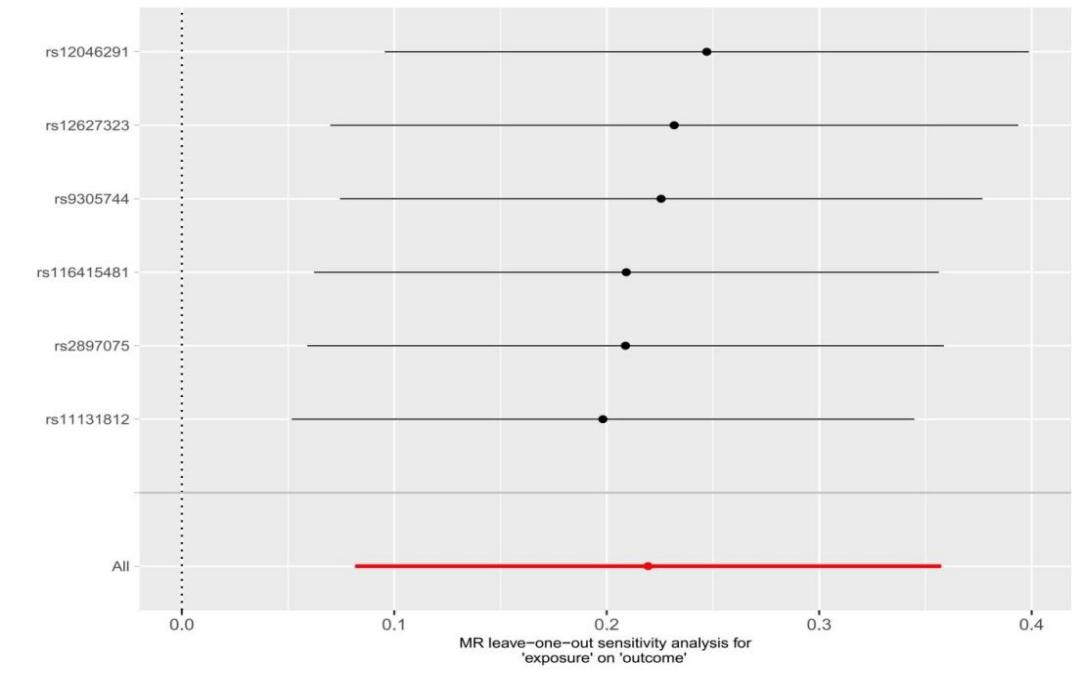


A

B

C

**Figure. S2** Leave one out plot. The causal effect of COVID-19 infection (A), COVID-19 hospitalization (B), COVID-19 severity(C) on epilepsy (ILAE).


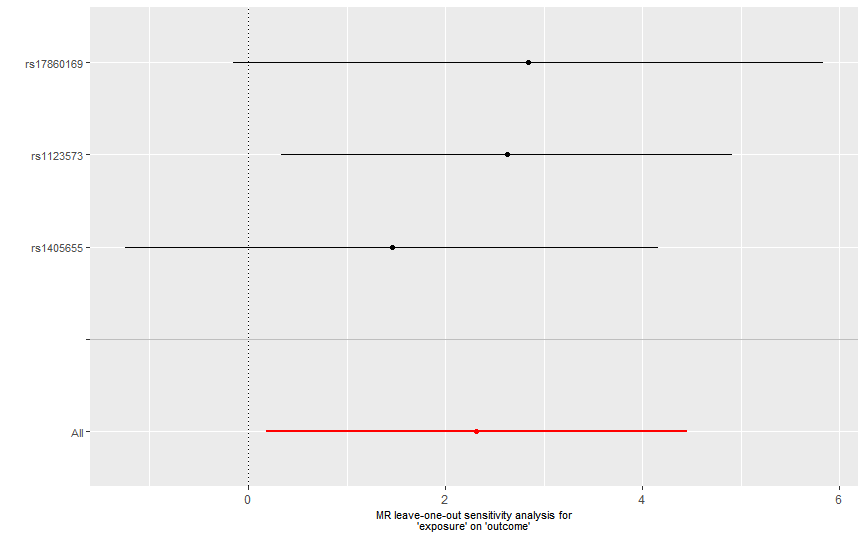

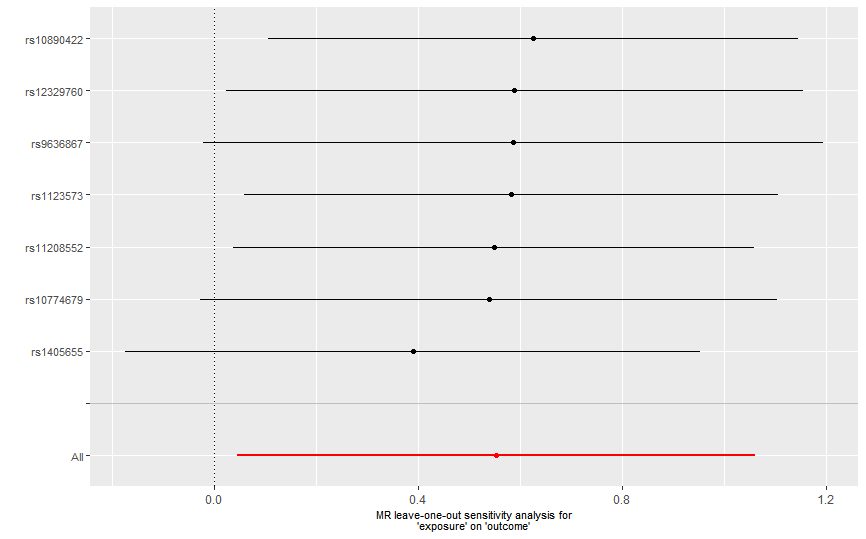


A

B

C


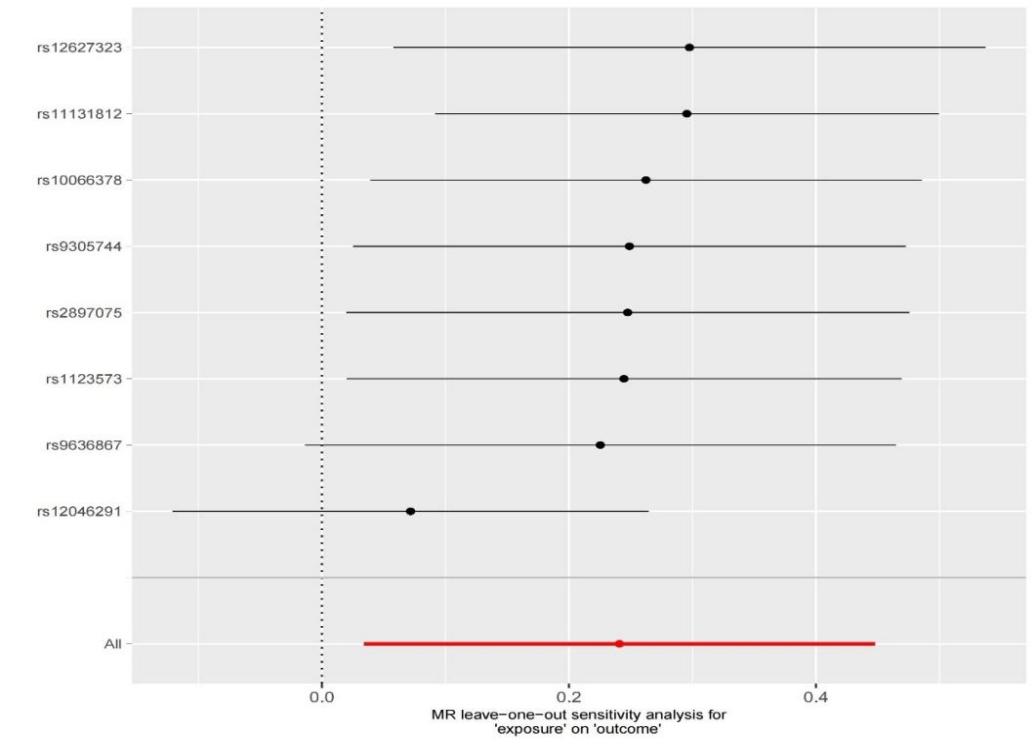


**Figure. S3** Leave one out plot. The causal effect of COVID-19 infection (A), COVID-19 hospitalization (B), COVID-19 severity(C) on focal epilepsy (FinnGen).


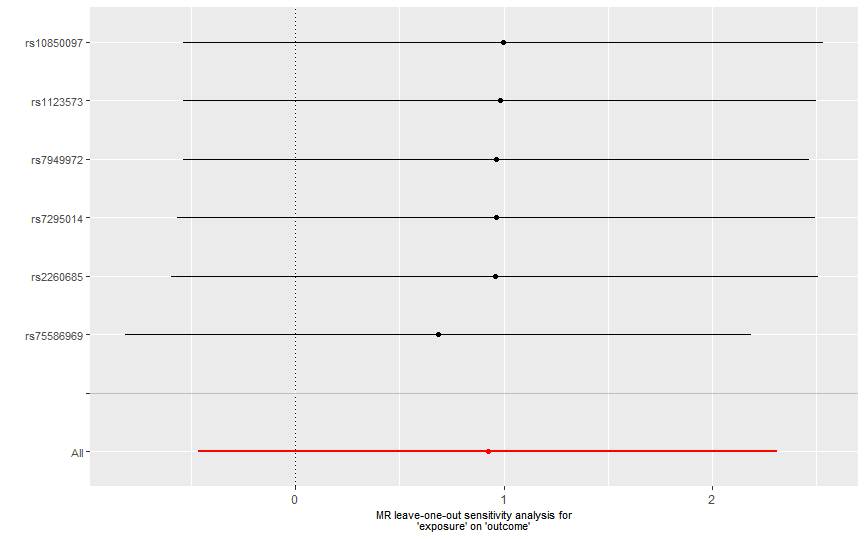

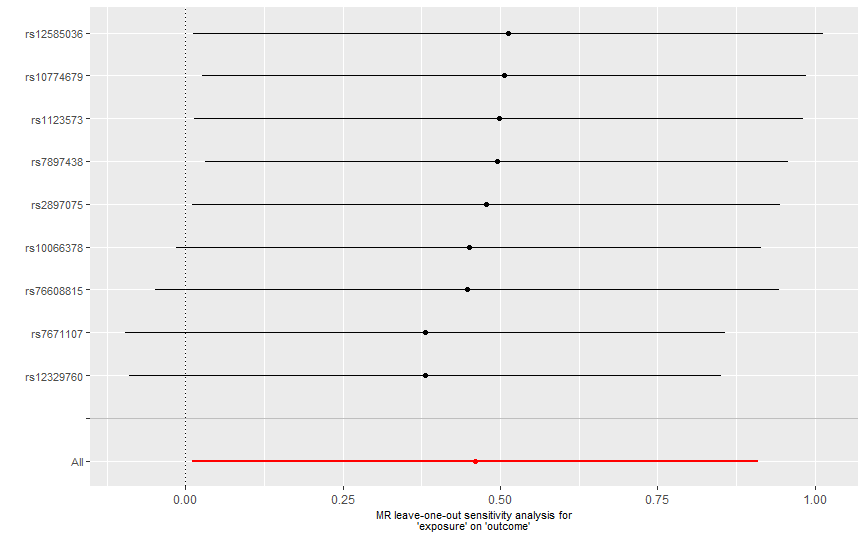

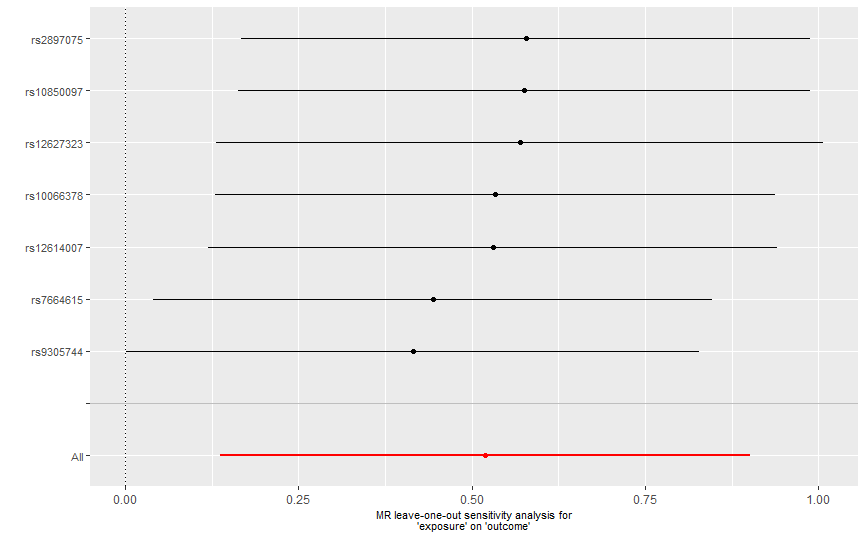


C

B

A

**Figure. S4** Leave one out plot. The causal effect of COVID-19 infection (A), COVID-19 hospitalization (B), COVID-19 severity(C) on focal epilepsy (ILAE).


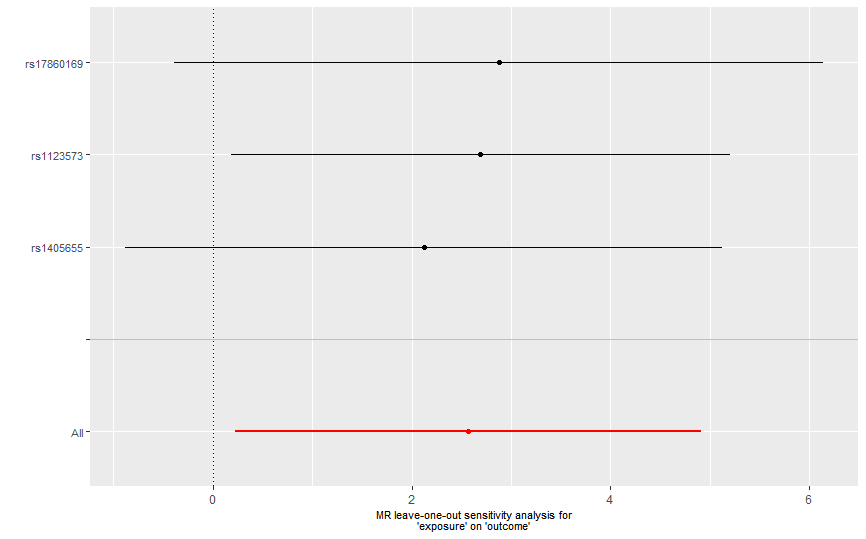

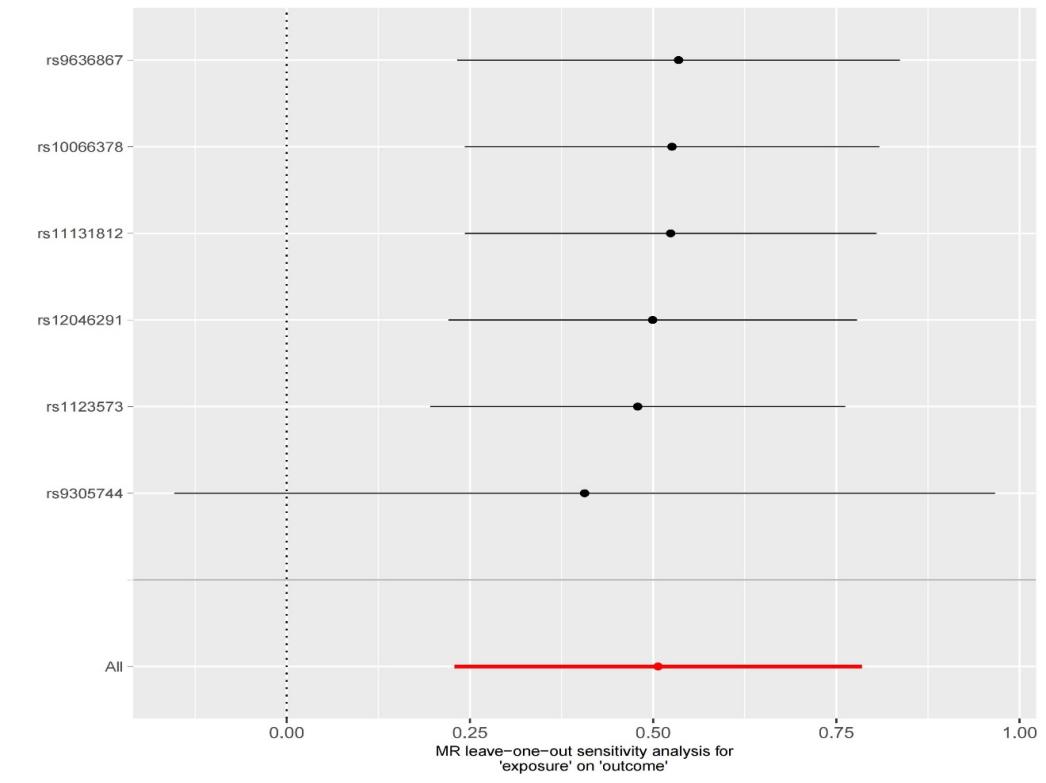

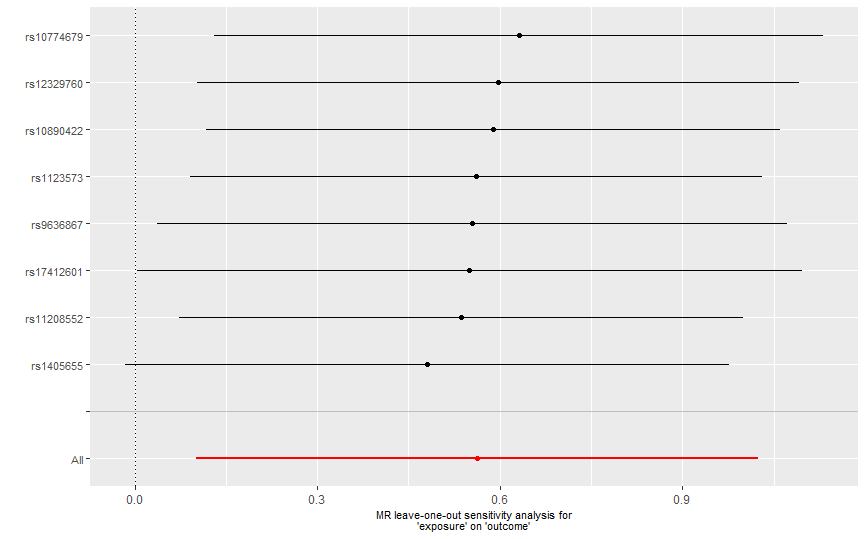


C

B

A

**Figure. S5** Leave one out plot. The causal effect of COVID-19 infection (A), COVID-19 hospitalization (B), COVID-19 severity(C) on generalized epilepsy (FinnGen).


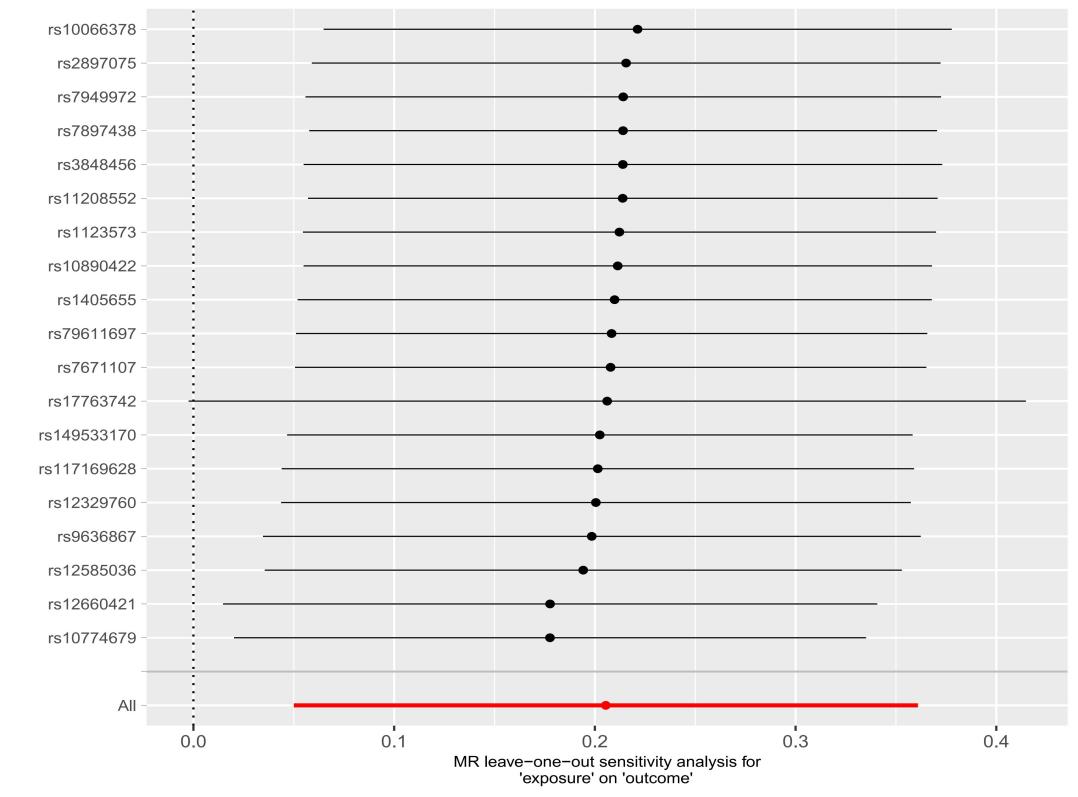

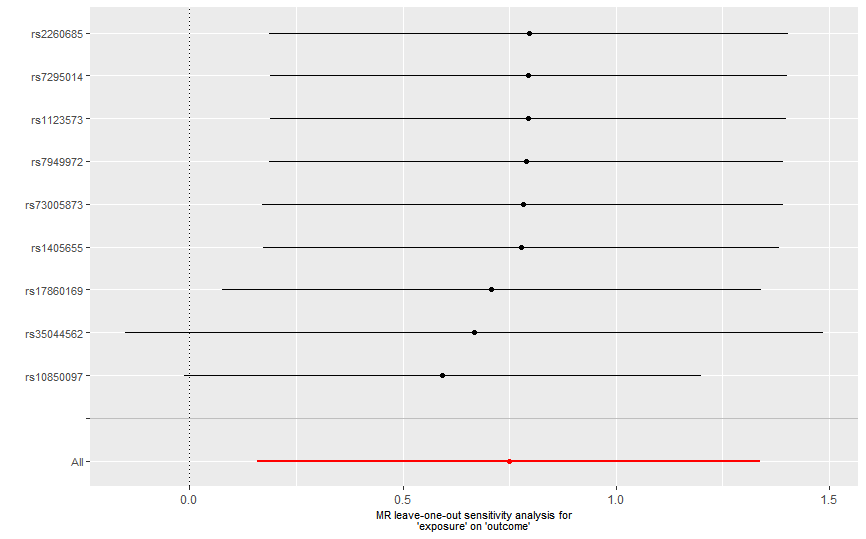

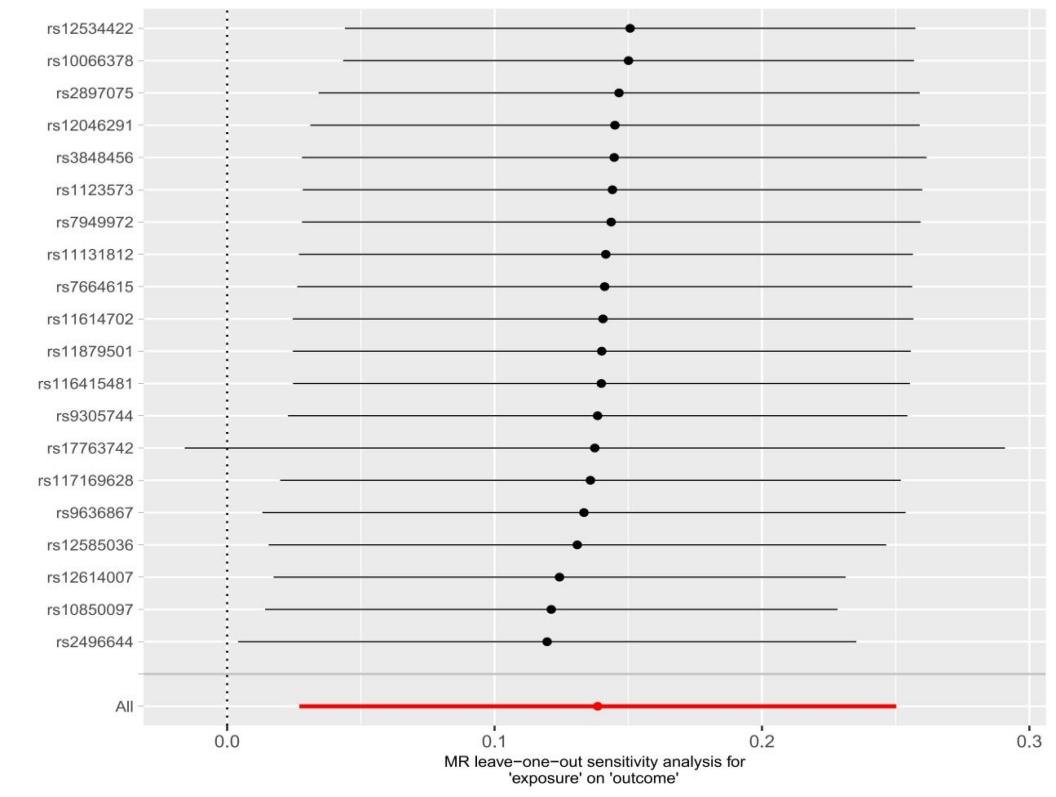


C

B

A

**Figure. S6** Leave one out plot. The causal effect of COVID-19 infection (A), COVID-19 hospitalization (B), COVID-19 severity(C) on generalized epilepsy (ILAE).


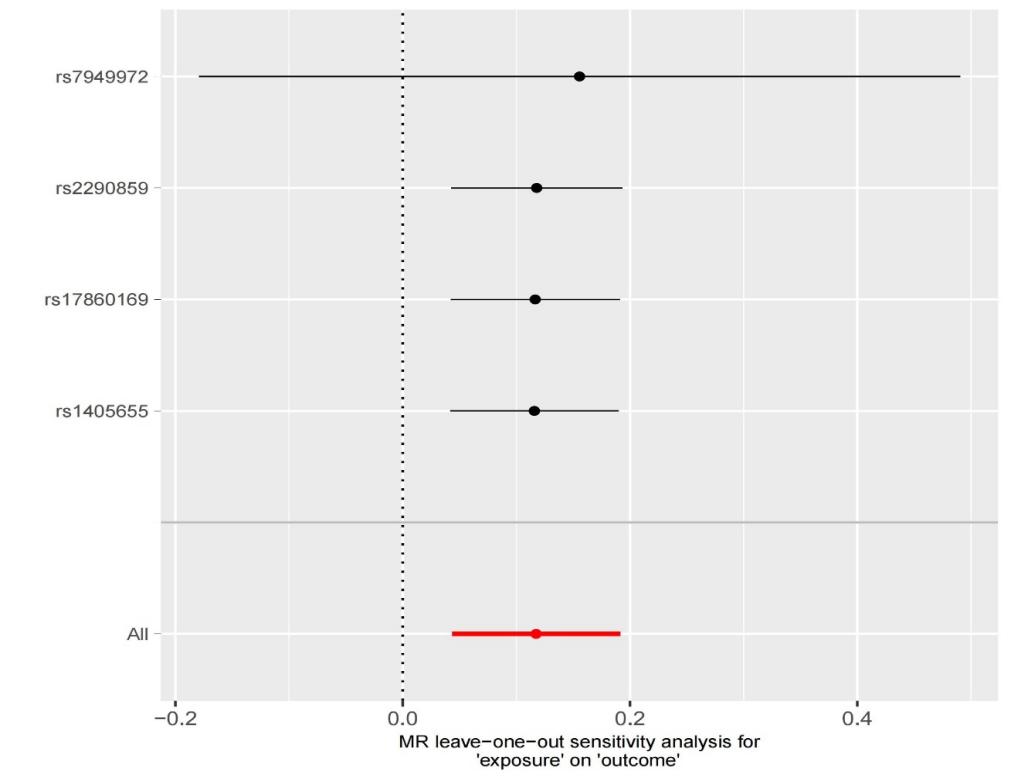

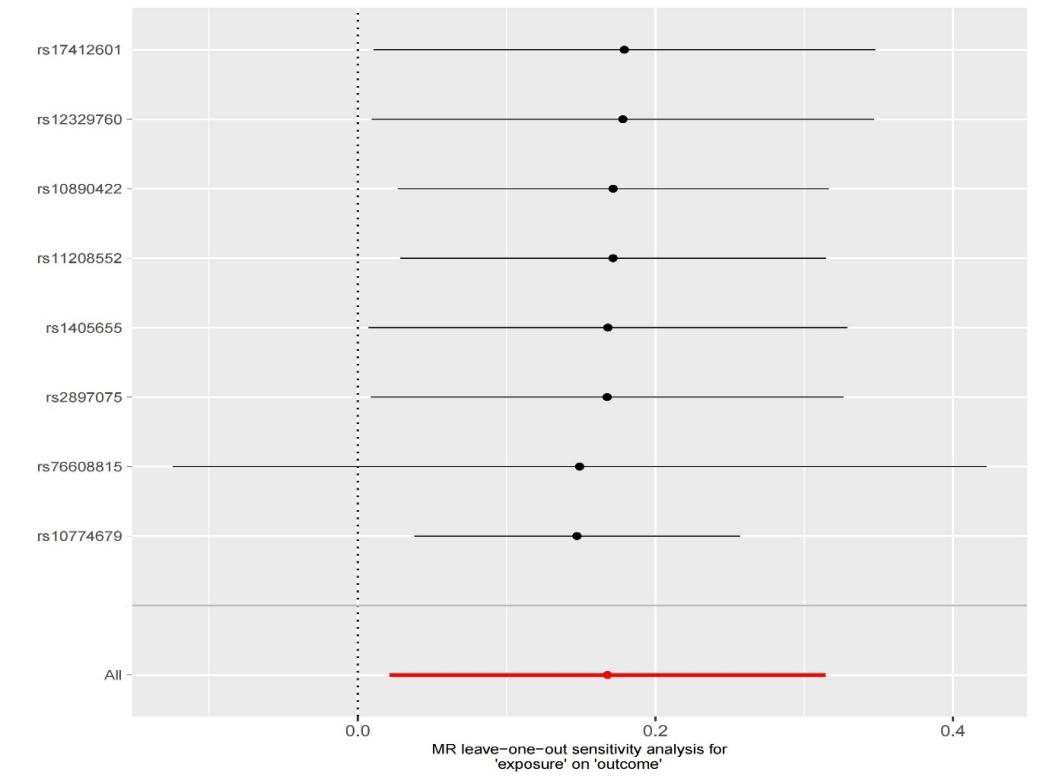

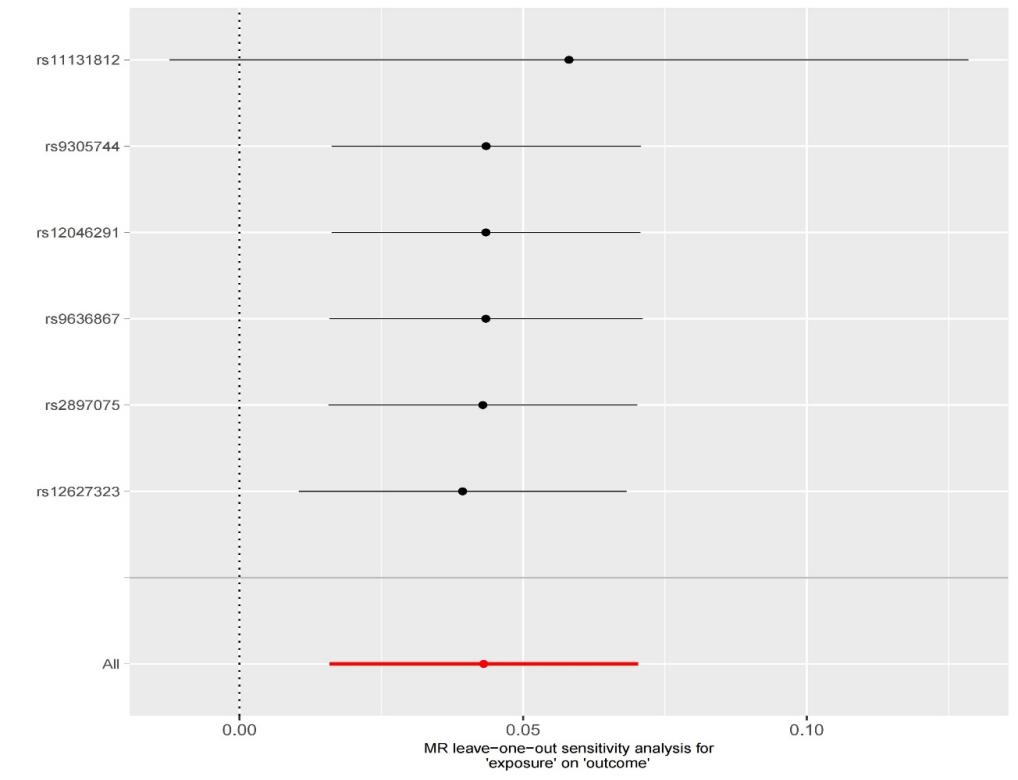


C

B

A

**Figure. S7** The causal effect of epilepsy (FinnGen) on COVID-19 (infection). (A) Scatter plot, (B) Funnel plot, (C) Forest plot, and (D) Leave one out plot.


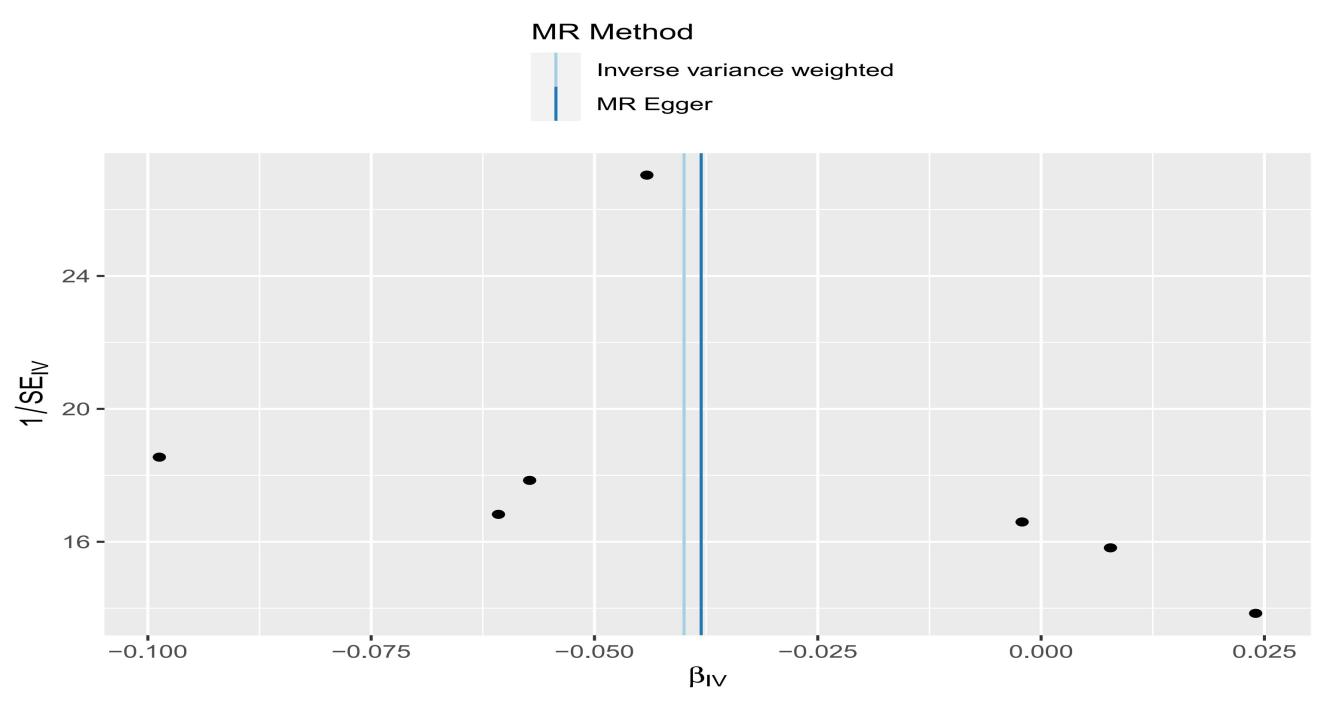

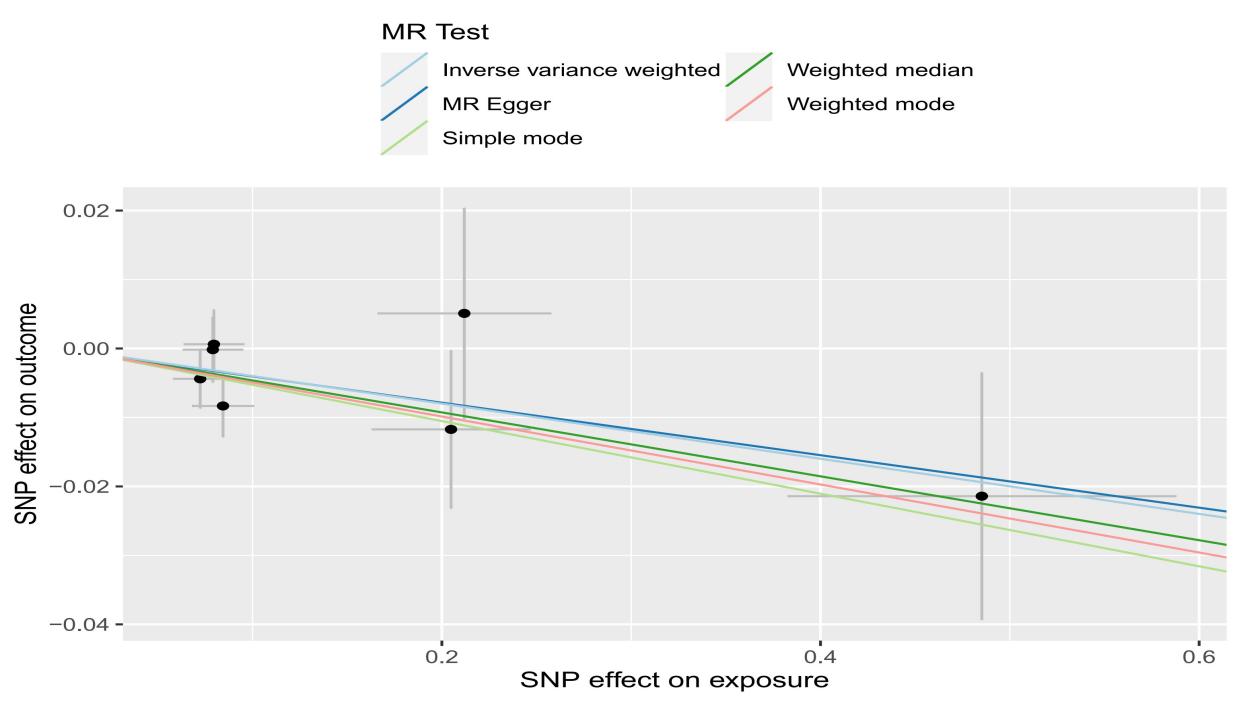

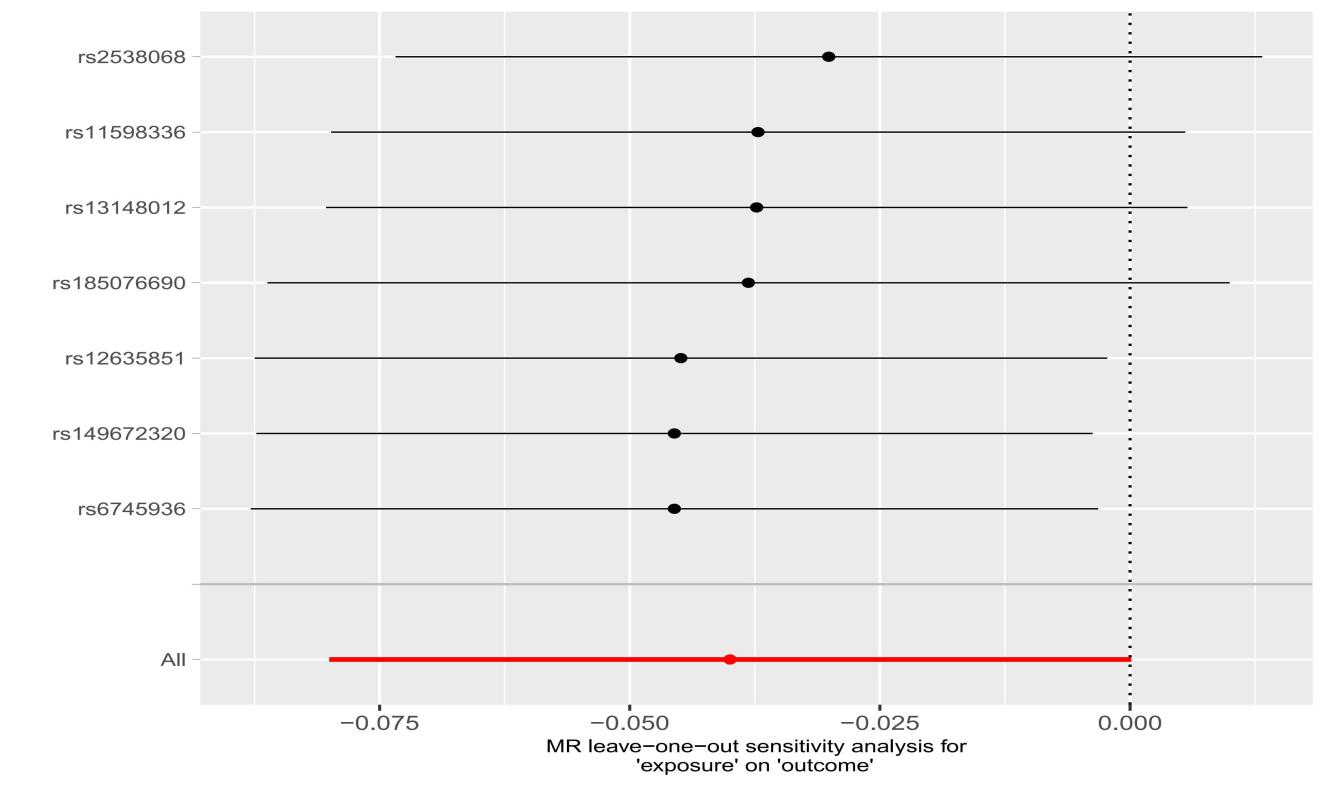

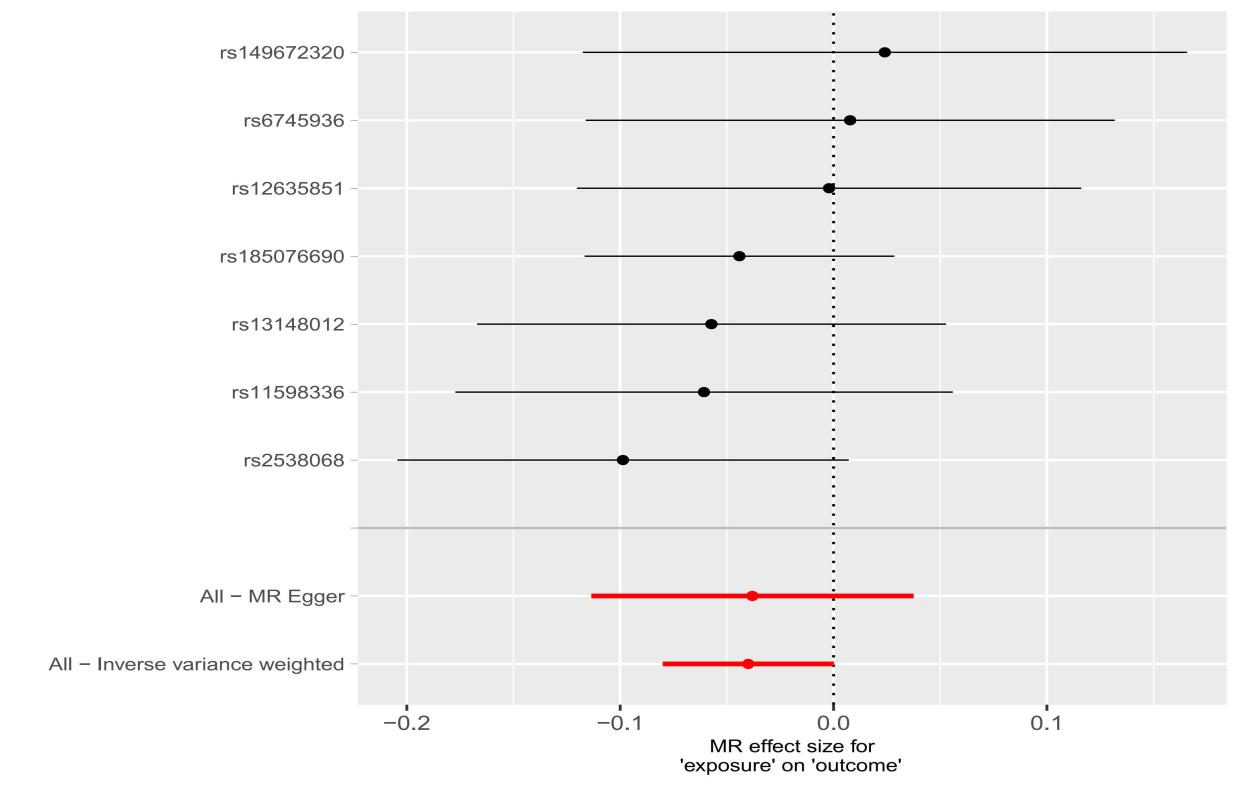


D

C

A

B

**Figure. S8** The causal effect of epilepsy (FinnGen) on COVID-19 (hospitalization). (A) Scatter plot, (B) Funnel plot, (C) Forest plot, and (D) Leave one out plot.


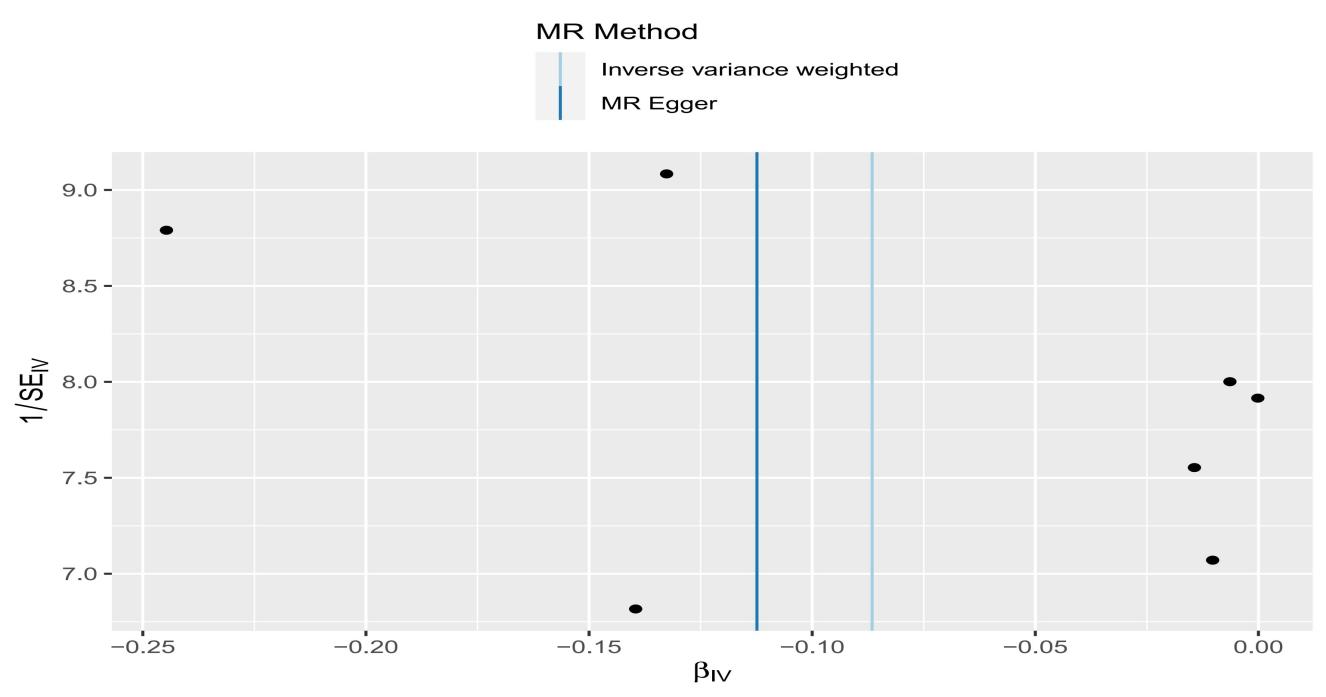

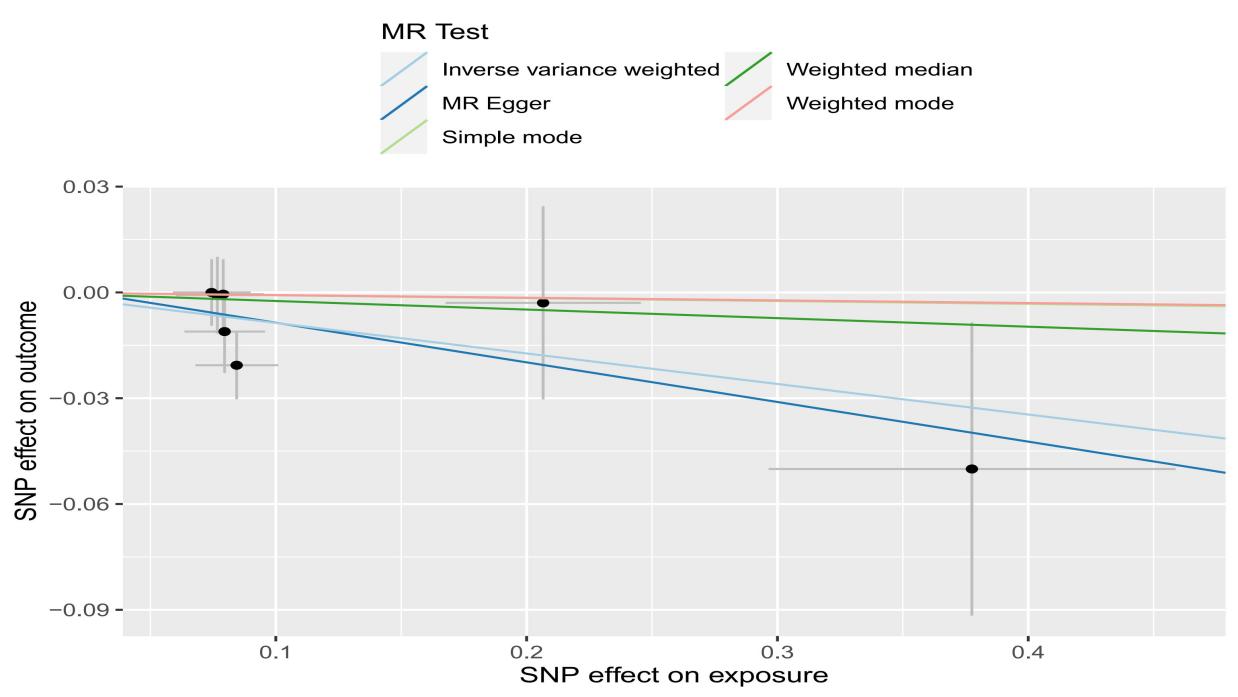

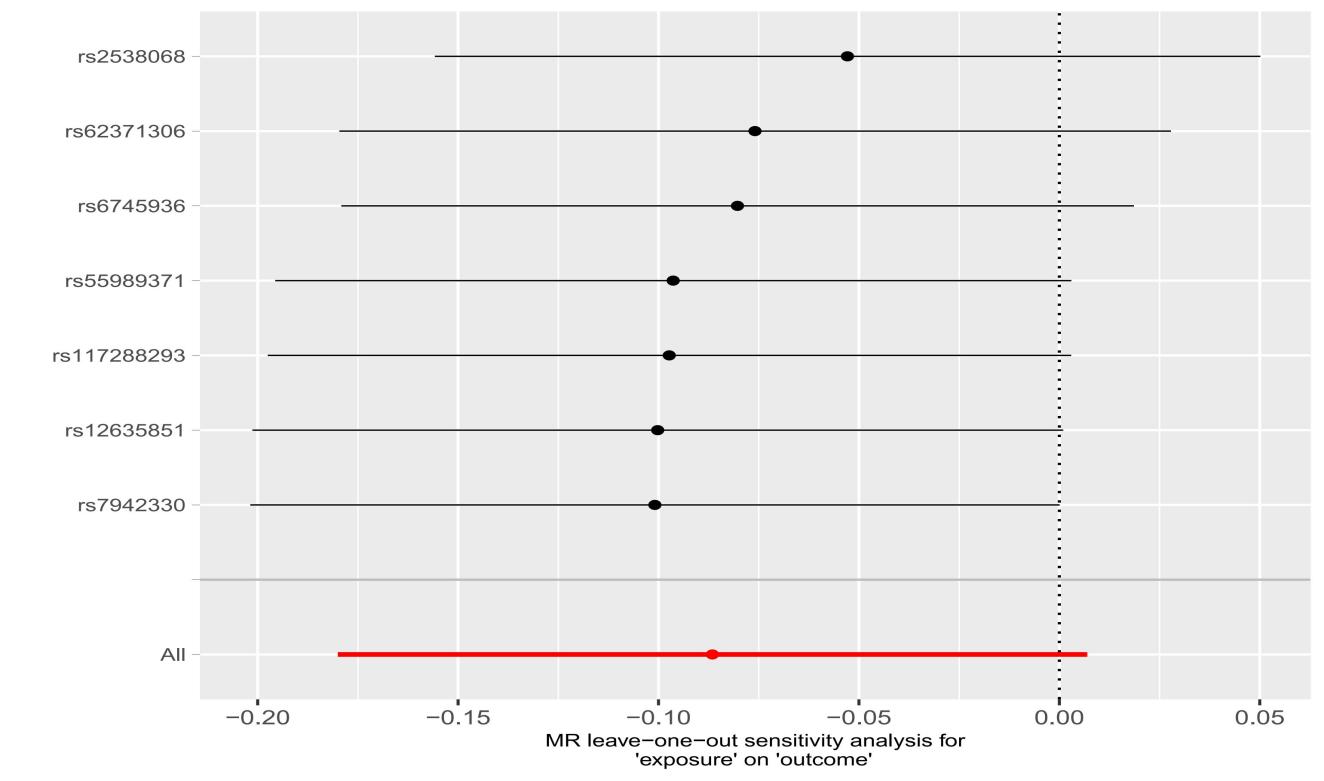

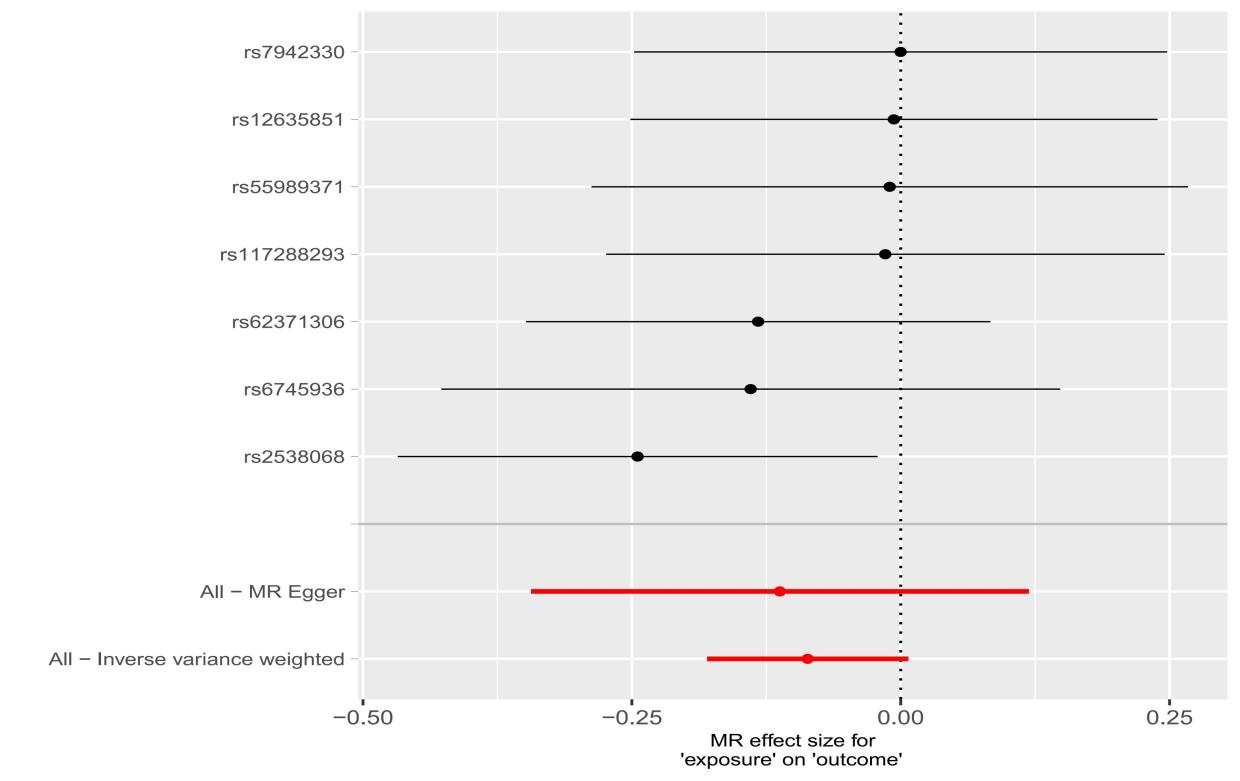


A

B

D

C

**Figure. S9** The causal effect of epilepsy (FinnGen) on COVID-19 (severity). (A) Scatter plot, (B) Funnel plot, (C) Forest plot, and (D) Leave one out plot.


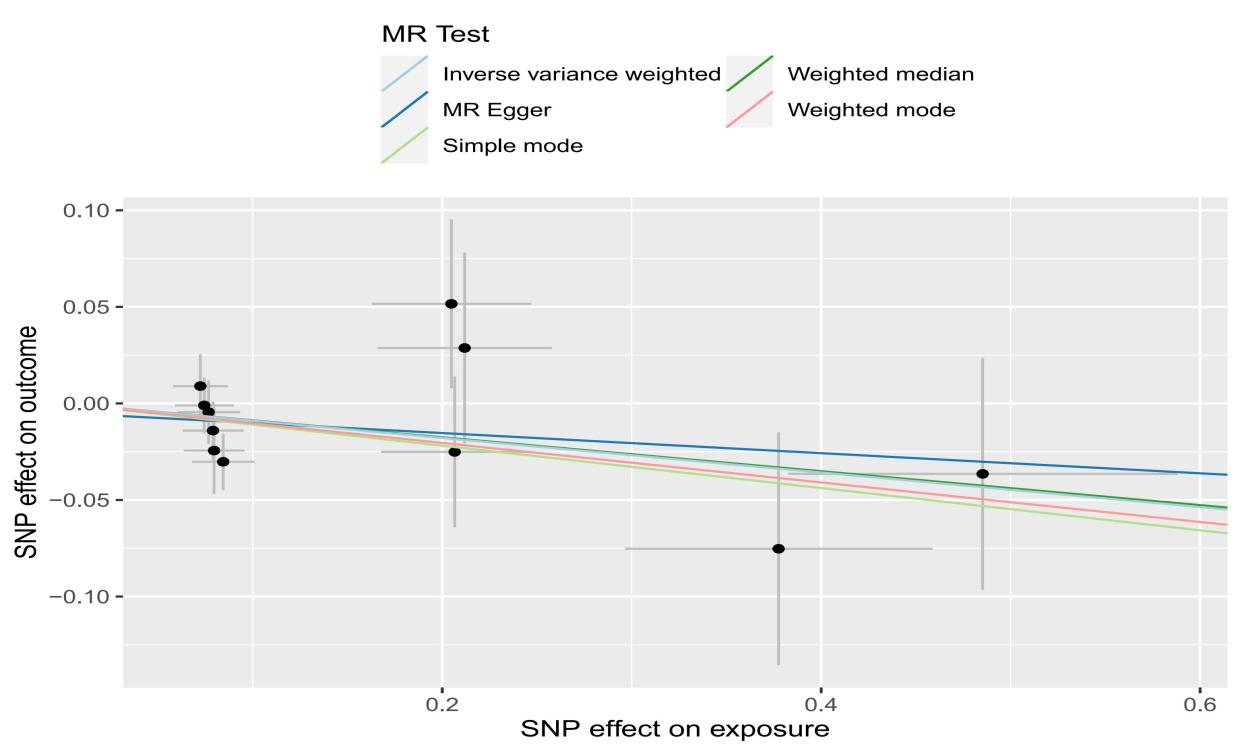

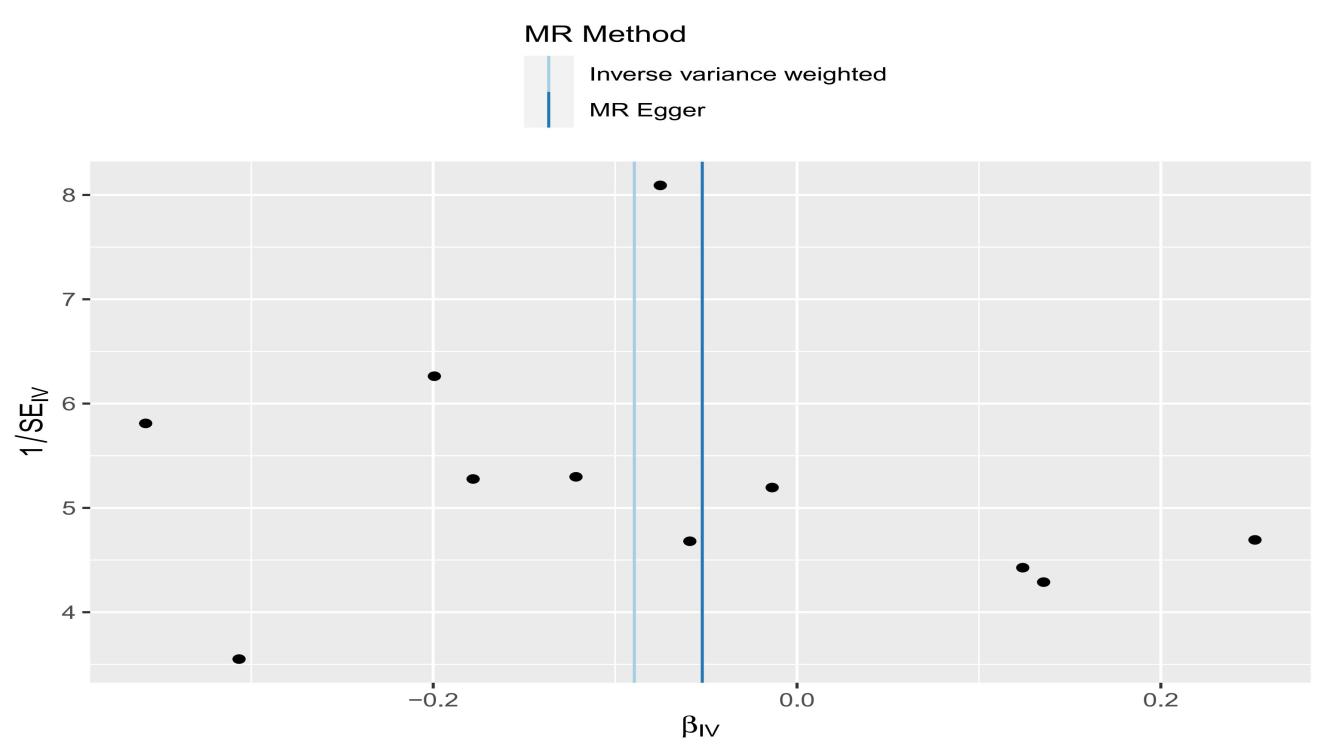

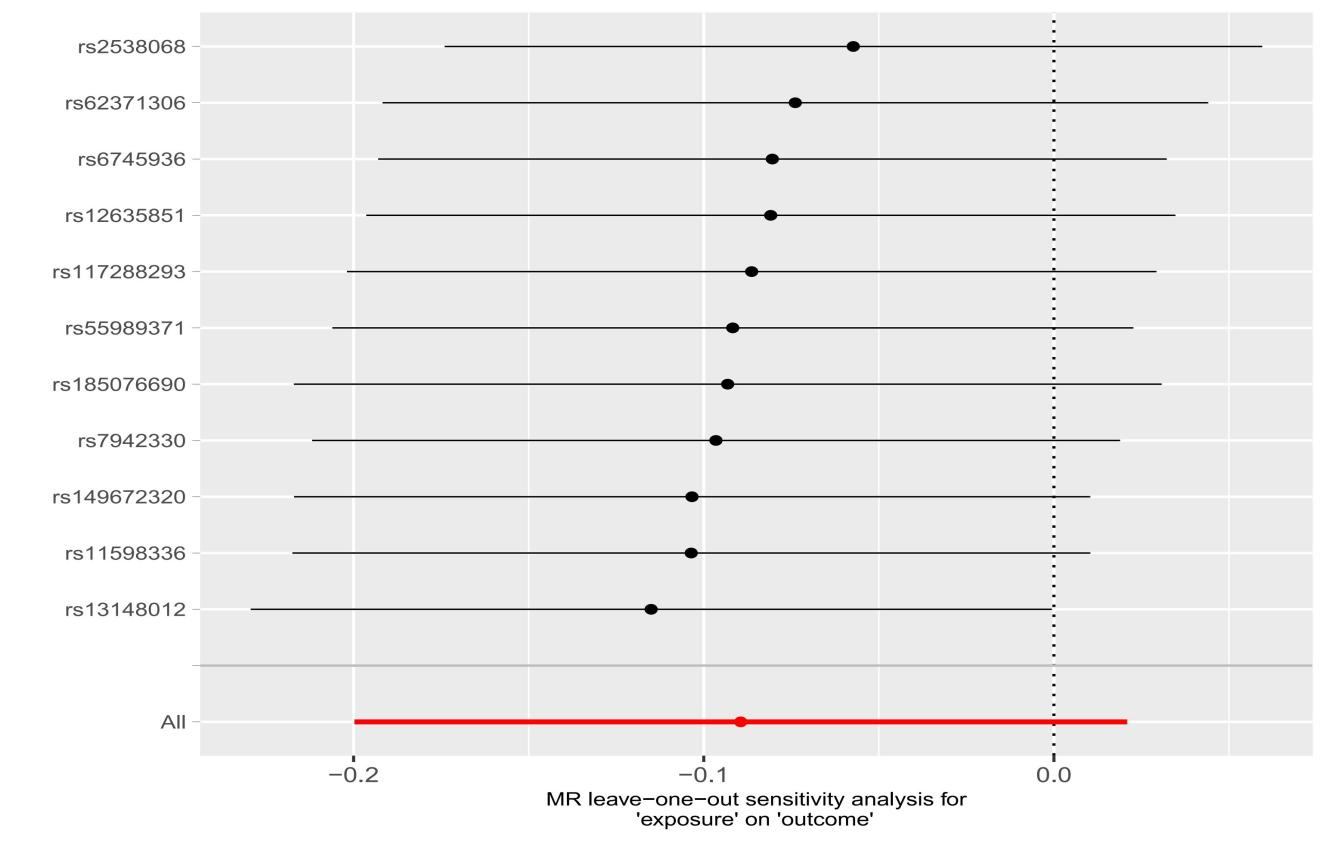

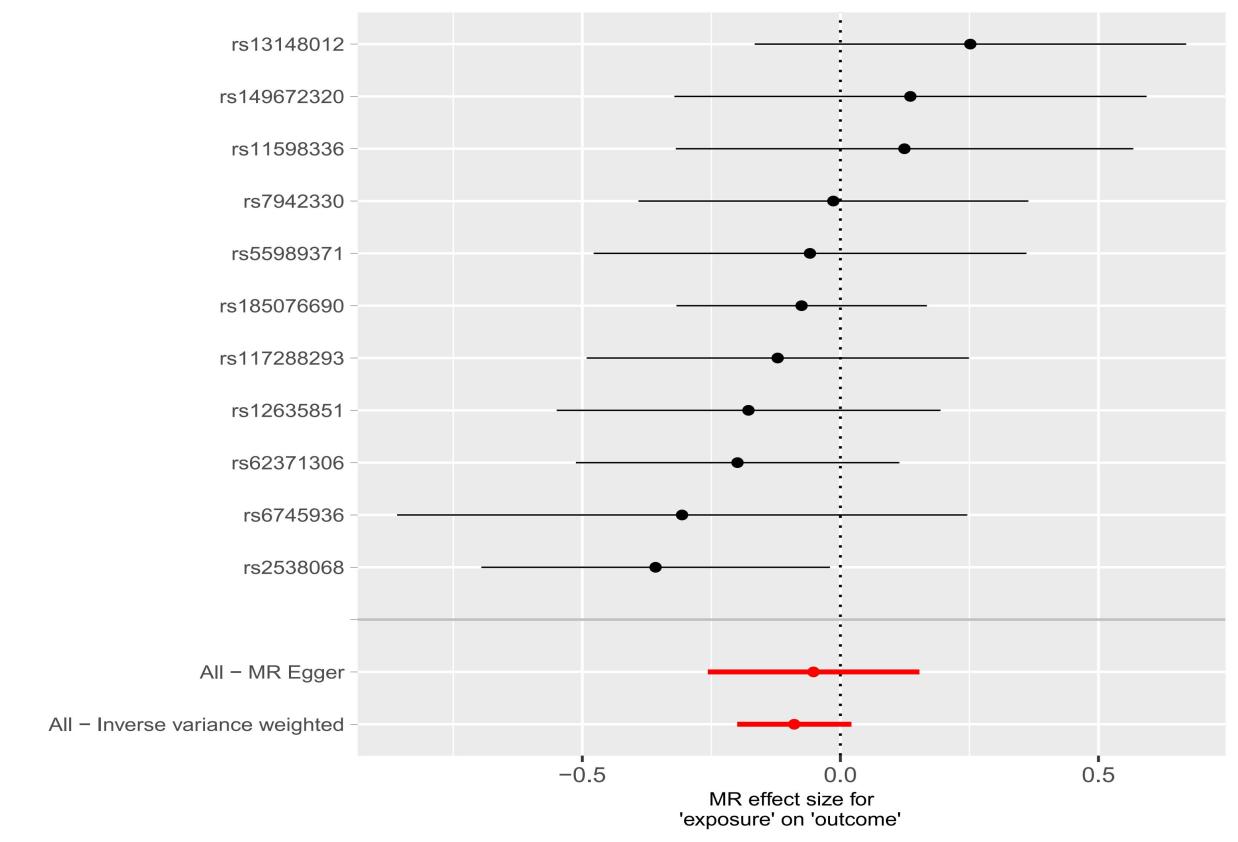


C

D

B

A

**Figure. S10** The causal effect of focal epilepsy (FinnGen) on COVID-19 (infection). (A) Scatter plot, (B) Funnel plot, (C) Forest plot, and (D) Leave one out plot.


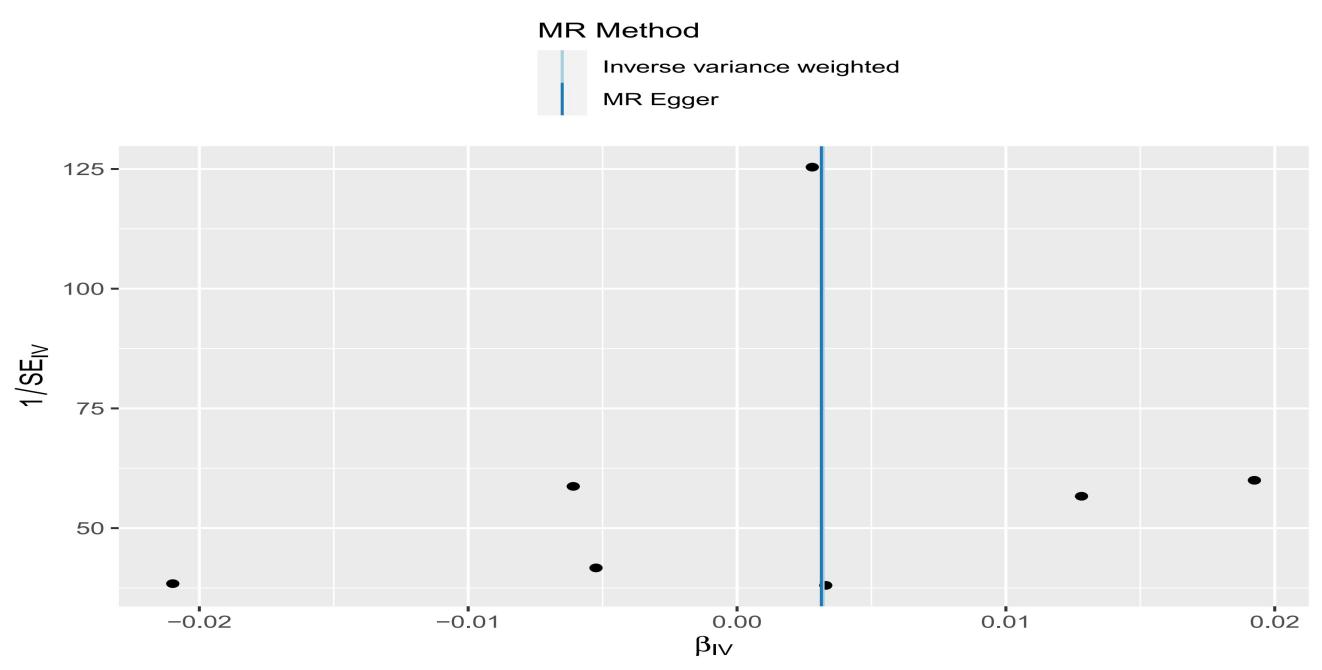

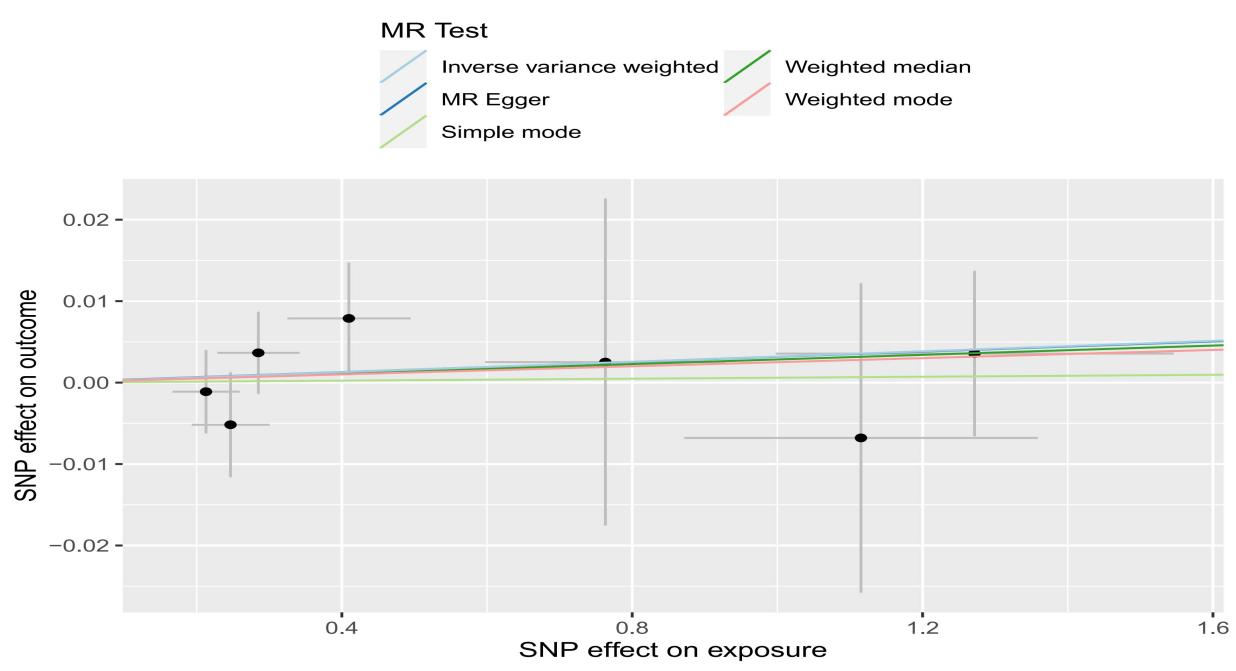

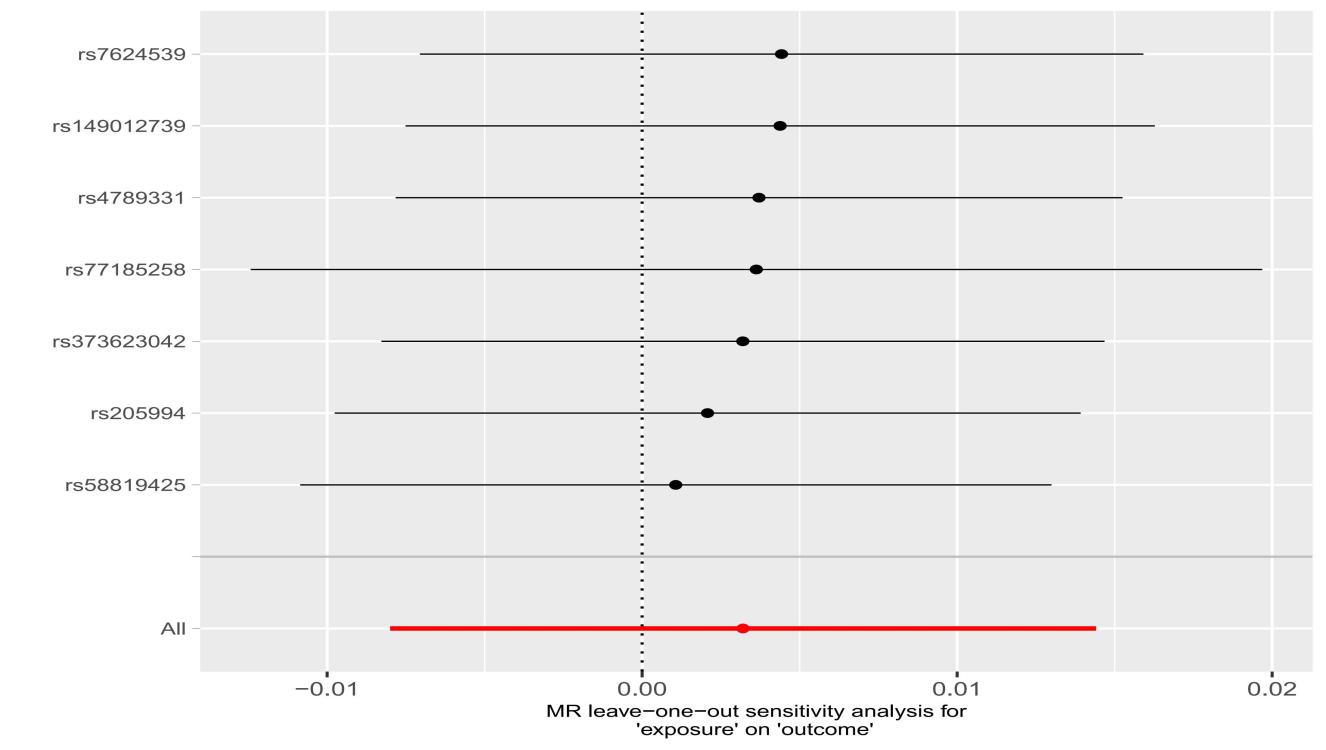

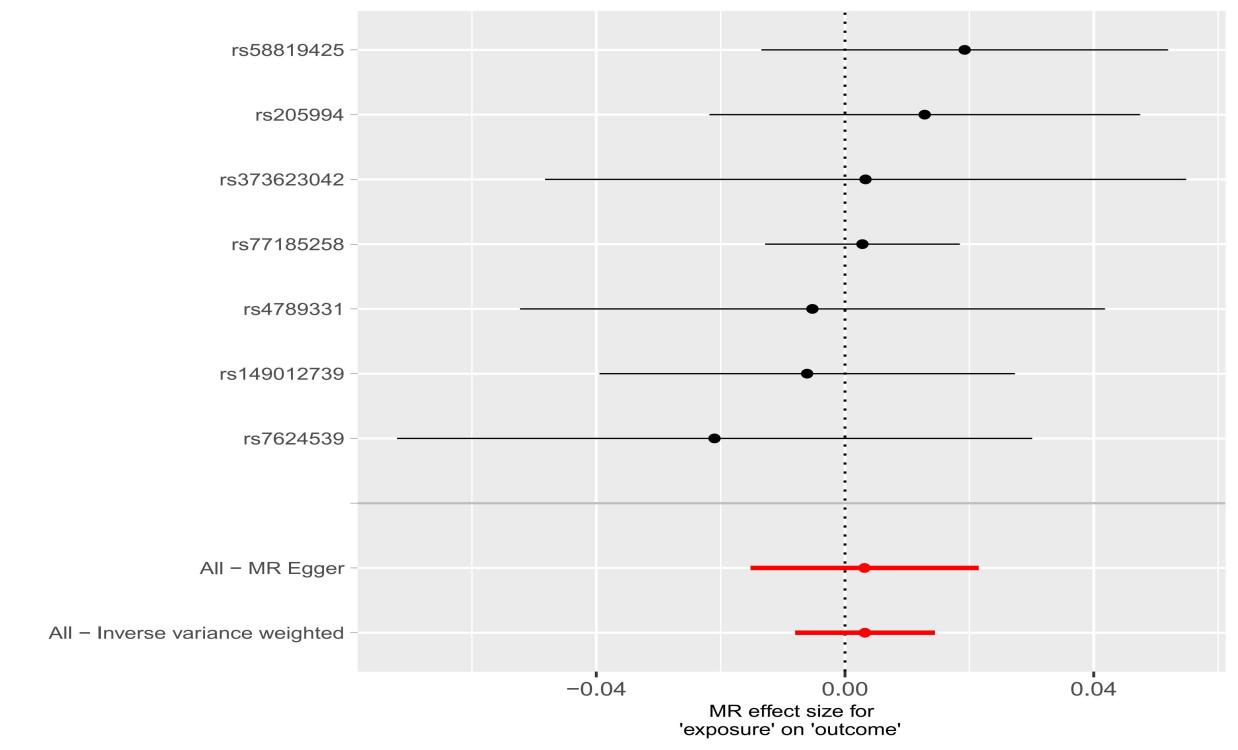


D

C

A

B

**Figure. S11** The causal effect of focal epilepsy (FinnGen) on COVID-19 (hospitalization). (A) Scatter plot, (B) Funnel plot, (C) Forest plot, and (D) Leave one out plot.


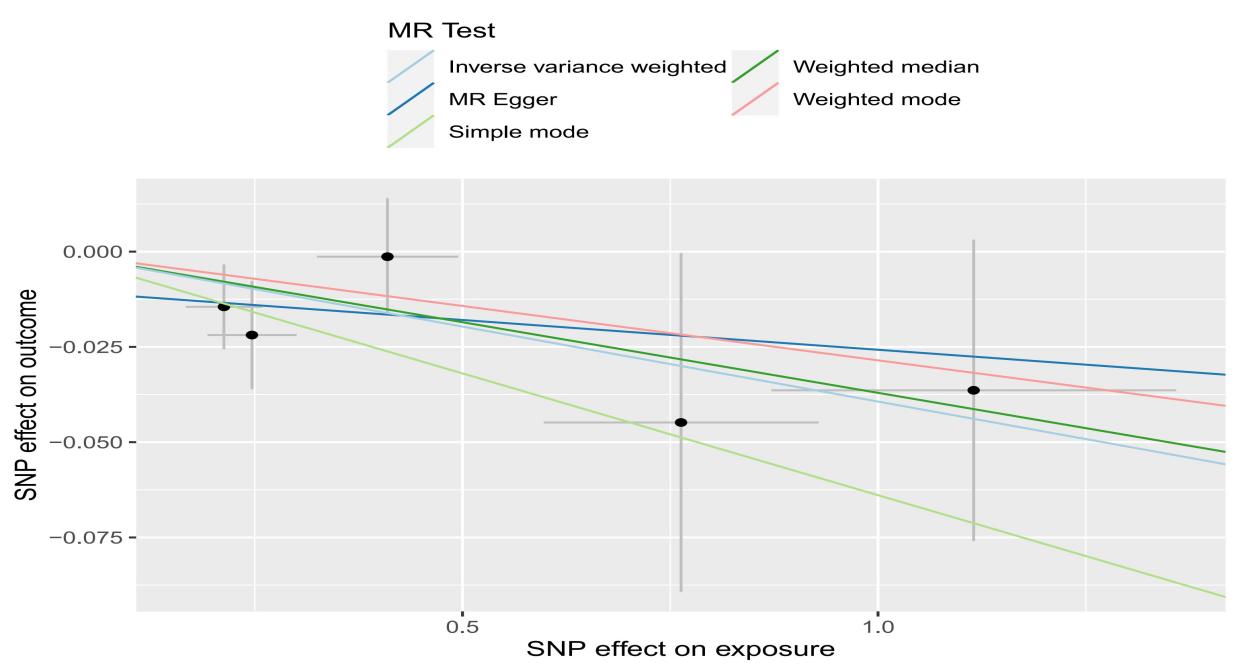

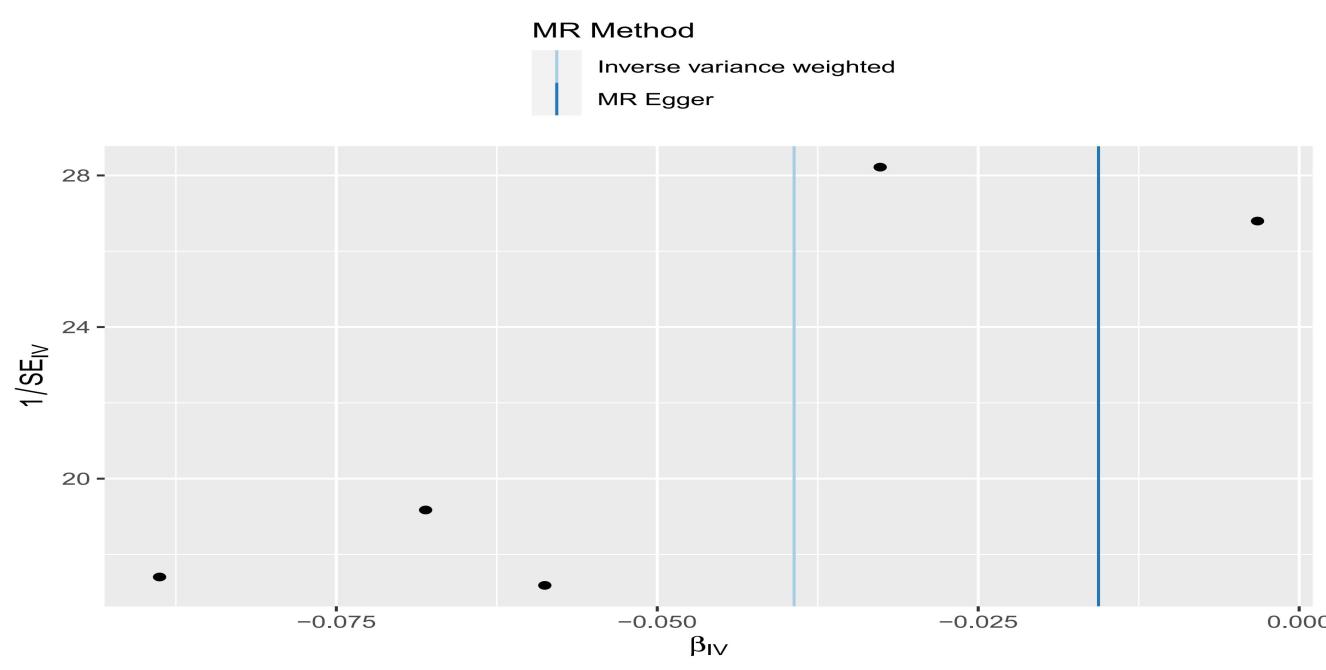

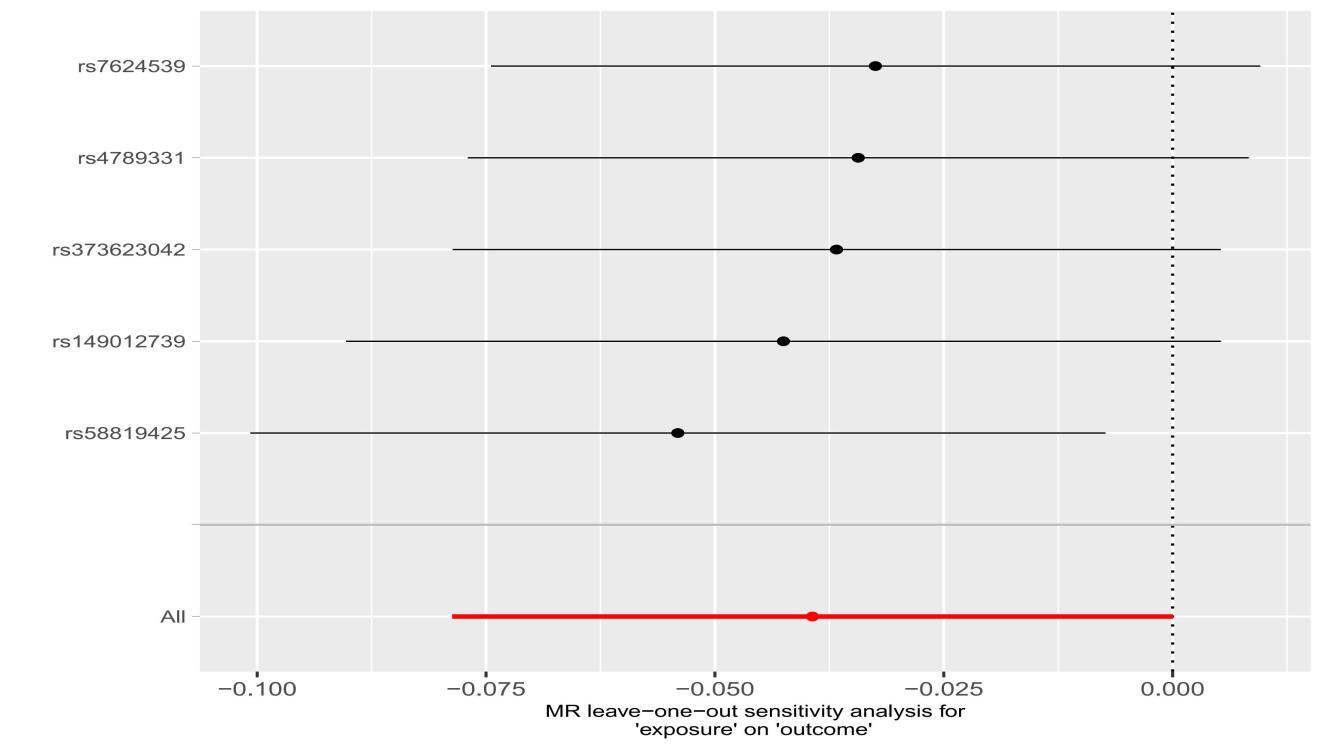

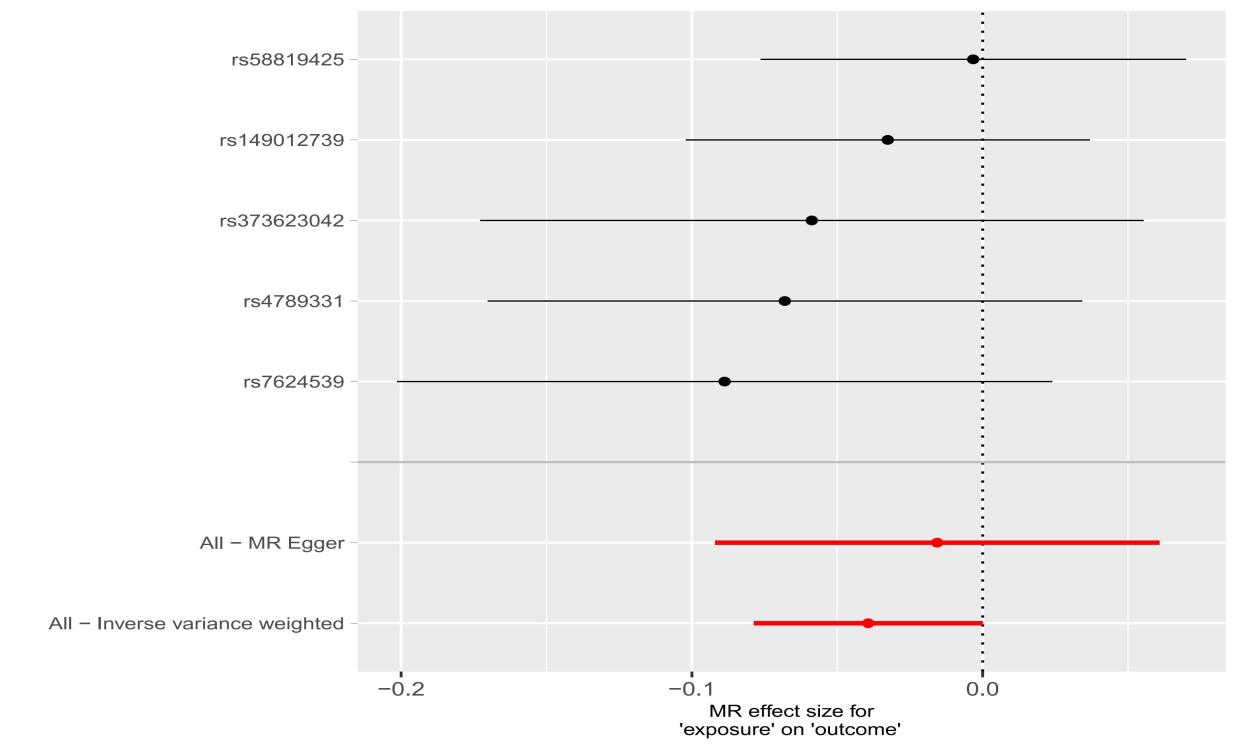


C

D

B

A

**Figure. S12** The causal effect of focal epilepsy (FinnGen) on COVID-19 (severity). (A) Scatter plot, (B) Funnel plot, (C) Forest plot, and (D) Leave one out plot.


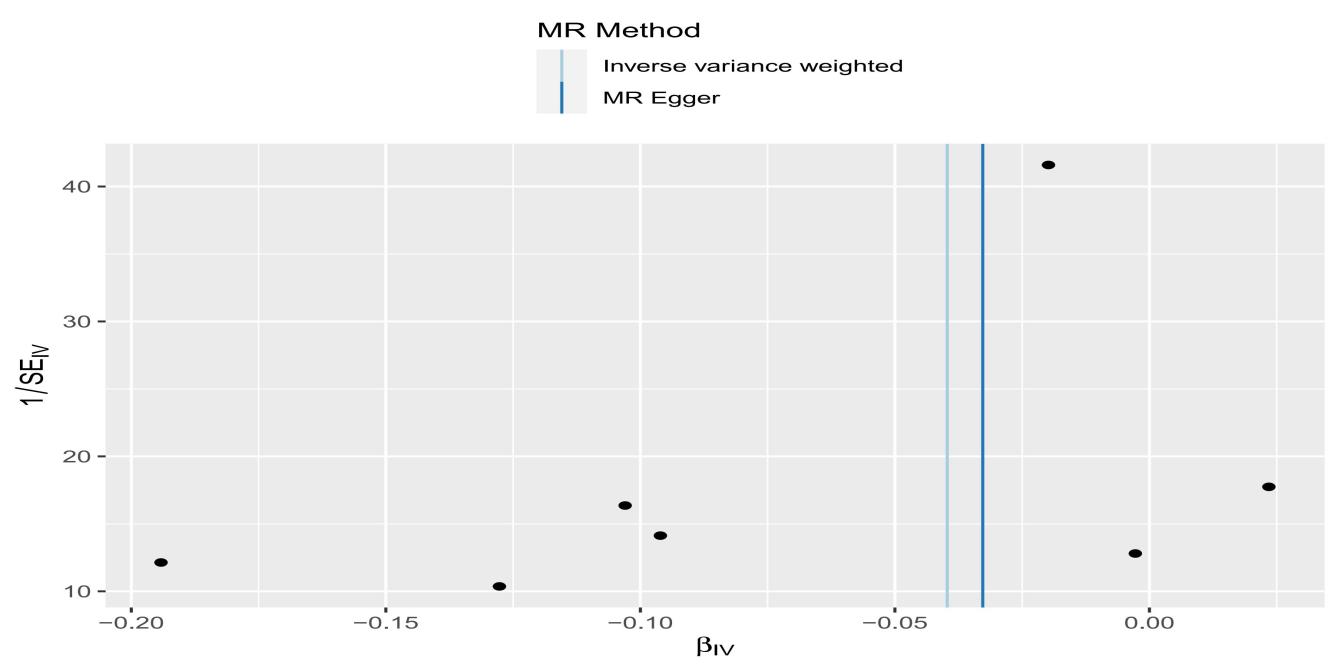

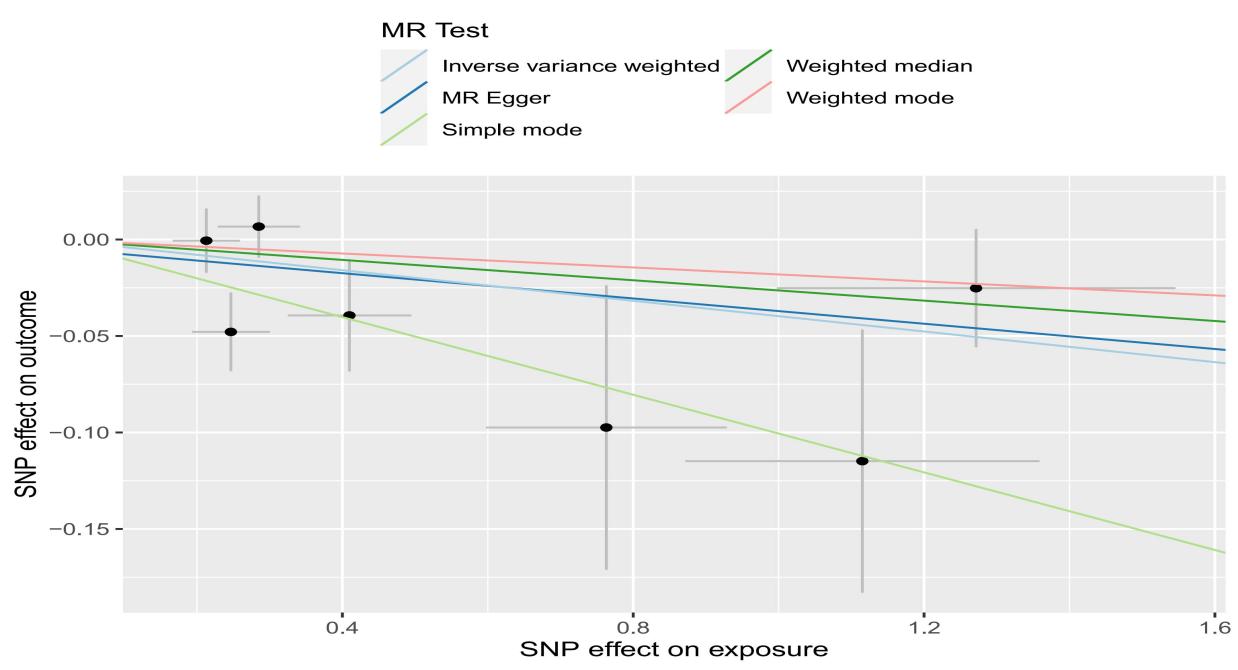

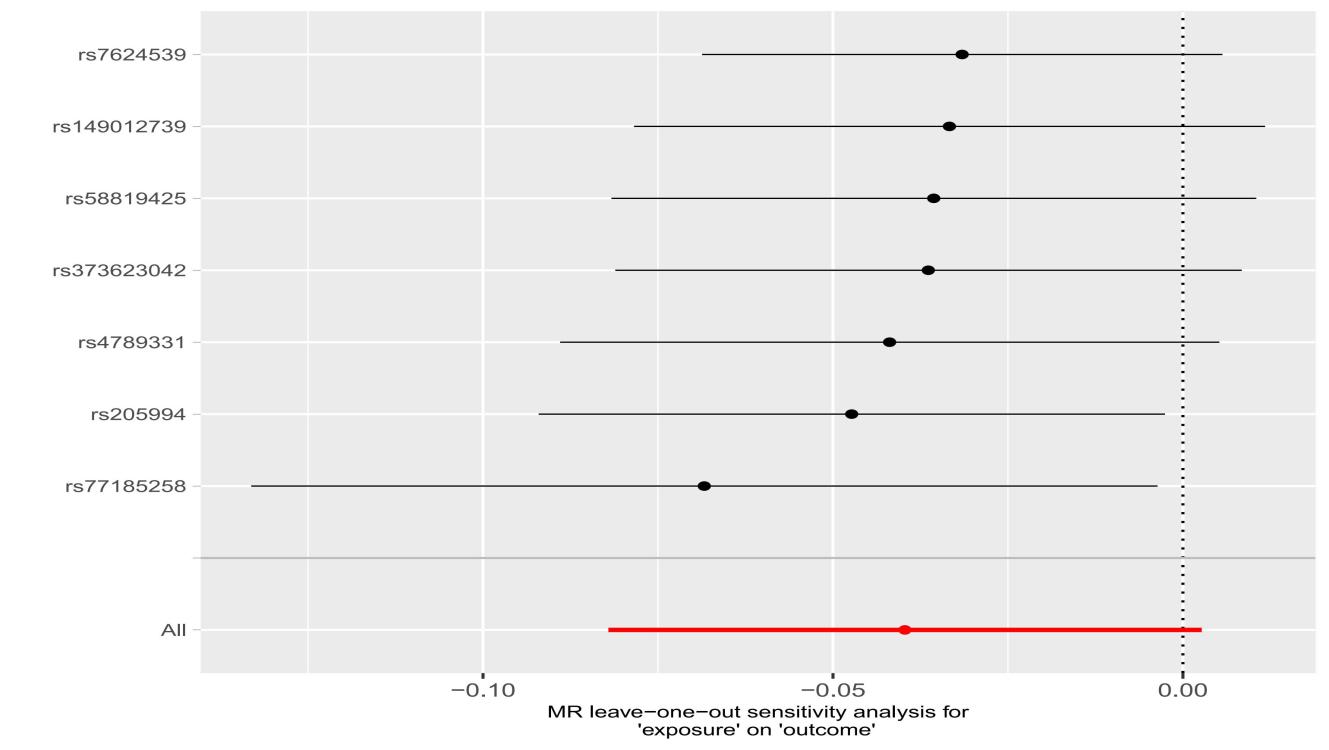

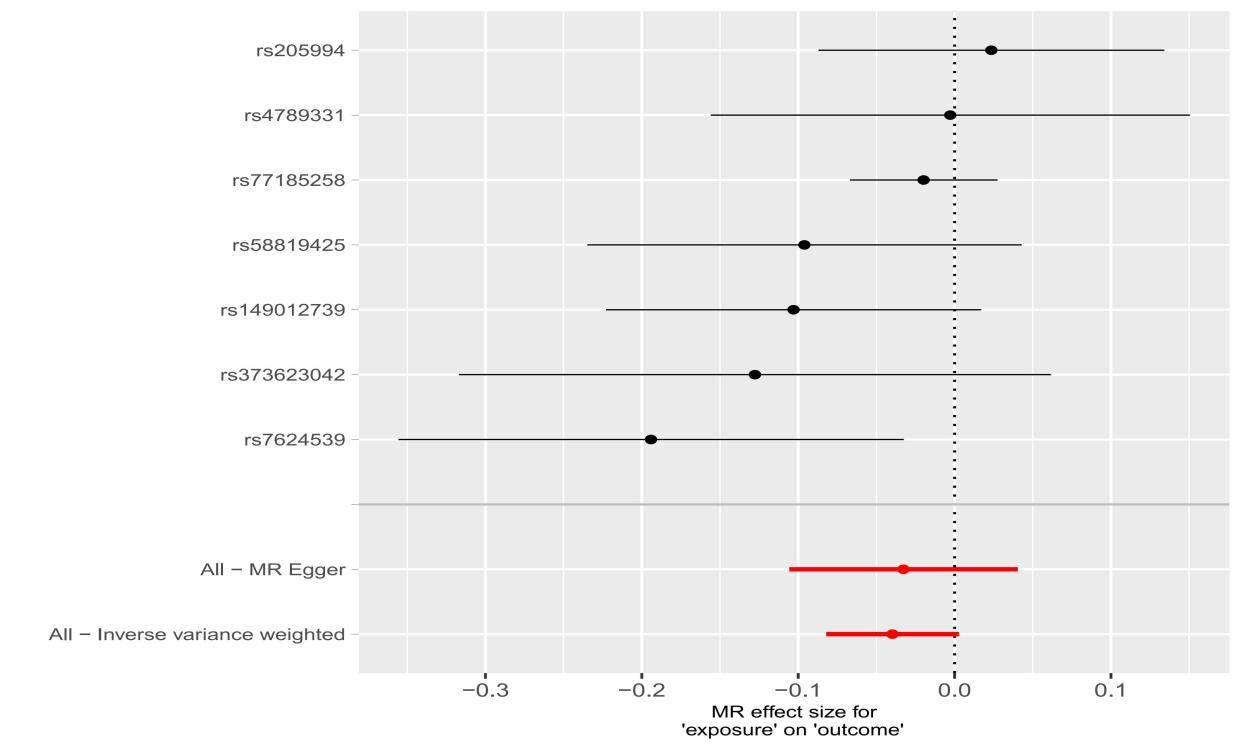


C

D

A

B

**Figure. S13** The causal effect of generalized epilepsy (FinnGen) on COVID-19 (infection). (A) Scatter plot, (B) Funnel plot, (C) Forest plot, and (D) Leave one out plot.


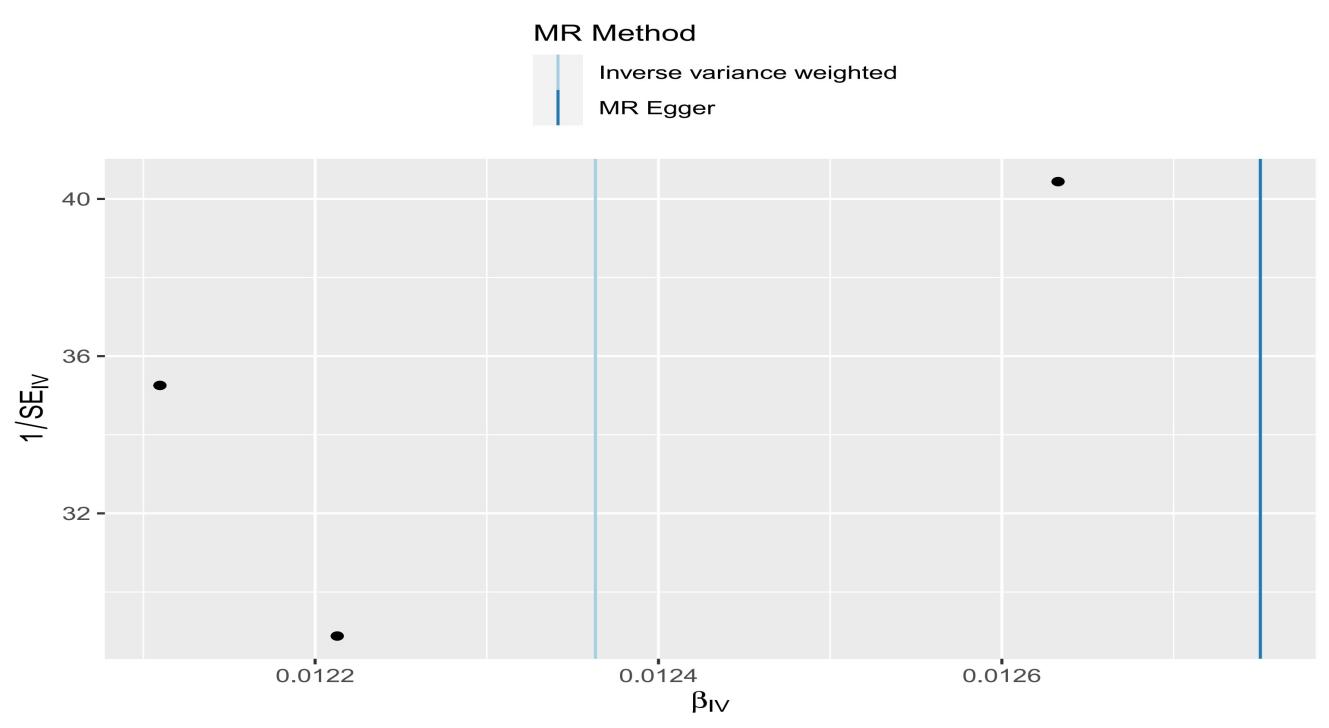

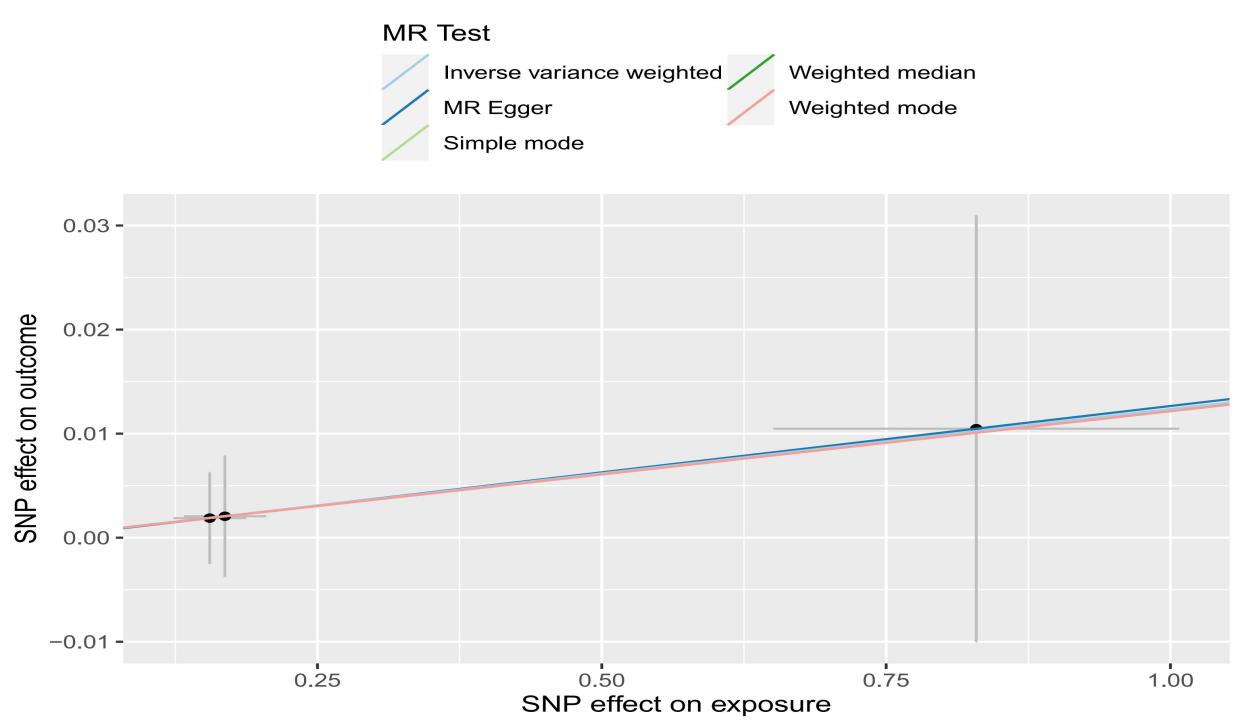

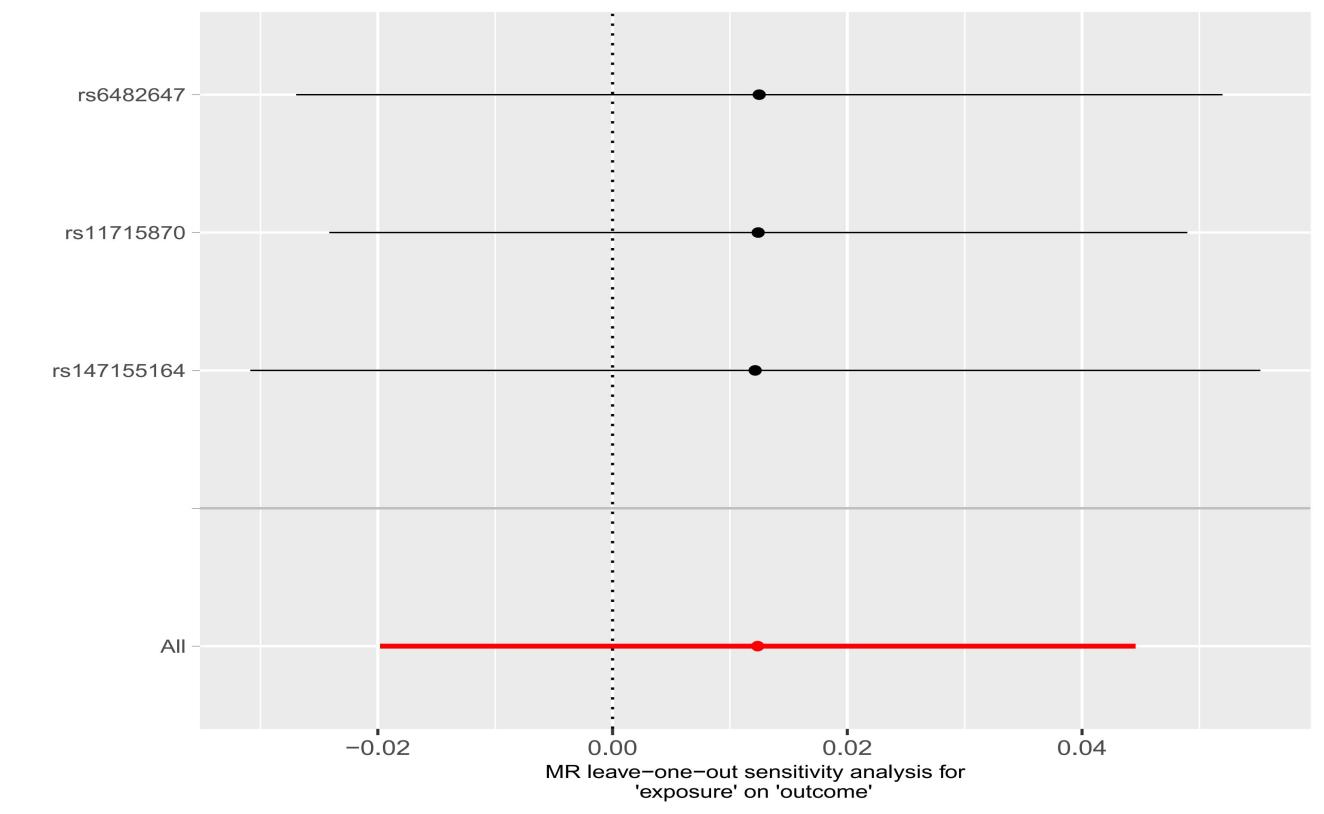

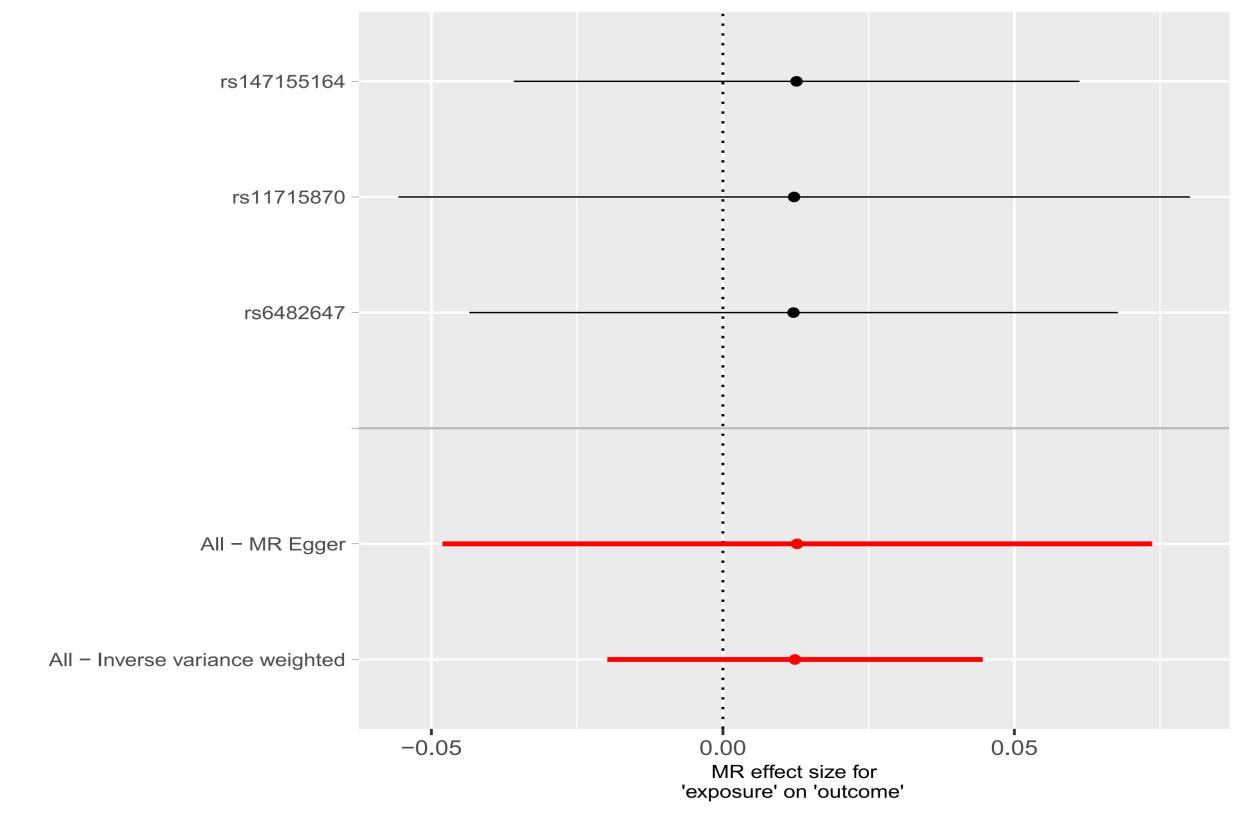


C

D

A

B

**Figure. S14** The causal effect of generalized epilepsy (FinnGen) on COVID-19 (hospitalization). (A) Scatter plot, (B) Funnel plot, (C) Forest plot, and (D) Leave one out plot.


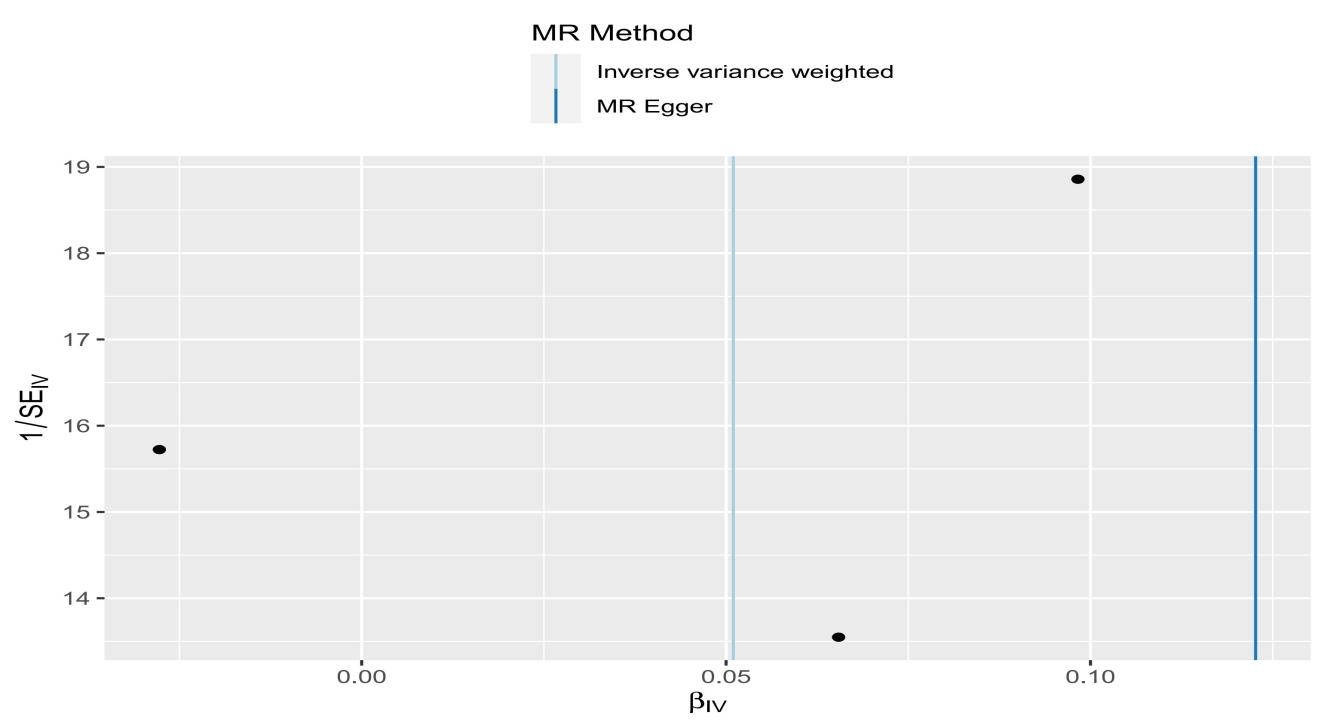

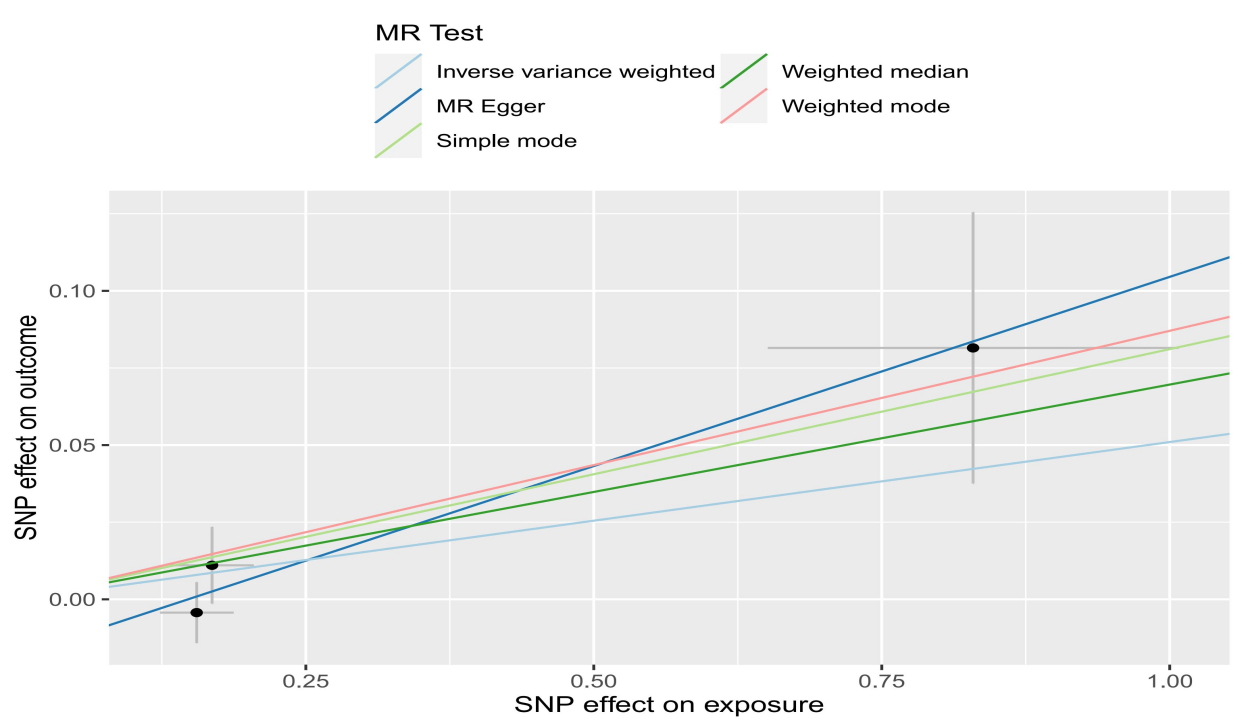

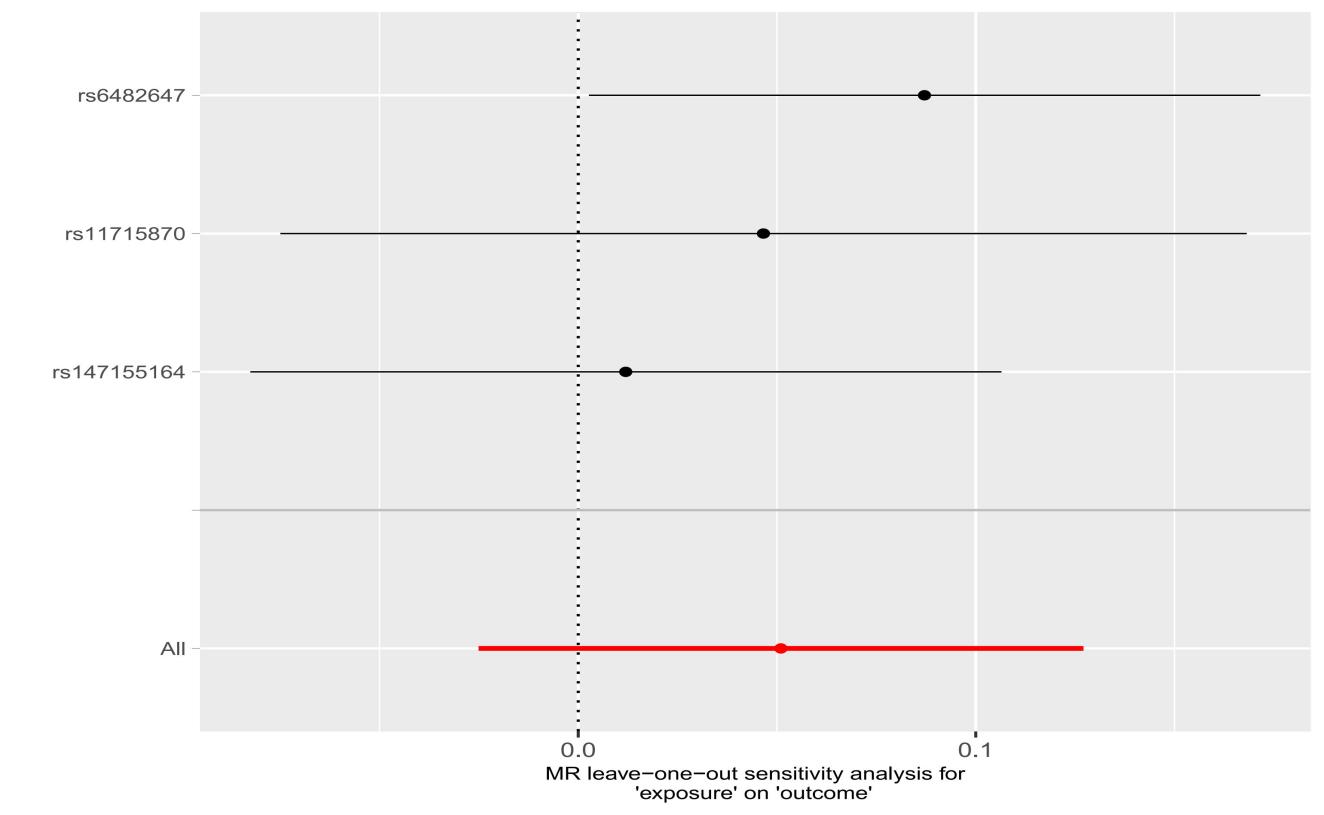

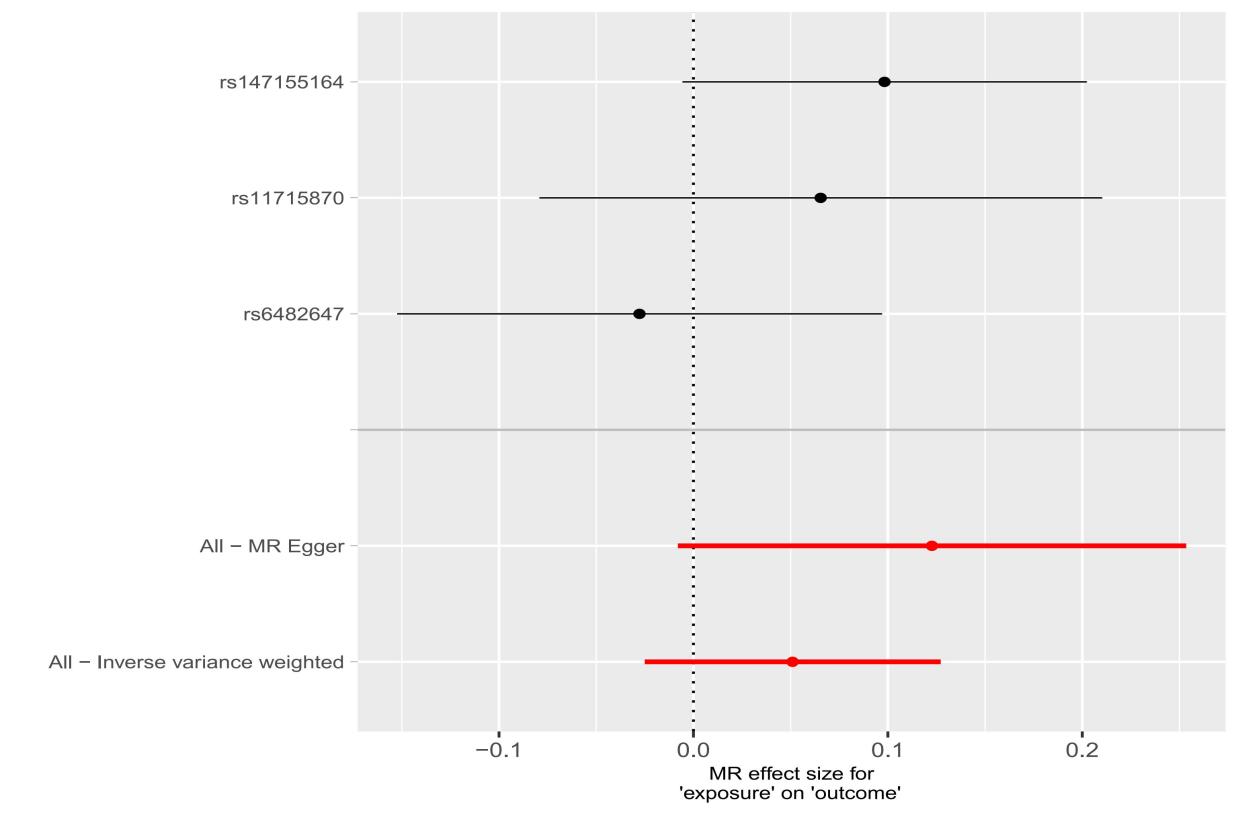


C

D

A

B

**Figure. S15** The causal effect of generalized epilepsy (FinnGen) on COVID-19 (severity). (A) Scatter plot, (B) Funnel plot, (C) Forest plot, and (D) Leave one out plot.


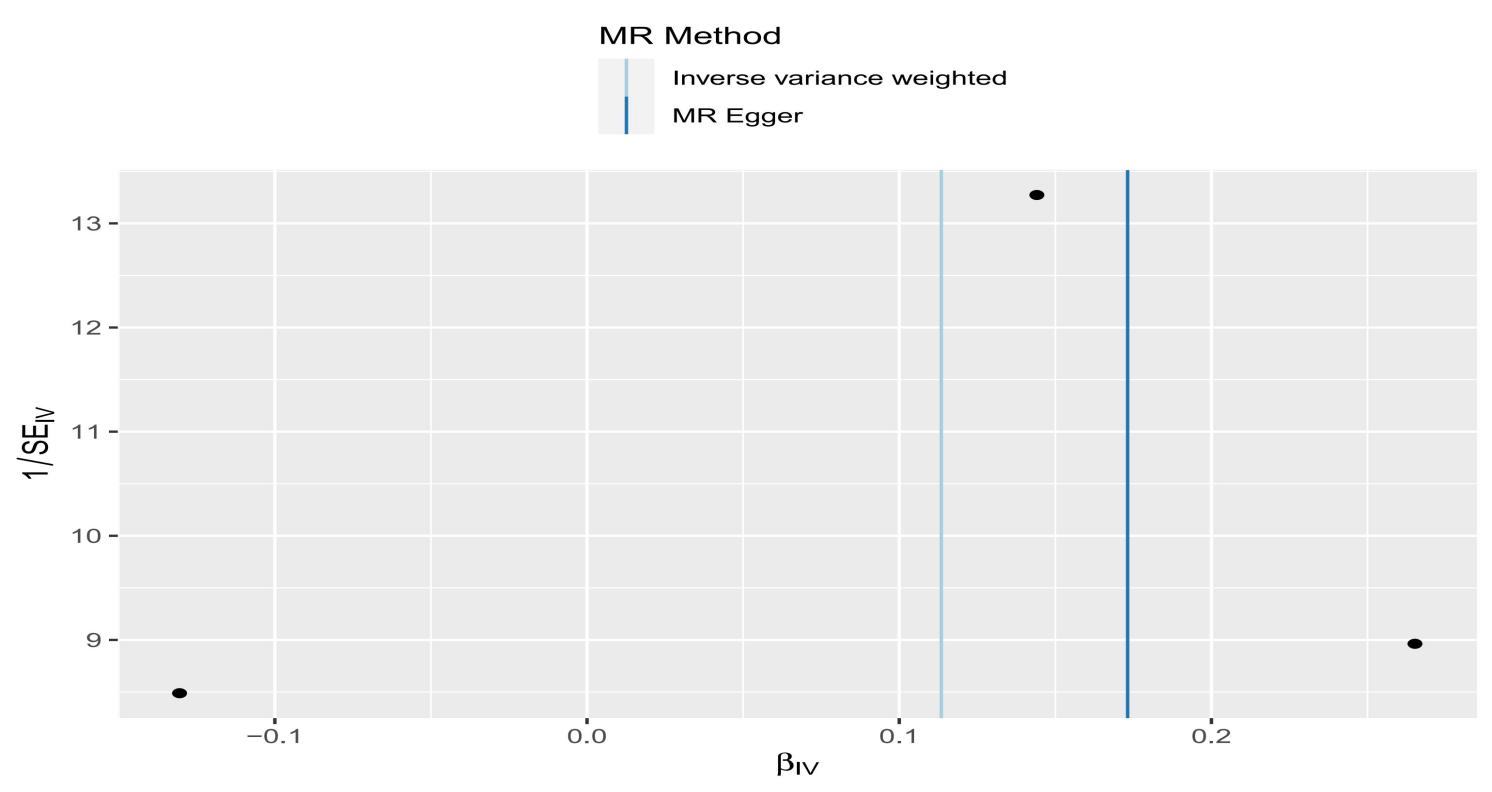

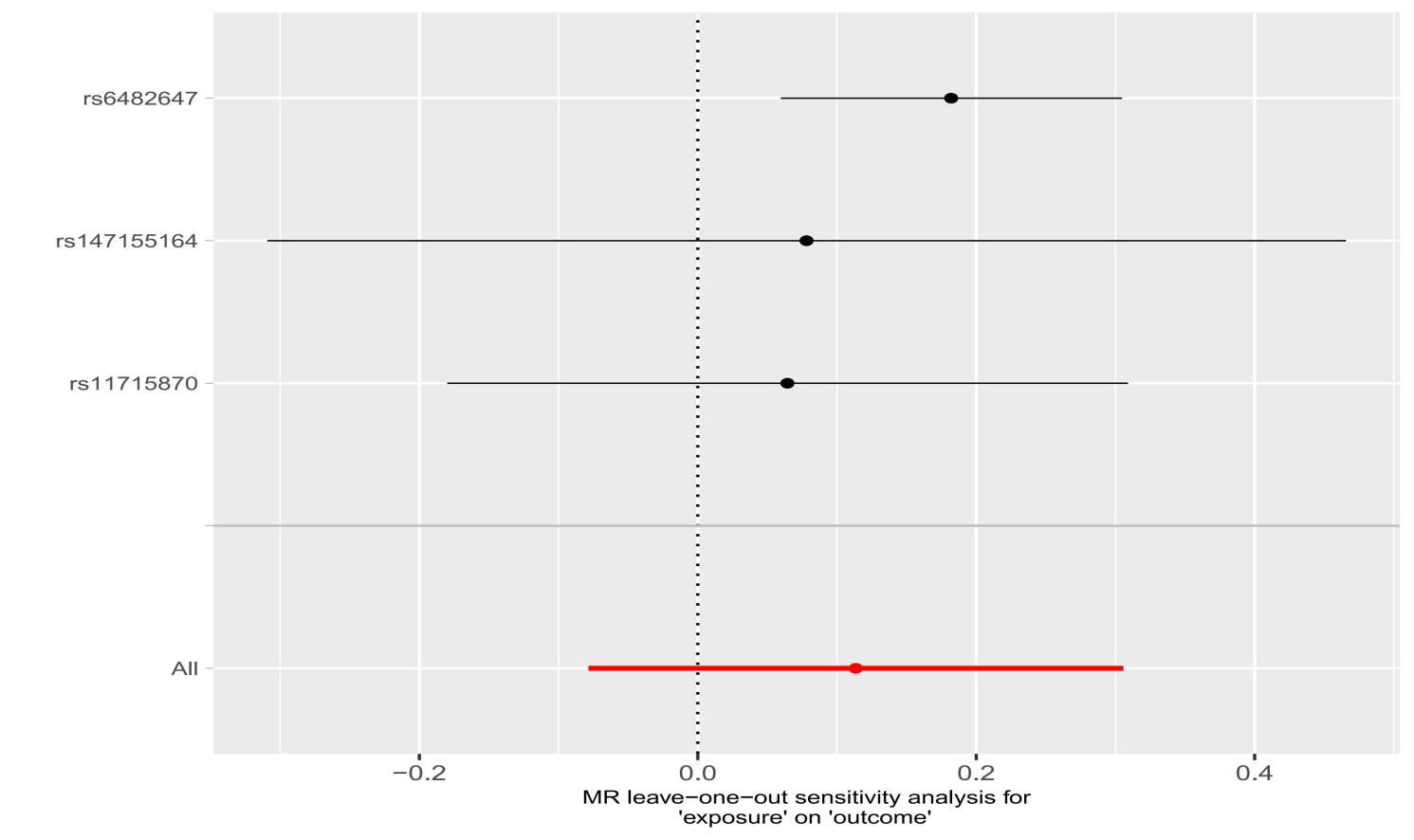


D


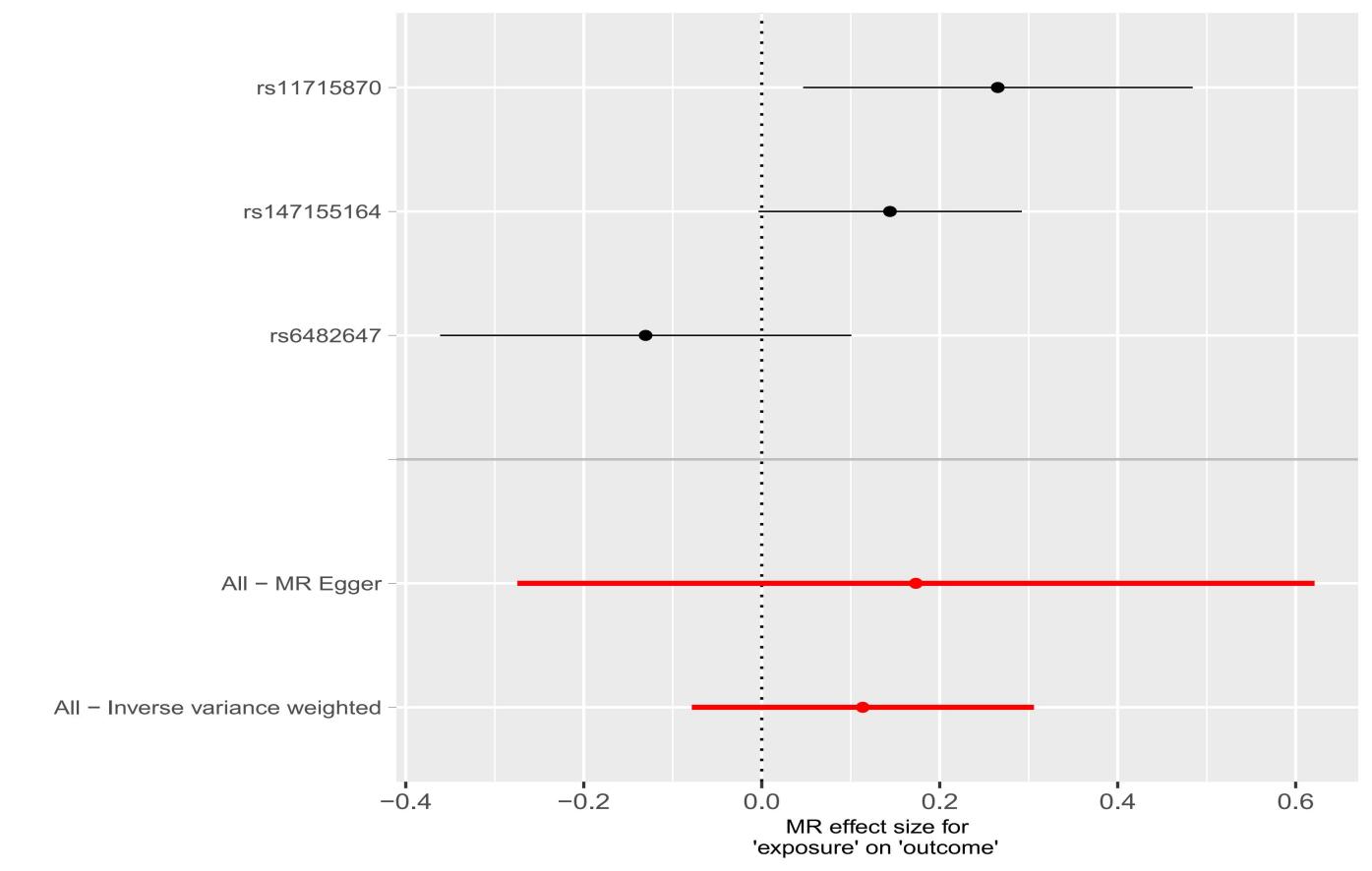


C


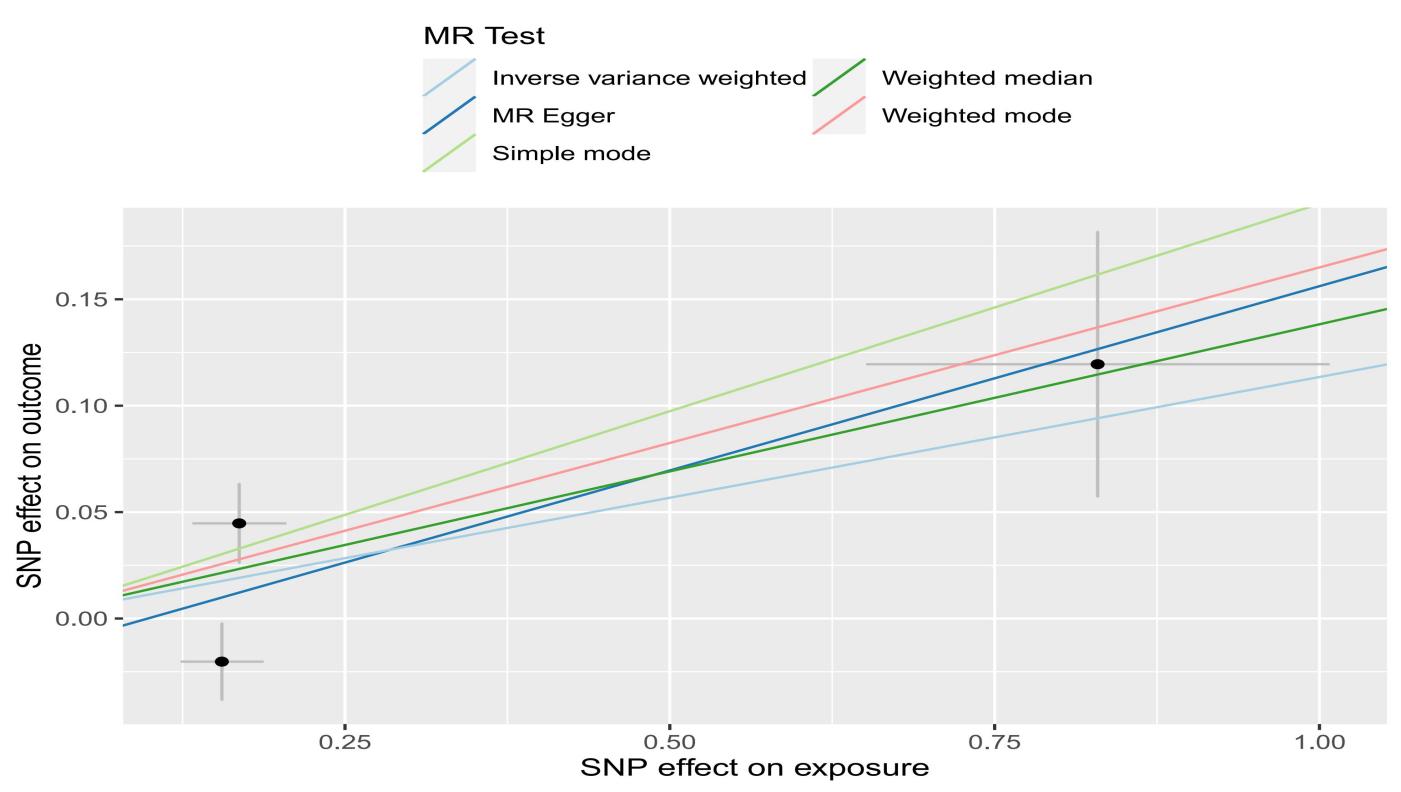


A

B

**Figure. S16** The causal effect of epilepsy (ILAE) on COVID-19 (infection). (A) Scatter plot, (B) Funnel plot, (C) Forest plot, and (D) Leave one out plot.


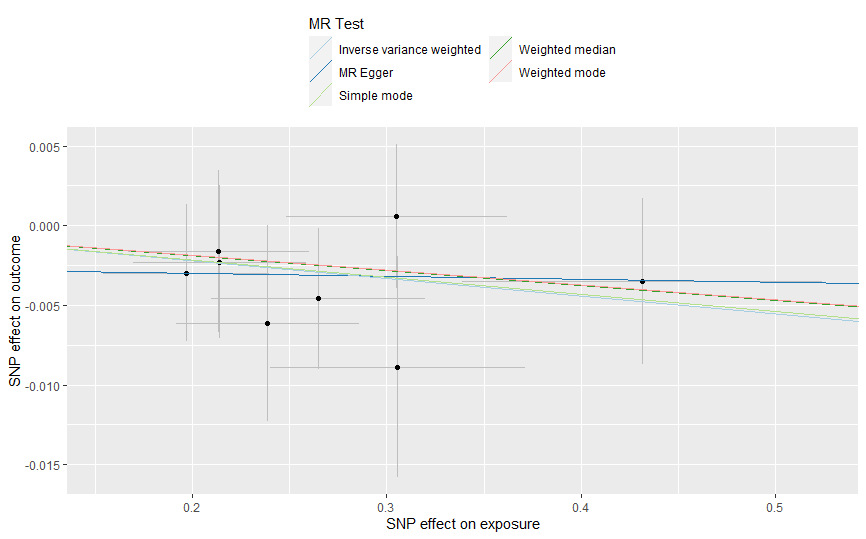

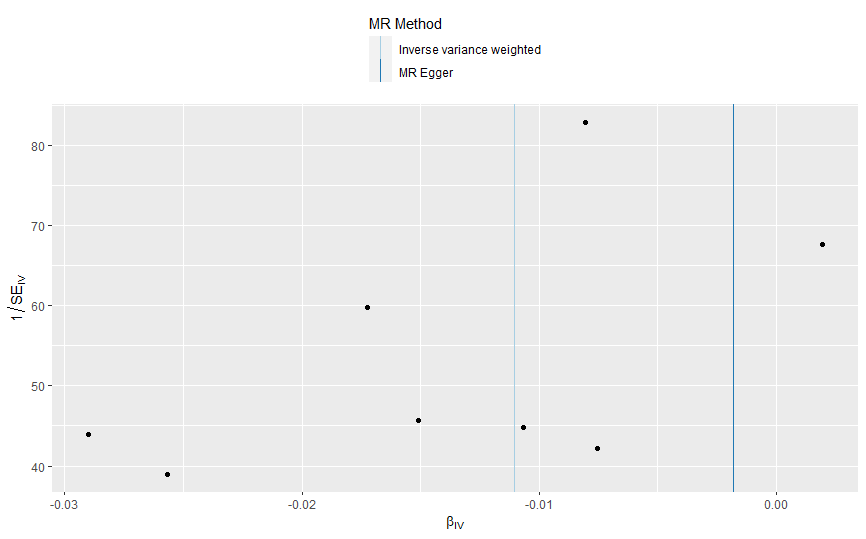

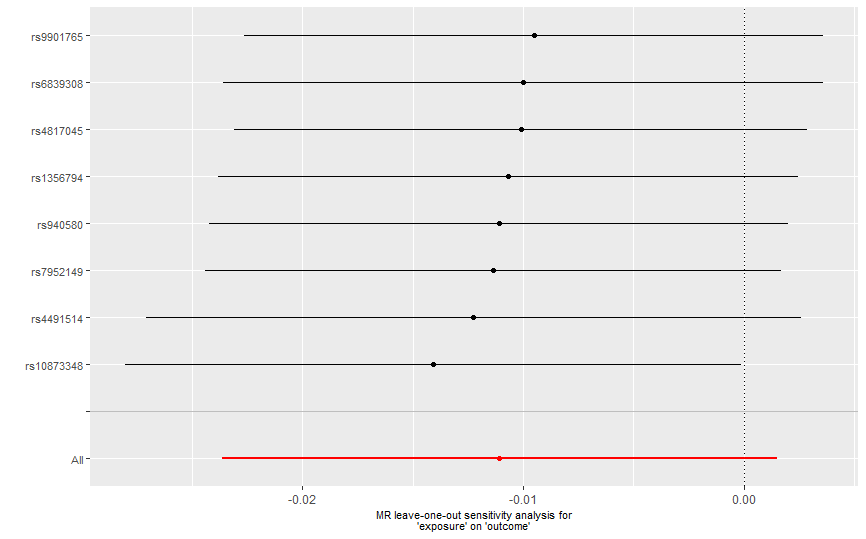

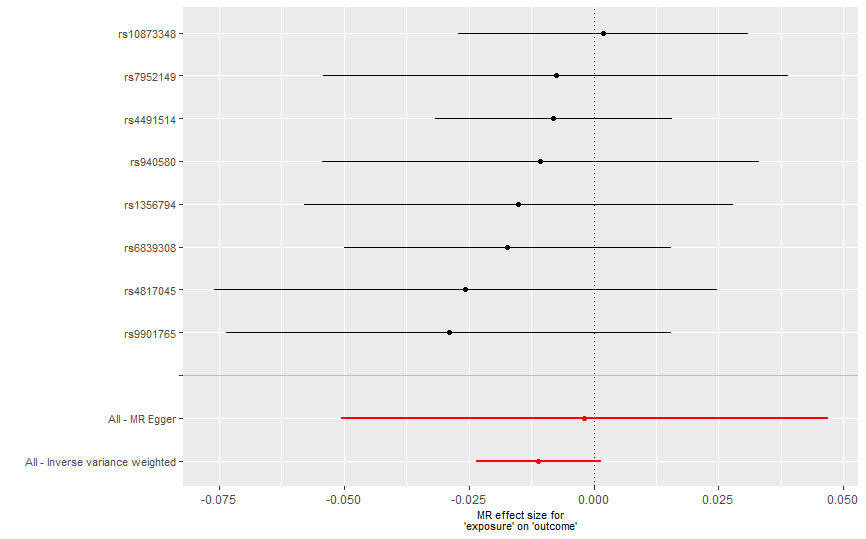


C

D

B

A

**Figure. S17** The causal effect of epilepsy **(ILAE) on COVID-19 (hospitalization). (A) Scatter plot, (B) Fun**nel plot, (C) Forest plot, and (D) Leave one out plot.


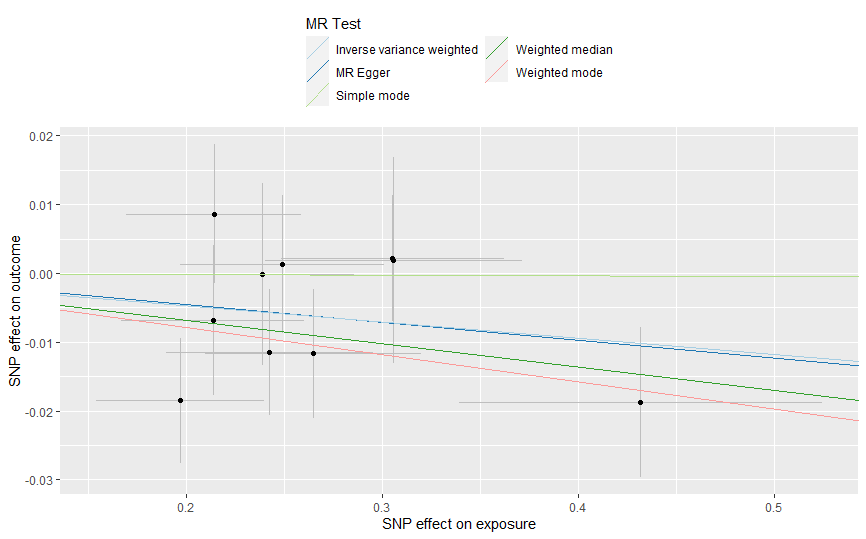

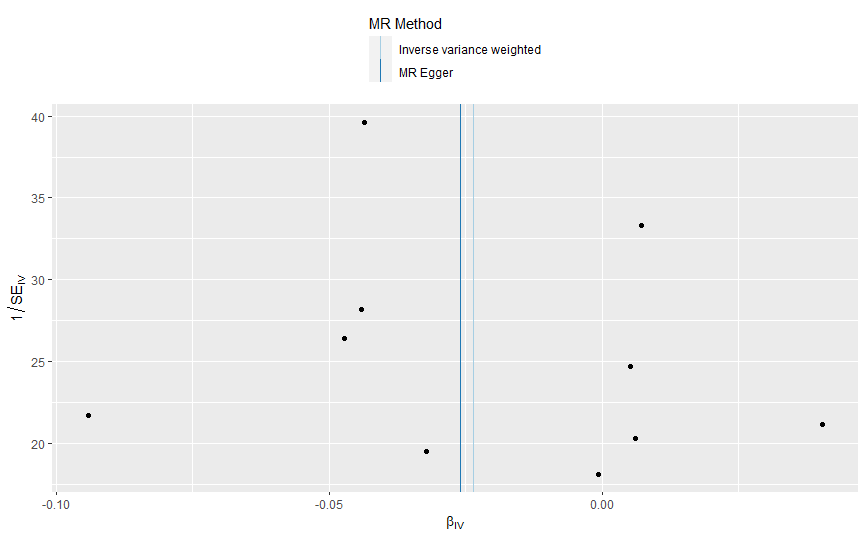

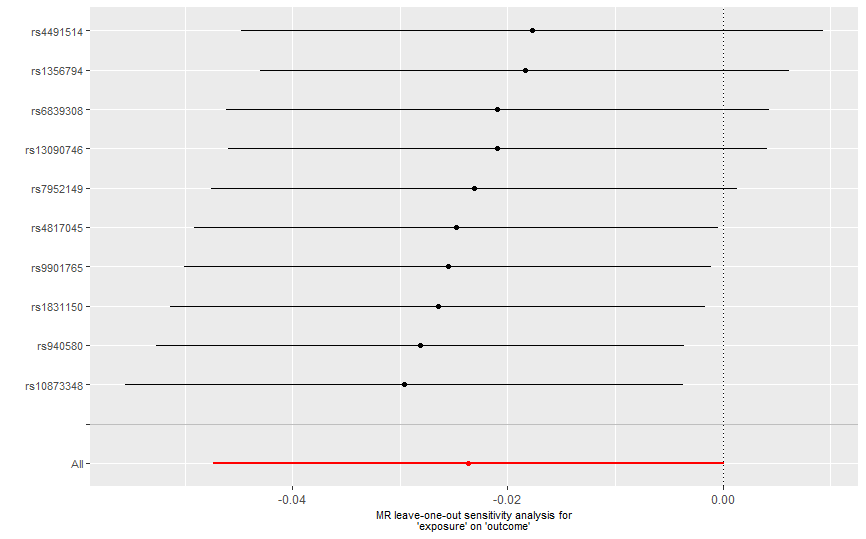

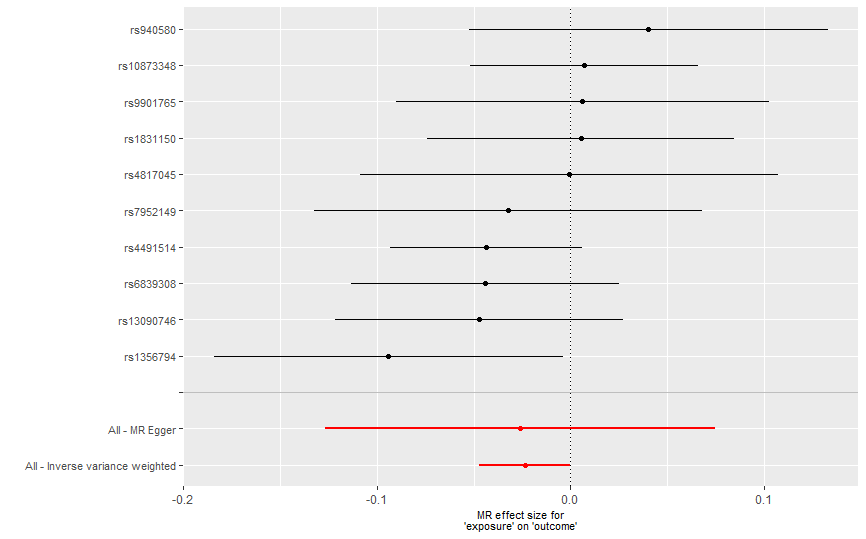


C

D

B

A

**Figure. S18** The causal effect of epilepsy (ILAE) on COVID-19 (severity). (A) Scatter plot, (B) Funnel plot, (C) Forest plot, and (D) Leave one out plot.


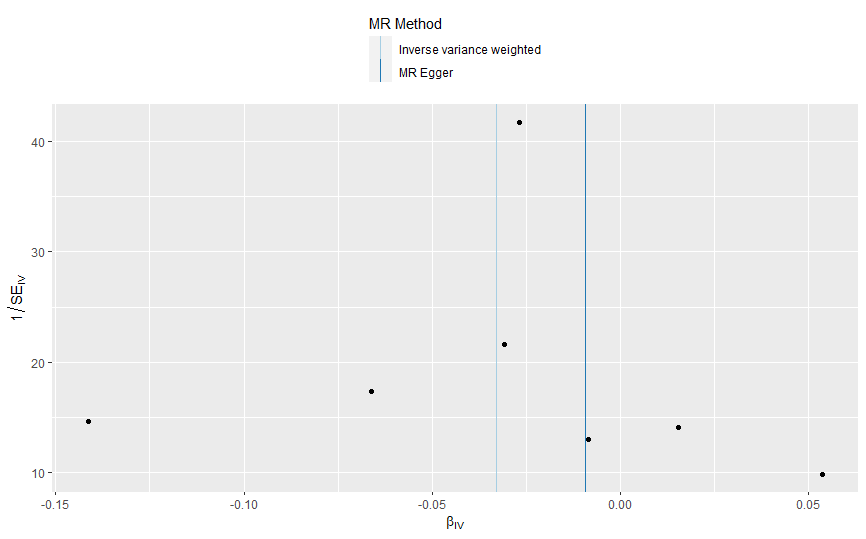

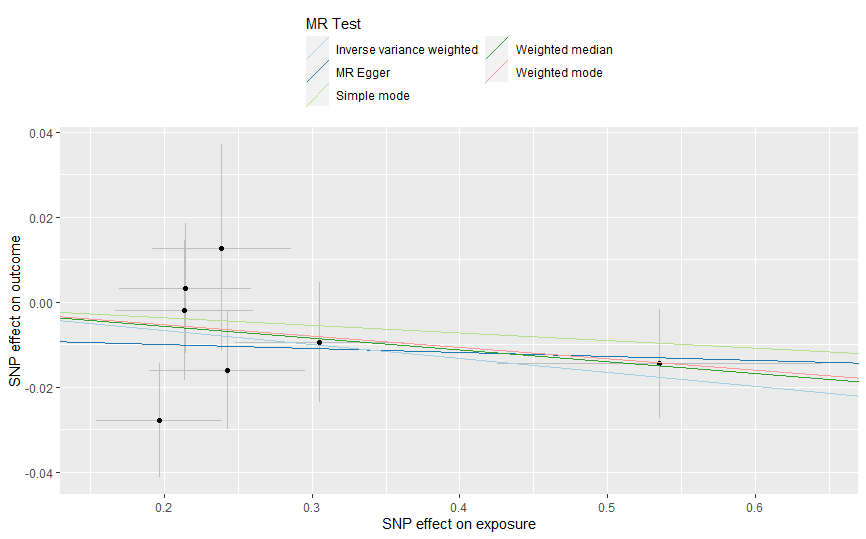

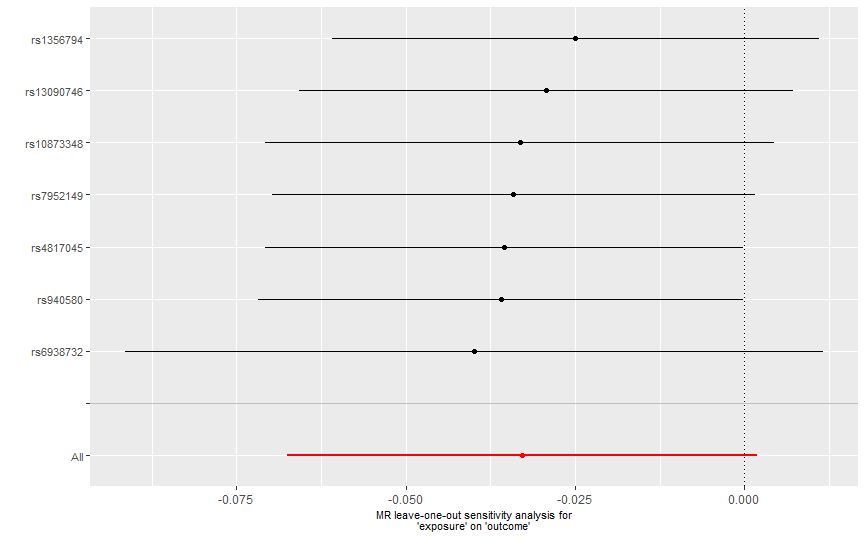

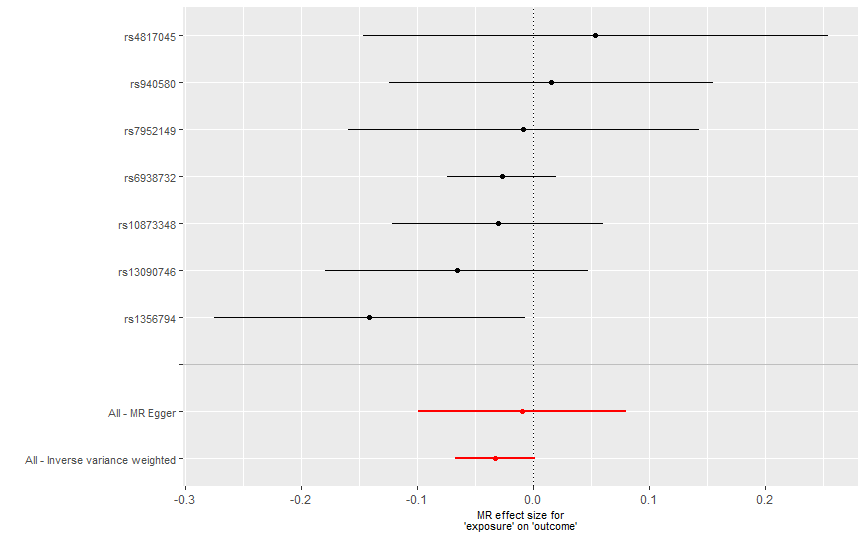


D

C

A

B

**Figure. S19** The causal effect of focal epilepsy (ILAE) on COVID-19 (infection). (A) Scatter plot, (B) Funnel plot, (C) Forest plot, and (D) Leave one out plot.


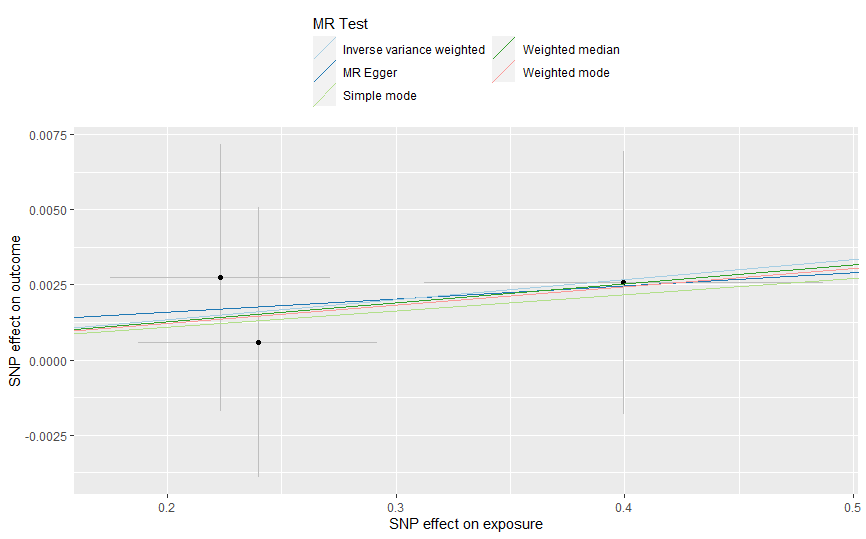

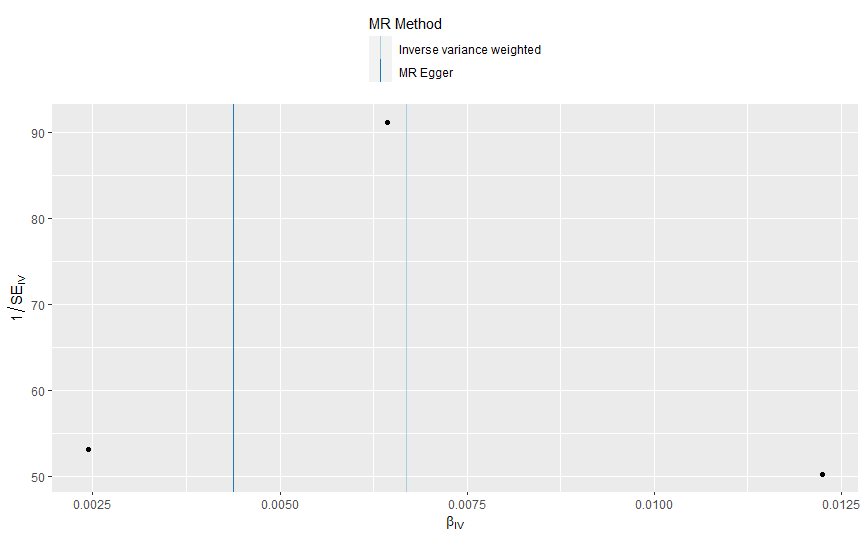

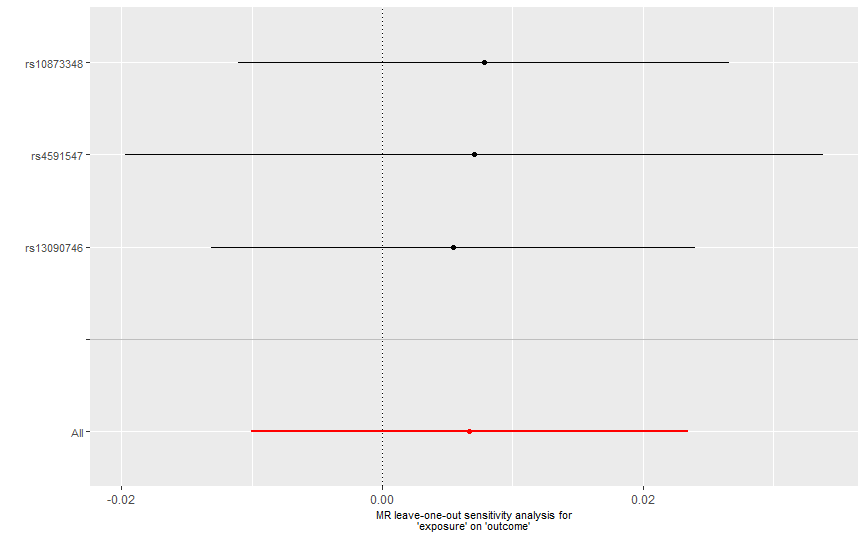

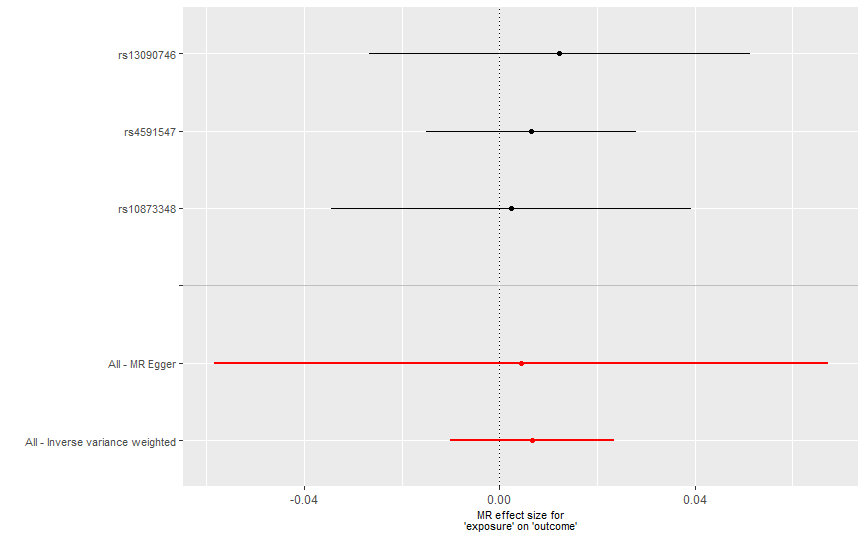


B

A

C

D

**Figure. S20** The causal effect of focal epilepsy (ILAE) on COVID-19 (hospitalization). (A) Scatter plot, (B) Funnel plot, (C) Forest plot, and (D) Leave one out plot.


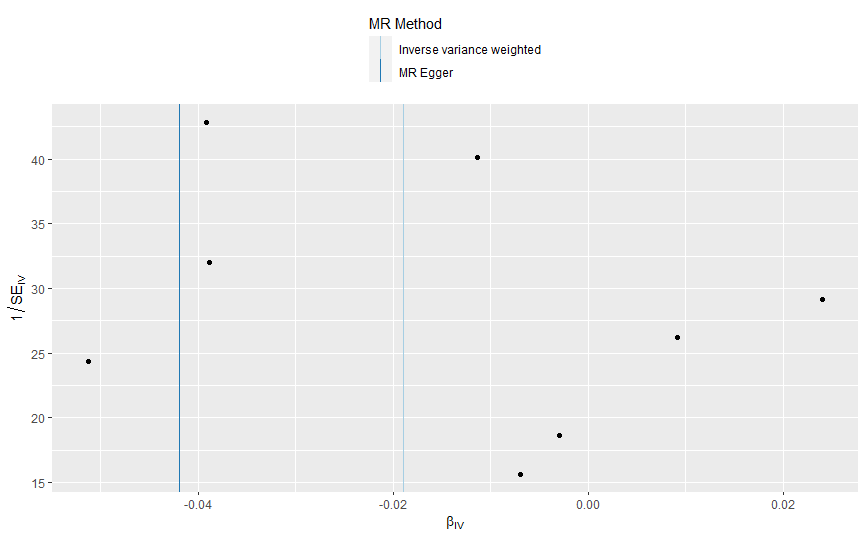

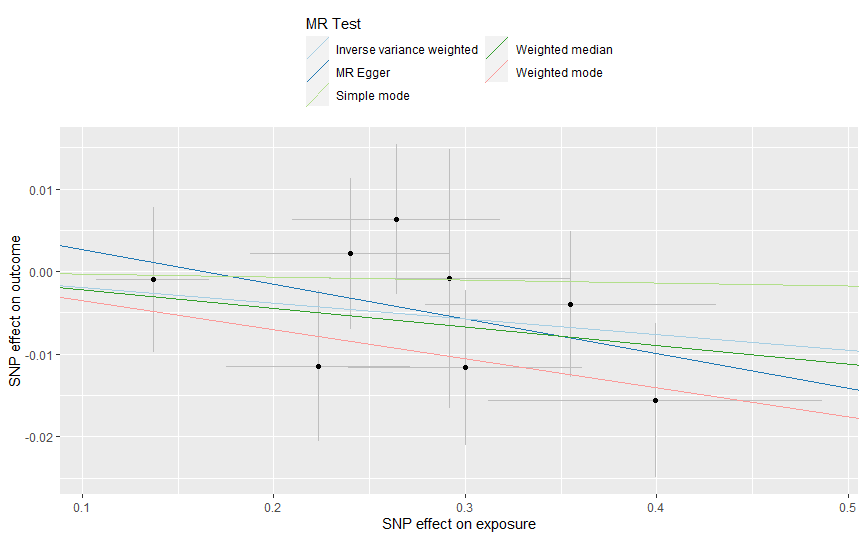

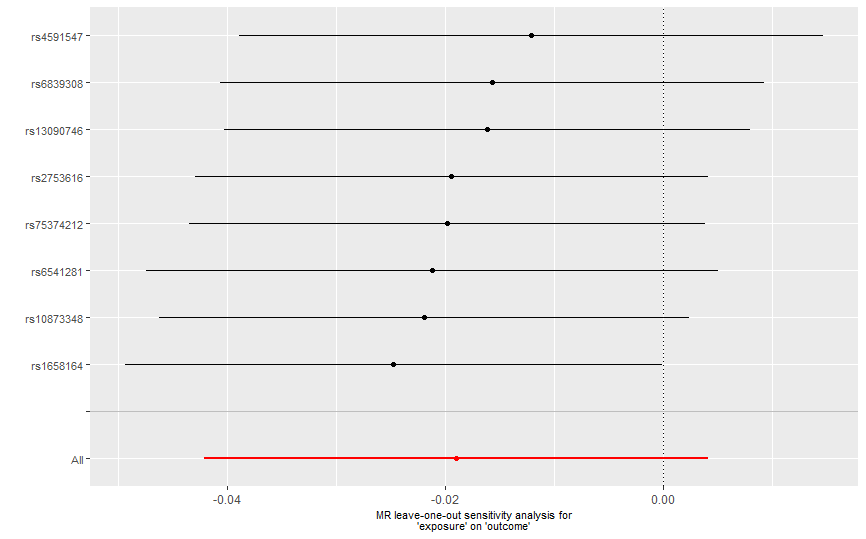

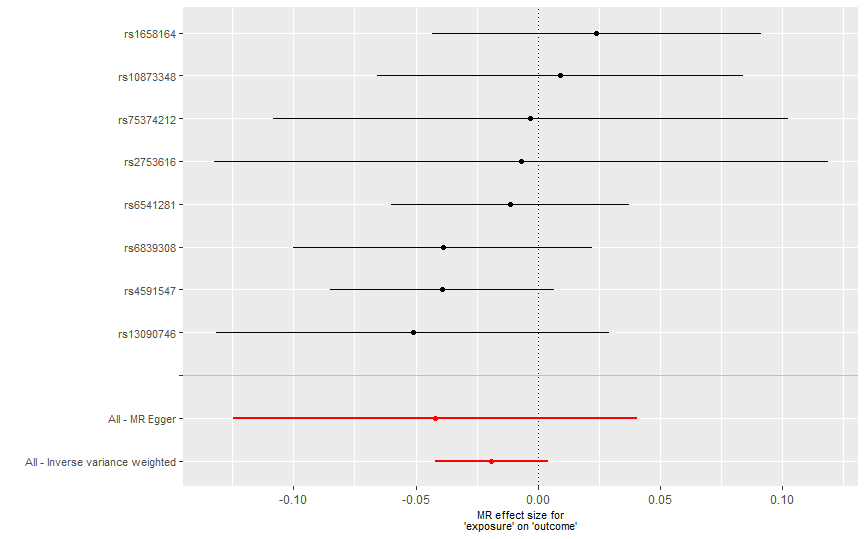


A

B

D

C

**Figure. S21** The causal effect of focal epilepsy (ILAE) on COVID-19 (severity). (A) Scatter plot, (B) Funnel plot, (C) Forest plot, and (D) Leave one out plot.


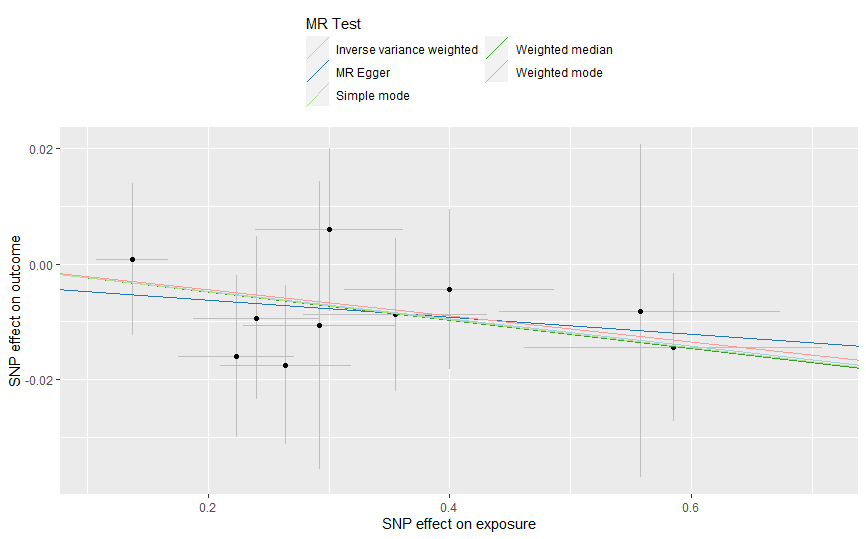

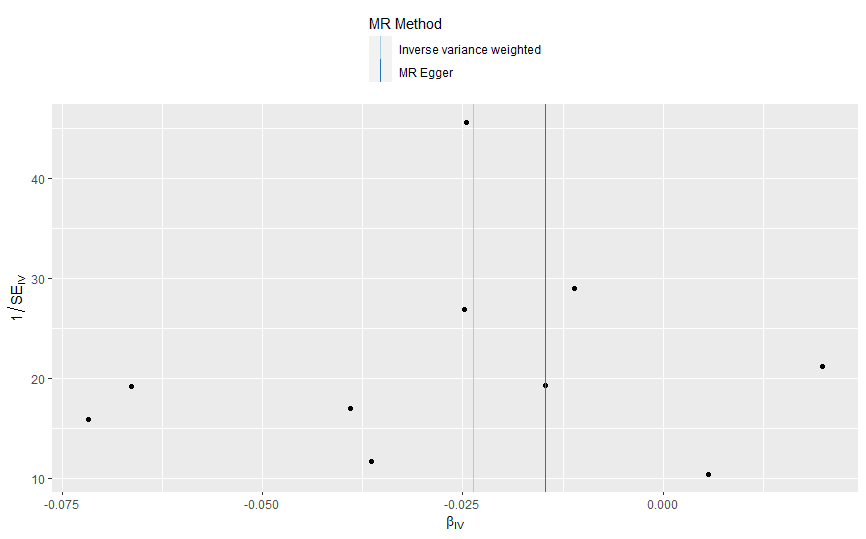

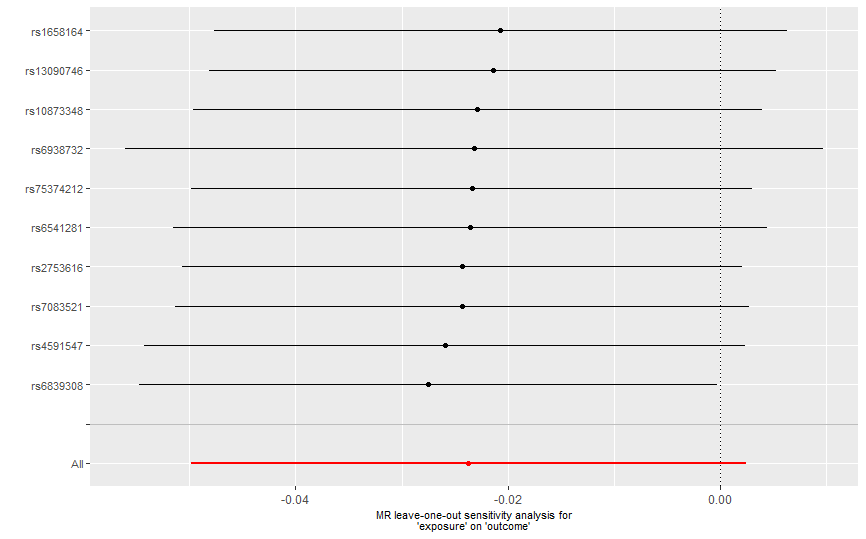

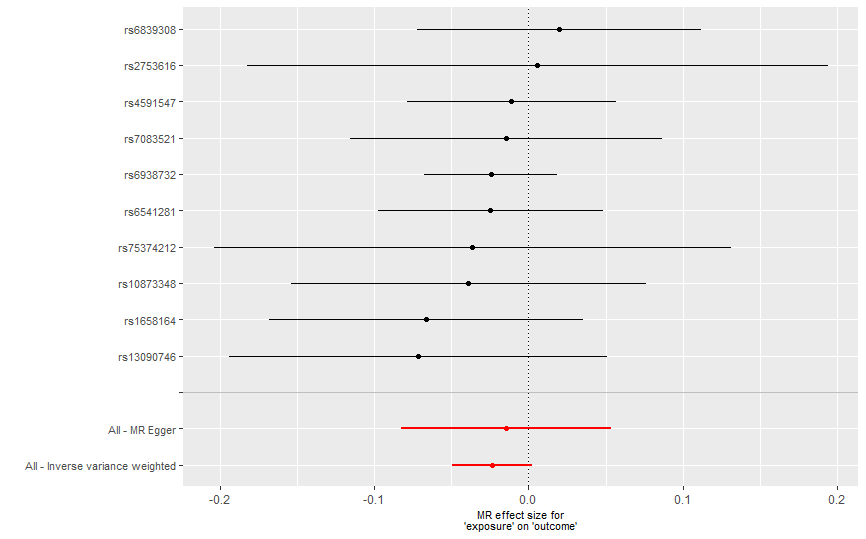


C

D

B

A

**Figure. S22** The causal effect of generalized (ILAE) epilepsy on COVID-19 (infection). (A) Scatter plot, (B) Funnel plot, (C) Forest plot, and (D) Leave one out plot.


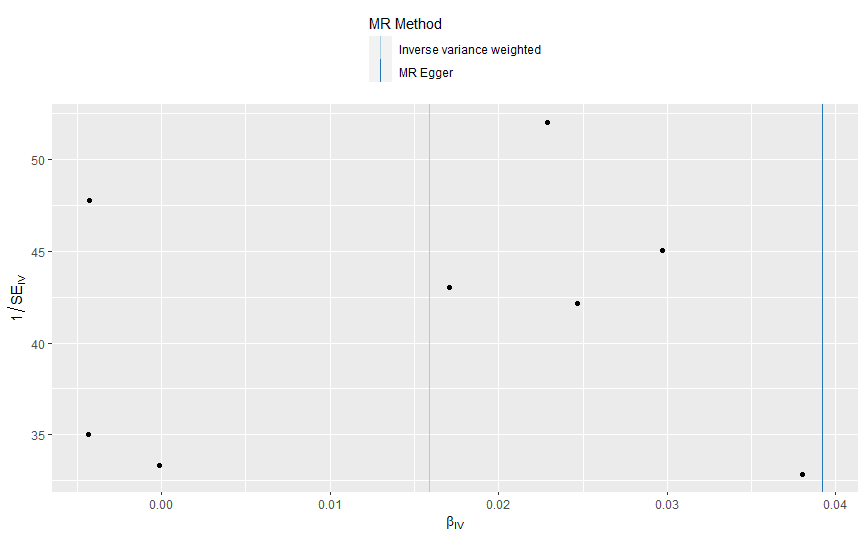

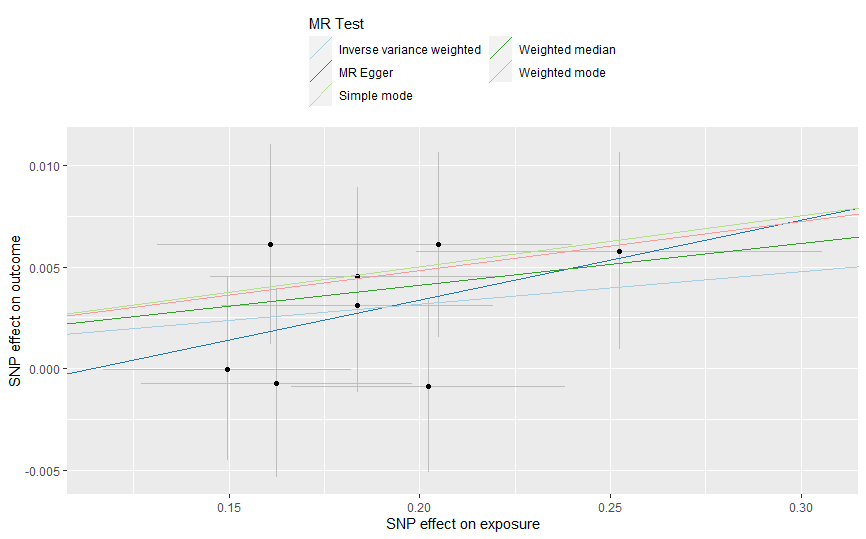

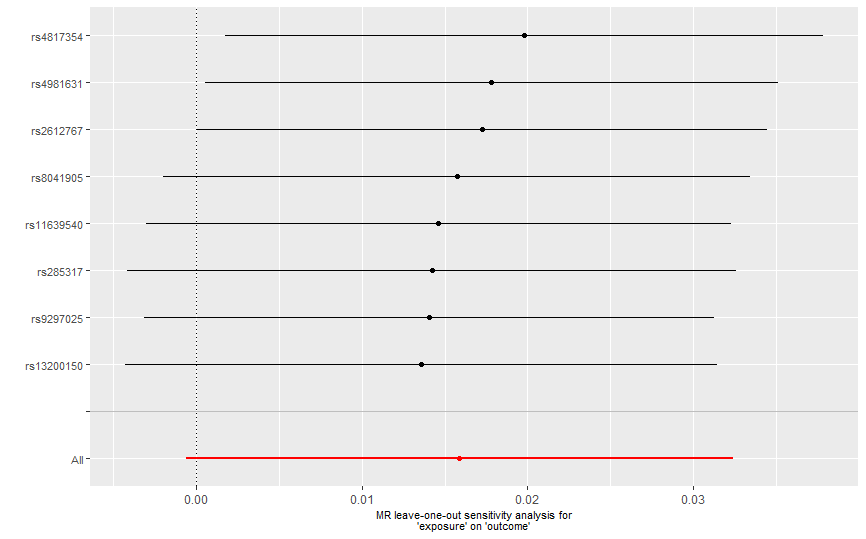

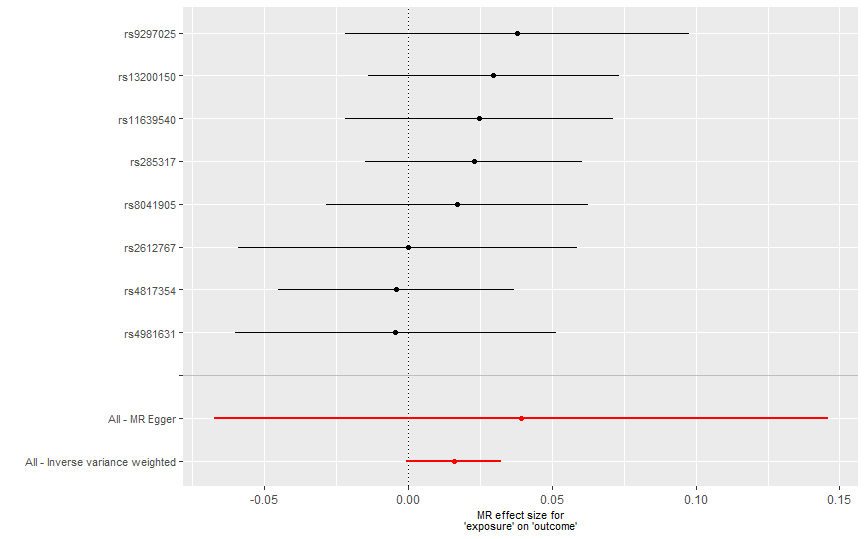


D

C

A

B

**Figure. S23** The causal effect of generalized epilepsy (ILAE) on COVID-19 (hospitalization). (A) Scatter plot, (B) Funnel plot, (C) Forest plot, and (D) Leave one out plot.


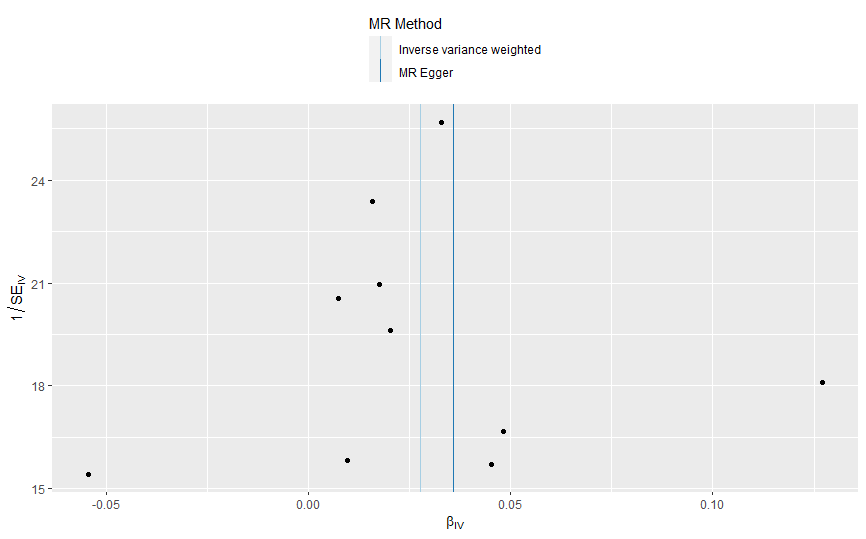

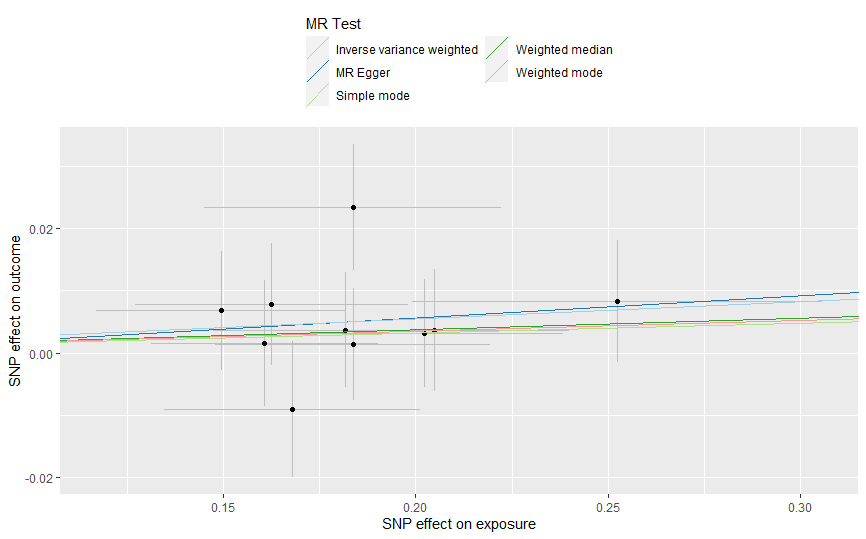

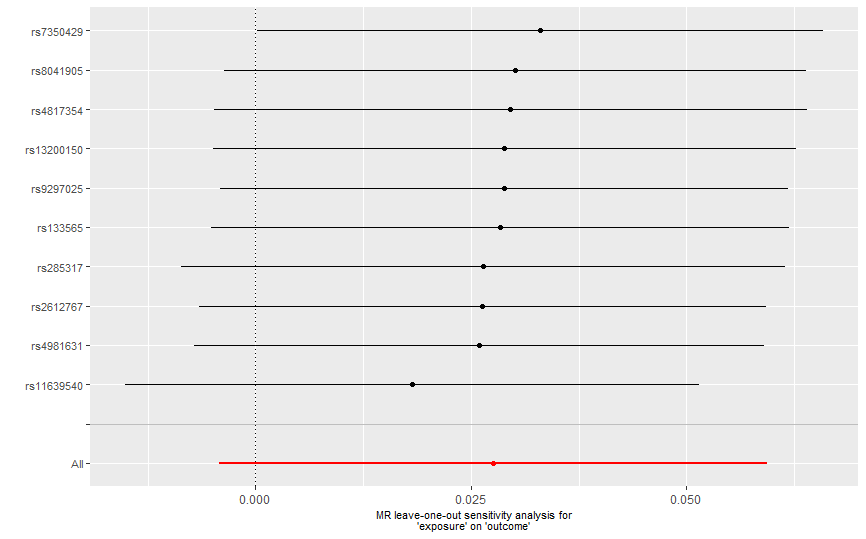

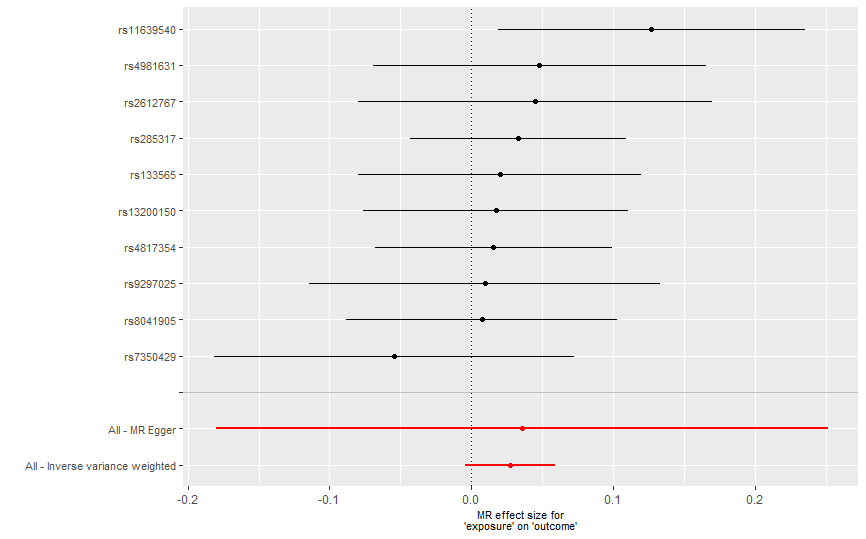


A

B

D

C

**Figure. S24** The causal effect of generalized epilepsy (ILAE) on COVID-19 (severity). (A) Scatter plot, (B) Funnel plot, (C) Forest plot, and (D) Leave one out plot.


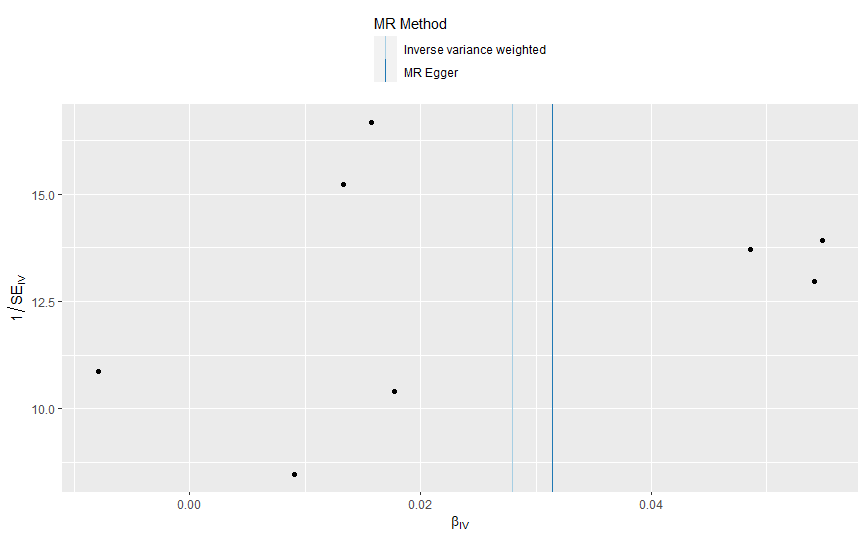

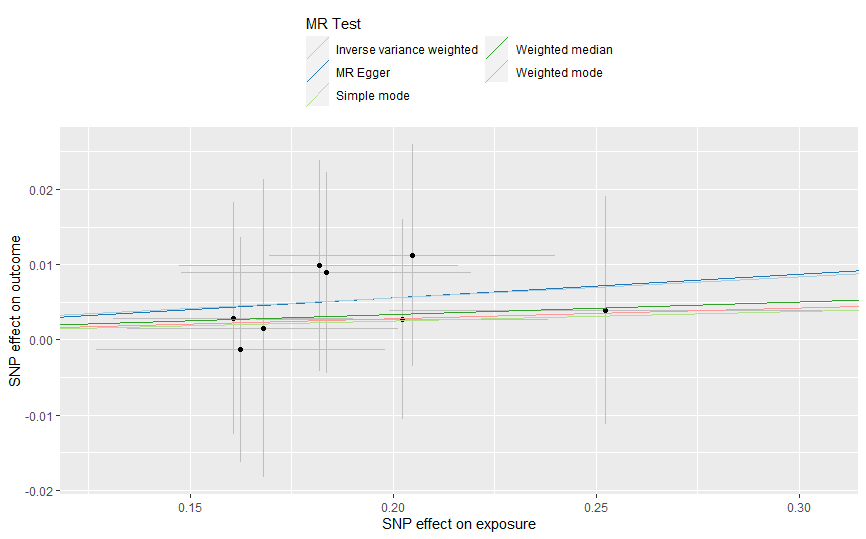

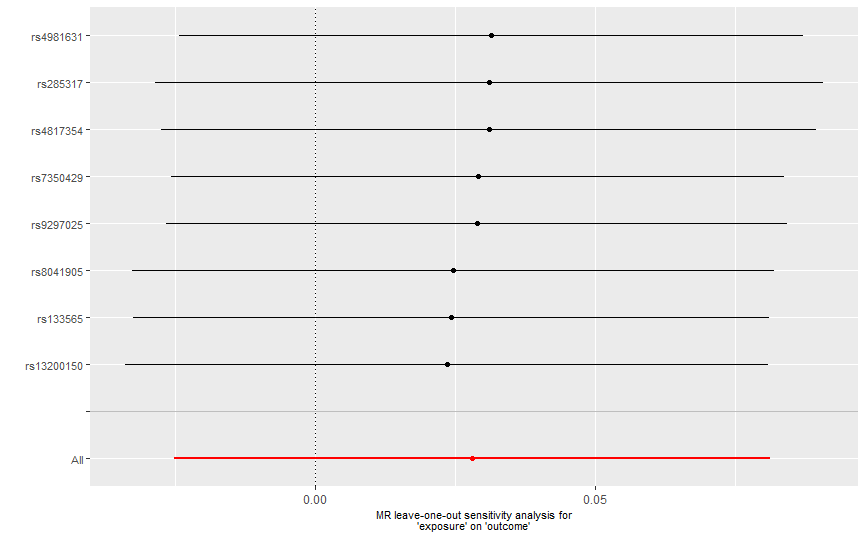

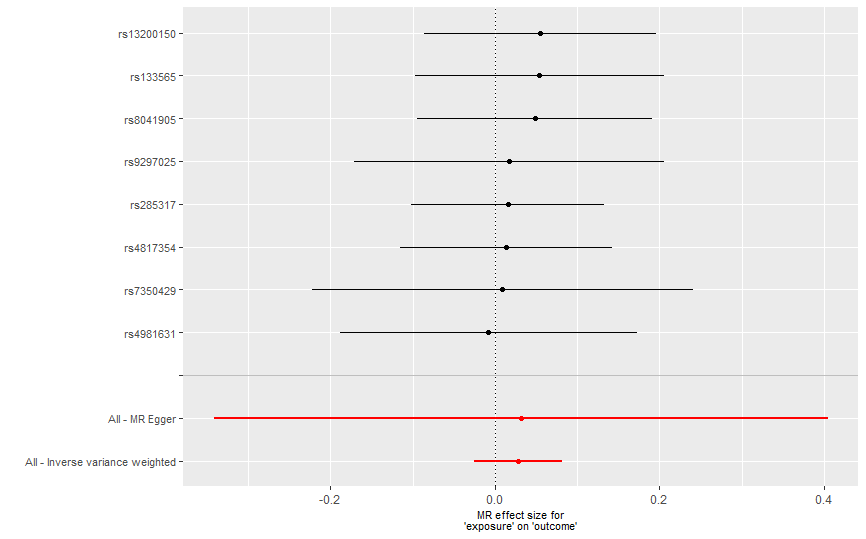


A

B

D

C
