# Supplementary material for: The association between epilepsy and COVID-19: analysis based on Mendelian randomization and FUMA
Source: Front Neurosci. 2023 Sep 15;17:1235822. doi: 10.3389/fnins.2023.1235822 (PMC10540302; doi:10.3389/fnins.2023.1235822)
Supplement: Supplementary file 1 [file Data_Sheet_1.ZIP › Supplementary Materials:Table S1-7.docx]

**Supplementary Material**

**Table S1 Sensitivity analysis of the associations of COVID-19 (infection, hospitalization, severity) on epilepsy and its substypes.**

| **Exposure** | **outcome** | **IVW**  **OR (95% CI)**  **P value** | **MR-Egger**  **OR (95% CI)**  **P value** | **Weighted median**  **OR (95% CI)**  **P value** | **Simple mode**  **OR (95% CI)**  **P value** | **Weighted mode**  **OR (95% CI)**  **P value** |
| --- | --- | --- | --- | --- | --- | --- |
| epilepsy and its subtypes database from FinnGen consortium | | | | | | |
| COVID-19 (infection) | epilepsy | 1.5306 (1.1676, 2.0062）  0.0021 | 1.3625 (0.8388, 2.2130)  0.2579 | 1.4247 (1.0155, 1.999)  0.0404 | 1.2563 (0.7049, 2.239)  0.4644 | 1.3860(0.9590, 2.0032)  0.1259 |
|  | focal epilepsy | 2.5161 (0.6269, 3.098)  0.1931 | 2.6106 (1.0081, 3.1747)  0.3934 | 2.0306 (0.3851, 2.7079)  0.4037 | 1.9637 (0.1870, 2.6170)  0.5981 | 1.9807 (0.2202, 2.8189)  0.5687 |
|  | generalized epilepsy | 2.1155 (1.1734, 3.8139)  0.0127 | 2.6926 (0.9214, 3.8689)  0.1132 | 1.9847 (0.9273, 4.2477)  0.0774 | 1.0382 (0.3088, 3.4903)  0.9531 | 2.3665 (1.0812, 5.1794)  0.0632 |
| COVID-19 (hospitalization) | epilepsy | 1.0934 (1.0097, 1.1841)  0.0281 | 1.1029 (0.9802, 1.2410)  0.1277 | 1.0967 (0.9947, 1.2091)  0.0640 | 1.0605 (0.8755, 1.2846)  0.5579 | 1.0924 (0.9852, 1.2111)  0.1156 |
|  | focal epilepsy | 1.5846 (1.0099, 2.4863)  0.0452 | 1.4083 (0.2793, 4.0994)  0.6906 | 1.3059 (0.7447, 2.2902)  0.3517 | 1.2761 (0.5268, 3.0907)  0.6038 | 1.3054 (0.6421, 2.6540)  0.4826 |
|  | generalized epilepsy | 1.2281 (1.0513, 1.4346)  0.0096 | 1.3471 (1.0543, 1.7212)  0.0291 | 1.2333 (1.0019, 1.5180)  0.0479 | 1.2394 (0.9951, 1.5437)  0.07138 | 1.2325 (1.0049, 1.5116)  0.0592 |
| COVID-19 (severity) | epilepsy | 1.2454 (1.0850, 1.4295)  0.0018 | 1.2011 (0.8078 to 1.7858)  0.4164 | 1.2076 (1.0146, 1.4374)  0.0338 | 1.2399 (0.9646, 1.5936)  0.1540 | 1.2139 (0.9638, 1.5290)  0.1606 |
|  | focal epilepsy | 1.6818 (1.1478, 2.4642)  0.0077 | 1.2250 (0.2704, 5.5498)  0.8029 | 1.4639 (0.9014, 2.3773)  0.1234 | 1.3739 (0.6710, 2.7079)  0.3942 | 1.3799 (0.7256, 2.6245)  0.3641 |
|  | generalized epilepsy | 1.1486 (1.0274, 1.2842)  0.0149 | 1.2032 (1.0063, 1.4385)  0.0575 | 1.1503 (1.0005, 1.3224）  0.0491 | 1.0358 (0.8042, 1.3341)  0.7881 | 1.1500 (0.9926, 1.3324)  0.0783 |
| epilepsy and its subtypes database from the International League Against epilepsy (ILAE) | | | | | | |
| COVID-19 (infection) | epilepsy | 1.3440 (1.0235,1.7649)  0.0334 | 1.1038 (0.3065, 3.9753）  0.9046 | 1.3059 (0.9641, 1.7689)  0.0847 | 1.3055 (0.8761, 1.9452)  0.3204 | 1.3010 (0.8978, 1.8851)  0.2989 |
|  | focal epilepsy | 1.0869 (0.9715, 1.2159)  0.1455 | 1.7709 (1.0665, 2.9405）  0.2707 | 1.0840 (0.9954, 1.1804)  0.0637 | 1.2802 (1.0544, 1.5545)  0.1301 | 1.0618 (0.9730, 1.1587)  0.3109 |
|  | generalized epilepsy | 1.1245 (1.0444, 1.2108)  0.0019 | 1.1306 (0.5419, 2.3587)  0.7746 | 1.1220 (1.0284, 1.2241)  0.0096 | 1.1290 (0.9465, 1.3467)  0.2703 | 1.1211 (1.0322, 1.2177)  0.0731 |
| COVID-19 (hospitalization) | epilepsy | 1.7381 (1.0467, 2.8862)  0.0326 | 2.7070 (0.5161, 4.1975)  0.2919 | 1.6491 (0.8873, 3.0649)  0.1137 | 1.7082 (0.7103, 4.1081)  0.2769 | 1.6522 (0.7924, 3.4448)  0.2290 |
|  | focal epilepsy | 1.7549 (1.1063, 2.7838)  0.0169 | 2.3981 (0.3966, 4.4988)  0.3775 | 1.8087 (1.0234, 3.1966)  0.0414 | 1.5896 (0.7734, 3.2671)  0.2477 | 1.6723 (0.8673, 3.2242)  0.1686 |
|  | generalized epilepsy | 1.1827 (1.0215, 1.3693）  0.0248 | 1.2757 (0.8415, 1.9338)  0.2951 | 1.1842 (1.0433, 1.3442)  0.0089 | 1.1670 (0.9689, 1.4056)  0.1477 | 1.1827 (1.0479, 1.3349)  0.0299 |
| COVID-19 (severity) | epilepsy | 1.2724 (1.0347, 1.5647)  0.0224 | 1.1579 (0.5956, 2.2509)  0.6807 | 1.0953 (0.8598, 1.3953)  0.4612 | 1.0324 (0.6823, 1.5621)  0.8843 | 1.0520 (0.7171, 1.5434）  0.8028 |
|  | focal epilepsy | 1.6598 (1.2572, 2.1914)  0.0003 | 1.4228 (0.3953, 5.1214)  0.6181 | 1.6419 (1.1548, 2.3346)  0.0058 | 1.2767 (0.7017, 2.3227)  0.4601 | 1.6479 (1.1666, 2.3279)  0.0365 |
|  | generalized epilepsy | 1.0439 (1.0159, 1.0728)  0.0019 | 1.0786 (0.9579, 1.2144)  0.2796 | 1.0455 (0.9878, 1.1064)  0.1244 | 1.0445 (0.9739, 1.1201)  0.2771 | 1.0445 (1.0069, 1.0834)  0.0674 |

**Table S2 The causal effect of epilepsy and its subtypes on Covid-19 (infection, hospitalization, severity).**

| **Exposure** | **Outcome** | **nSNPs** | **Method** | **OR (95% CI)** | ***P* value** | ***P_FDR_*** |
| --- | --- | --- | --- | --- | --- | --- |
| epilepsy and its subtypes database from FinnGen consortium | | | | | | |
| epilepsy | COVID-19 (infection) | 7 | IVW | 0.9608 (0.9231, 1.0001) | 0.0505 | 0.4545 |
|  | COVID-19 (hospitalization) | 7 | IVW | 0.9171 (0.8353, 1.0069) | 0.0694 | 0.1785 |
|  | COVID-19 (severity) | 11 | IVW | 0.9144 (0.8189,1.0211) | 0.1119 | 0.1678 |
| focal epilepsy, | COVID-19 (infection) | 7 | IVW | 1.0032 (0.9920, 1.0145) | 0.5753 | 0.5753 |
|  | COVID-19 (hospitalization) | 5 | IVW | 0.9614 (0.9243, 1.0000) | 0.0501 | 0.9014 |
|  | COVID-19 (severity) | 7 | IVW | 0.9611 (0.9212, 1.0026) | 0.0661 | 0.1983 |
| generalized epilepsy | COVID-19 (infection) | 3 | IVW | 1.0124 (0.9804, 1.0455) | 0.4513 | 0.4778 |
|  | COVID-19 (hospitalization) | 3 | IVW | 1.0523 (0.9753, 1.1354) | 0.1887 | 0.2613 |
|  | COVID-19 (severity) | 3 | IVW | 1.1202 (0.9245, 1.3572) | 0.2466 | 0.3171 |
| epilepsy and its subtypes database from the International League Against epilepsy (ILAE) | | | | | | |
| epilepsy | COVID-19 (infection) | 8 | IVW | 0.9890 (0.9766, 1.0015) | 0.0844 | 0.1688 |
|  | COVID-19 (hospitalization) | 10 | IVW | 0.9766 (0.9537, 1.0001) | 0.0509 | 0.3054 |
|  | COVID-19 (severity) | 7 | IVW | 0.9677 (0.9347, 1.0019) | 0.0641 | 0.2308 |
| focal epilepsy | COVID-19 (infection) | 3 | IVW | 1.0067 (0.9900, 1.0237) | 0.4348 | 0.4892 |
|  | COVID-19 (hospitalization) | 8 | IVW | 0.9812 (0.9588, 1.0042) | 0.1079 | 0.1766 |
|  | COVID-19 (severity) | 10 | IVW | 0.9766 (0.9514, 1.0024) | 0.0754 | 0.1697 |
| generalized epilepsy | COVID-19 (infection) | 8 | IVW | 1.0160 (0.9994, 1.0329) | 0.0595 | 0.2676 |
|  | COVID-19 (hospitalization) | 10 | IVW | 1.0279 (0.9957, 1.0612) | 0.0896 | 0.1613 |
|  | COVID-19 (severity) | 8 | IVW | 1.0283 (0.9751, 1.0845) | 0.3030 | 0.3636 |

MR, Mendelian randomization; OR, odds ratio; Type-I error rate (α) was set as 0.05.

**Table S3. Sensitivity analysis of epilepsy and its subtypes on Covid-19 (infection, hospitalization, severity).**

| **Exposure** | **outcome** | **IVW**  **OR (95% CI)**  **P value** | **MR-Egger**  **OR (95% CI)**  **P value** | **Weighted median**  **OR (95% CI)**  **P value** | **Simple mode**  **OR (95% CI)**  **P value** | **Weighted mode**  **OR (95% CI)**  **P value** |
| --- | --- | --- | --- | --- | --- | --- |
| epilepsy and its subtypes database from FinnGen consortium | | | | | | |
| epilepsy | COVID-19 (infection) | 0.9608 (0.9231, 1.0001)  0.0505 | 0.9627 (0.8927, 1.0381)  0.3681 | 0.9547 (0.9057, 1.0064)  0.0848 | 0.9487 (0.8786, 1.0244)  0.2275 | 0.9519 (0.8901, 1.0179)  0.1999 |
|  | COVID-19 (hospitalization) | 0.9171 (0.8353, 1.0069)  0.0694 | 0.8937 (0.7091, 1.1264)  0.3849 | 0.9759 (0.8625, 1.1044)  0.6999 | 0.9918 (0.8232, 1.1952)  0.9345 | 0.9925 (0.8257, 1.1929)  0.9388 |
|  | COVID-19 (severity) | 0.9144 (0.8189, 1.0211)  0.1119 | 0.9492 (0.7736, 1.1648)  0.6297 | 0.9159 (0.7865, 1.0667)  0.2588 | 0.8963 (0.7131, 1.1265)  0.3699 | 0.9027 (0.7399, 1.1013)  0.3369 |
| focal epilepsy | COVID-19 (infection) | 1.0032 (0.9920, 1.0145)  0.5753 | 1.0031 (0.9849, 1.0217)  0.7507 | 1.0028 (0.9885, 1.0174)  0.7116 | 1.0006 (0.9812, 1.0204)  0.9578 | 1.0025 (0.9879, 1.0173)  0.7584 |
|  | COVID-19 (hospitalization) | 0.9614 (0.9243, 1.0000)  0.0501 | 0.9845 (0.9121, 1.0627)  0.7153 | 0.9636 (0.9153, 1.0145)  0.1582 | 0.9381 (0.8729, 1.0082)  0.1572 | 0.97188 (0.9123, 1.0353)  0.4264 |
|  | COVID-19 (severity) | 0.9611 (0.9212, 1.0026)  0.0661 | 0.9677 (0.8998, 1.0409)  0.4190 | 0.9739 (0.9281, 1.0221)  0.2833 | 0.9043 (0.8199, 0.9974)  0.0909 | 0.9821 (0.9385, 1.0276)  0.4639 |
| generalized epilepsy | COVID-19 (infection) | 1.0124 (0.9804, 1.0455)  0.4513 | 1.0128 (0.9531, 1.0764)  0.7519 | 1.0124 (0.9768, 1.0492)  0.5007 | 1.0122 (0.9710, 1.0552)  0.6242 | 1.0122 (0.9709, 1.0553)  0.6253 |
|  | COVID-19 (hospitalization) | 1.0523 (0.9753, 1.1354)  0.1887 | 1.1305 (0.9921, 1.2883)  0.3169 | 1.0721 (0.9816, 1.1709)  0.1216 | 1.0845 (0.9518, 1.2357)  0.3474 | 1.0909 (0.9718, 1.2247)  0.2781 |
|  | COVID-19 (severity) | 1.1202 (0.9245, 1.3572)  0.2466 | 1.1891 (0.7599, 1.8605)  0.5870 | 1.1483 (0.9896, 1.3324)  0.0685 | 1.2151 (1.0009, 1.4753)  0.1878 | 1.1794 (0.9929, 1.4008)  0.2009 |
| epilepsy and its subtypes database from the International League Against epilepsy (ILAE) | | | | | | |
| epilepsy | COVID-19 (infection) | 0.9890 (0.9766, 1.0015)  0.0844 | 0.9982 (0.9508, 1.0480)  0.9439 | 0.9907 (0.9746, 1.0071)  0.2637 | 0.9893 (0.9651, 1.0141)  0.4216 | 0.9907 (0.9713, 1.0106)  0.3894 |
|  | COVID-19 (hospitalization) | 0.9766 (0.9537, 1.0001)  0.0509 | 0.9744 (0.8807, 1.0779)  0.6278 | 0.9666 (0.9364, 0.9978)  0.0361 | 0.9990 (0.9435, 1.0579)  0.9746 | 0.9613 (0.9118, 1.0135)  0.1774 |
|  | COVID-19 (severity) | 0.9677 (0.9347, 1.0019)  0.0641 | 0.9907 (0.9057, 1.0837)  0.8470 | 0.9727 (0.9314, 1.0157)  0.2098 | 0.9822 (0.9242, 1.0439)  0.5852 | 0.9737 (0.9318, 1.0174)  0.2785 |
| focal epilepsy | COVID-19 (infection) | 1.0067 (0.9900, 1.0237)  0.4348 | 1.0043 (0.9432, 1.0695)  0.9137 | 1.0063 (0.9875, 1.0255)  0.5120 | 1.0054 (0.9831, 1.0283)  0.6831 | 1.0061 (0.9860, 1.0266)  0.6139 |
|  | COVID-19 (hospitalization) | 0.9812 (0.9588, 1.0042)  0.1079 | 0.9589 (0.8829, 1.0415)  0.3584 | 0.9779 (0.9478, 1.0089)  0.1604 | 0.9964 (0.9495, 1.0456)  0.8882 | 0.9654 (0.9282, 1.0042)  0.1229 |
|  | COVID-19 (severity) | 0.9766 (0.9514, 1.0024)  0.0754 | 0.9854 (0.9205, 1.0548)  0.6826 | 0.9759 (0.9420, 1.0110)  0.1762 | 0.9761 (0.9297, 1.0248)  0.3560 | 0.9776 (0.9404, 1.0163)  0.2830 |
| generalized epilepsy | COVID-19 (infection) | 1.0160 (0.9994, 1.0329)  0.0595 | 1.0399 (0.9347, 1.1570)  0.4987 | 1.0207（0.9995, 1.0424)  0.0559 | 1.0253 (0.9932, 1.0585)  0.1859 | 1.0243 (0.9926, 1.0571)  0.1835 |
|  | COVID-19 (hospitalization) | 1.0279(0.9957, 1.0612)  0.0896 | 1.0364 (0.8354, 1.2858)  0.7534 | 1.0188 (0.9772, 1.0622)  0.3818 | 1.0160 (0.9597, 1.0756)  0.5990 | 1.0174 (0.9625, 1.0755)  0.5565 |
|  | COVID-19 (severity) | 1.0283 (0.9751, 1.0845)  0.3030 | 1.0319(0.7106, 1.4986)  0.8744 | 1.0170 (0.9532, 1.0852)  0.6097 | 1.0127 (0.9289, 1.1042)  0.7823 | 1.0142 (0.9380, 1.0966)  0.7341 |

**Table S4**. **Heterogeneity and pleiotropy tests of epilepsy and its subtypes on Covid-19 (infection, hospitalization, severity).**

| **Exposure** | **outcome** | **Cochrane’s *Q* test** | | **MR-Egger intercept test** | | **MRPRESSO global test** |
| --- | --- | --- | --- | --- | --- | --- |
|  |  | **Q-value** | **PQ** | **Intercept** | **P-intercept** | **P value** |
| epilepsy and its subtypes database from FinnGen consortium | | | | | | |
| epilepsy | COVID-19 (infection) | 3.1667 | 0.7877 | -0.0002 | 0.9554 | 0.812 |
|  | COVID-19 (hospitalization) | 3.7059 | 0.7164 | 0.0026 | 0.8206 | 0.723 |
|  | COVID-19 (severity) | 8.3303 | 0.5966 | -0.0049 | 0.6810 | 0.614 |
| focal epilepsy | COVID-19 (infection) | 2.5098 | 0.8674 | 3.8931e-05 | 0.9933 | 0.9 |
|  | COVID-19 (hospitalization) | 2.1257 | 0.7127 | -0.0101 | 0.5291 | 0.724 |
|  | COVID-19 (severity) | 8.2139 | 0.2228 | -0.0044 | 0.8181 | 0.808 |
| generalized epilepsy | COVID-19 (infection) | 2.1757e-04 | 0.9999 | -9.6328e-05 | 0.9906 | 0.423 |
|  | COVID-19 (hospitalization) | 2.3668 | 0.3062 | -0.0181 | 0.4239 | 0.787 |
|  | COVID-19 (severity) | 6.3035 | 0.0428 | -0.0170 | 0.8028 | 0.787 |
| epilepsy and its subtypes database from the ILAE | | | | | | |
| epilepsy | COVID-19 (infection) | 1.9739 | 0.9613 | -0.0026 | 0.7132 | 0.959 |
|  | COVID-19 (hospitalization) | 7.6304 | 0.5718 | 0.0007 | 0.9638 | 0.587 |
|  | COVID-19 (severity) | 4.1920 | 0.6507 | -0.0081 | 0.6017 | 0.76 |
| focal epilepsy | COVID-19 (infection) | 0.1292 | 0.9374 | 0.0007 | 0.9525 | - |
|  | COVID-19 (hospitalization) | 4.0876 | 0.7696 | 0.0068 | 0.5911 | 0.767 |
|  | COVID-19 (severity) | 2.4661 | 0.9818 | -0.0034 | 0.7871 | 0.987 |
| generalized epilepsy | COVID-19 (infection) | 2.9002 | 0.8941 | -0.0045 | 0.6801 | 0.894 |
|  | COVID-19 (hospitalization) | 5.4415 | 0.7943 | -0.0016 | 0.9417 | 0.807 |
|  | COVID-19 (severity) | 0.6159 | 0.9989 | -0.0007 | 0.9859 | 0.998 |

**Table S5. Power calculation for MR analysis of epilepsy and its subtypes on Covid-19 (infection, hospitalization, severity).**

| **Exposure** | **Outcome** | **Sample size** | **Proportion of cases** | **OR** | **R^2^** | **Power** |
| --- | --- | --- | --- | --- | --- | --- |
| epilepsy and its subtypes database from FinnGen consortium | | | | | | |
| epilepsy | COVID-19 (infection) | 2942817 | 0.0543 | 0.9608 | 0.0182 | 54% |
|  | COVID-19 (hospitalization) | 2401372 | 0.0187 | 0.9171 | 0.0184 | 66% |
|  | COVID-19 (severity) | 1163698 | 0.0156 | 1.1202 | 0.0287 | 78% |
| focal epilepsy, mode | COVID-19 (infection) | 2942817 | 0.0543 | 1.0032 | 0.1489 | 8% |
|  | COVID-19 (hospitalization) | 2401372 | 0.0187 | 0.9614 | 0.0837 | 65% |
|  | COVID-19 (severity) | 1163698 | 0.0156 | 0.9611 | 0.1489 | 52% |
| generalized epilepsy, mode | COVID-19 (infection) | 2942817 | 0.0543 | 1.0124 | 0.0287 | 13% |
|  | COVID-19 (hospitalization) | 2401372 | 0.0187 | 1.0523 | 0.0287 | 46% |
|  | COVID-19 (severity) | 1163698 | 0.0156 | 1.1202 | 0.0287 | 78% |
| epilepsy and its subtypes database from the ILAE | | | | | | |
| epilepsy | COVID-19 (infection) | 2942817 | 0.0543 | 0.9890 | 0.2127 | 51% |
|  | COVID-19 (hospitalization) | 2401372 | 0.0187 | 0.9766 | 0.2634 | 71% |
|  | COVID-19 (severity) | 1163698 | 0.0156 | 0.9677 | 0.2699 | 61% |
| focal epilepsy | COVID-19 (infection) | 2942817 | 0.0543 | 1.0067 | 0.1217 | 12% |
|  | COVID-19 (hospitalization) | 2401372 | 0.0187 | 0.9812 | 0.2805 | 55% |
|  | COVID-19 (severity) | 1163698 | 0.0156 | 0.9766 | 0.4974 | 60% |
| generalized epilepsy | COVID-19 (infection) | 2942817 | 0.0543 | 1.0283 | 0.1197 | 97% |
|  | COVID-19 (hospitalization) | 2401372 | 0.0187 | 1.0279 | 0.1465 | 61% |
|  | COVID-19 (severity) | 1163698 | 0.0156 | 1.0283 | 0.0156 | 26% |

MR, Mendelian randomization; OR, odds ratio; Type-I error rate (α) was set as 0.05.

**Table S6. Characteristics of selected SNPs for COVID-19 (infection, hospitalization, severity).**

| **SNP** | **Trait** | **Chr** | **Pos.** | **Effect allele** | **Other allele** | **EAF** | **Beta** | **SE** | ***P value*** | **R^2^** | **F statistic** |
| --- | --- | --- | --- | --- | --- | --- | --- | --- | --- | --- | --- |
| rs10850097 | COVID-19 (infection) | 12 | 112923312 | T | C | 0.6789 | 0.028587 | 0.00436 | 5.50E-11 | 0.000356298 | 69.92583723 |
| rs17860169 | COVID-19 (infection) | 21 | 33240996 | G | A | 0.3374 | 0.039565 | 0.0043246 | 5.76E-20 | 0.000699921 | 137.4113352 |
| rs2260685 | COVID-19 (infection) | 3 | 195770872 | C | T | 0.4625 | 0.026488 | 0.0042375 | 4.08E-10 | 0.000348834 | 68.46044152 |
| rs35044562 | COVID-19 (infection) | 3 | 45867532 | G | A | 0.0773 | 0.13537 | 0.007549 | 6.56E-72 | 0.002613746 | 514.1260928 |
| rs7295014 | COVID-19 (infection) | 12 | 132491403 | A | G | 0.6386 | 0.025314 | 0.0043105 | 4.29E-09 | 0.00029578 | 58.04525455 |
| rs73005873 | COVID-19 (infection) | 19 | 8896954 | A | G | 0.3645 | 0.028961 | 0.0046884 | 6.53E-10 | 0.000388571 | 76.26207422 |
| rs75586969 | COVID-19 (infection) | 21 | 33893155 | T | C | 0.08208 | 0.042867 | 0.0072118 | 2.78E-09 | 0.000276897 | 54.33857753 |
| rs7949972 | COVID-19 (infection) | 11 | 34480495 | T | C | 0.3562 | -0.025863 | 0.0042089 | 8.00E-10 | 0.000306784 | 60.20541768 |
| rs1405655 | COVID-19 (infection) | 19 | 50379362 | C | T | 0.3322 | 0.026014 | 0.0042786 | 1.20E-09 | 0.000300255 | 58.92374819 |
| rs1123573 | COVID-19 (infection) | 2 | 60480453 | G | A | 0.3668 | -0.025387 | 0.0043319 | 4.62E-09 | 0.00029938 | 58.75200192 |
| rs1405655 | COVID-19 (infection) | 19 | 50379362 | C | T | 0.3322 | 0.026014 | 0.0042786 | 1.20E-09 | 0.000300255 | 58.92374819 |
| rs10774679 | COVID-19 (hospitalization) | 12 | 112936943 | T | C | 0.6527 | 0.071847 | 0.0088009 | 3.25E-16 | 0.002340268 | 216.6528654 |
| rs10890422 | COVID-19 (hospitalization) | 1 | 46810098 | C | T | 0.4054 | -0.047171 | 0.0086191 | 4.43E-08 | 0.001072726 | 99.18275138 |
| rs11208552 | COVID-19 (hospitalization) | 1 | 64947147 | T | G | 0.6393 | -0.054975 | 0.0091889 | 2.19E-09 | 0.001393835 | 128.9134665 |
| rs12329760 | COVID-19 (hospitalization) | 21 | 41480570 | T | C | 0.2616 | -0.055389 | 0.0098937 | 2.16E-08 | 0.00118524 | 109.5980261 |
| rs12585036 | COVID-19 (hospitalization) | 13 | 112881427 | T | C | 0.2076 | 0.097378 | 0.010296 | 3.15E-21 | 0.003119777 | 289.042526 |
| rs149533170 | COVID-19 (hospitalization) | 9 | 21172826 | A | G | 0.0076 | 0.28165 | 0.049626 | 1.38E-08 | 0.001221441 | 112.9495299 |
| rs17412601 | COVID-19 (hospitalization) | 3 | 101780431 | C | T | 0.3408 | -0.066775 | 0.0090438 | 1.54E-13 | 0.002003432 | 185.4072607 |
| rs17763742 | COVID-19 (hospitalization) | 3 | 45805277 | G | A | 0.07814 | 0.48852 | 0.015694 | 1.00E-200 | 0.034382153 | 3288.584443 |
| rs2897075 | COVID-19 (hospitalization) | 7 | 100032719 | T | C | 0.3648 | 0.049692 | 0.0086419 | 8.92E-09 | 0.001144375 | 105.8148838 |
| rs5023077 | COVID-19 (hospitalization) | 12 | 132565387 | C | T | 0.5167 | -0.066746 | 0.0084514 | 2.84E-15 | 0.002225029 | 205.960694 |
| rs76608815 | COVID-19 (hospitalization) | 21 | 33980963 | T | C | 0.0898 | 0.13283 | 0.014161 | 6.60E-21 | 0.002883109 | 267.0522489 |
| rs7671107 | COVID-19 (hospitalization) | 4 | 25447603 | A | G | 0.7314 | -0.071892 | 0.010204 | 1.84E-12 | 0.00203073 | 187.9386635 |
| rs7897438 | COVID-19 (hospitalization) | 10 | 112972548 | A | C | 0.2409 | -0.053738 | 0.009847 | 4.83E-08 | 0.001056158 | 97.64925233 |
| rs7949972 | COVID-19 (hospitalization) | 11 | 34480495 | T | C | 0.3545 | -0.087013 | 0.008542 | 2.28E-24 | 0.00346506 | 321.1437303 |
| rs79611697 | COVID-19 (hospitalization) | 9 | 15795835 | T | G | 0.0692 | 0.10347 | 0.018776 | 3.57E-08 | 0.001379181 | 127.5563133 |
| rs10066378 | COVID-19 (hospitalization) | 5 | 132441275 | C | T | 0.1324 | 0.074001 | 0.011575 | 1.63E-10 | 0.001258093 | 116.3431021 |
| rs1123573 | COVID-19 (hospitalization) | 2 | 60480453 | G | A | 0.3645 | -0.071705 | 0.0093078 | 1.32E-14 | 0.002382001 | 220.5255424 |
| rs117169628 | COVID-19 (hospitalization) | 16 | 89196249 | A | G | 0.133 | 0.098534 | 0.012798 | 1.37E-14 | 0.002239097 | 207.2658206 |
| rs12660421 | COVID-19 (hospitalization) | 6 | 41520640 | A | G | 0.0424 | 0.24033 | 0.02066 | 2.82E-31 | 0.004684963 | 434.7372386 |
| rs1405655 | COVID-19 (hospitalization) | 19 | 50379362 | C | T | 0.3326 | 0.077686 | 0.0087352 | 5.92E-19 | 0.002679316 | 248.1249 |
| rs3848456 | COVID-19 (hospitalization) | 17 | 49863260 | A | C | 0.0388 | 0.19199 | 0.020254 | 2.56E-21 | 0.002745967 | 254.3142423 |
| rs9636867 | COVID-19 (hospitalization) | 21 | 33237639 | G | A | 0.3378 | 0.13121 | 0.0087727 | 1.41E-50 | 0.007702163 | 716.8888982 |
| rs11131812 | COVID-19 (severity) | 4 | 167765627 | A | G | 0.6043 | 0.071172 | 0.013042 | 4.84E-08 | 0.002422518 | 128.4485974 |
| rs116415481 | COVID-19 (severity) | 1 | 155317031 | T | C | 0.0301 | 0.21867 | 0.038311 | 1.14E-08 | 0.002789217 | 147.9463596 |
| rs12046291 | COVID-19 (severity) | 1 | 64948270 | G | A | 0.6648 | -0.087293 | 0.013994 | 4.43E-10 | 0.003396126 | 180.2479389 |
| rs12627323 | COVID-19 (severity) | 21 | 33960832 | A | G | 0.0889 | 0.18217 | 0.021522 | 2.57E-17 | 0.005377541 | 285.9792434 |
| rs2897075 | COVID-19 (severity) | 7 | 100032719 | T | C | 0.3745 | 0.081288 | 0.012981 | 3.80E-10 | 0.003095722 | 164.2546119 |
| rs9305744 | COVID-19 (severity) | 21 | 41471061 | A | G | 0.2698 | -0.086349 | 0.015139 | 1.17E-08 | 0.002937842 | 155.8530064 |
| rs10066378 | COVID-19 (severity) | 5 | 132441275 | C | T | 0.1039 | 0.11467 | 0.018159 | 2.71E-10 | 0.002741051 | 145.3845555 |
| rs10850097 | COVID-19 (severity) | 12 | 112923312 | T | C | 0.7288 | 0.092001 | 0.013585 | 1.27E-11 | 0.003727199 | 197.8851816 |
| rs1123573 | COVID-19 (severity) | 2 | 60480453 | G | A | 0.3336 | -0.1157 | 0.013925 | 9.67E-17 | 0.006182641 | 329.0610555 |
| rs11614702 | COVID-19 (severity) | 12 | 132481571 | A | G | 0.49959 | 0.096146 | 0.012696 | 3.65E-14 | 0.004621488 | 245.5854025 |
| rs117169628 | COVID-19 (severity) | 16 | 89196249 | A | G | 0.1220 | 0.15 | 0.019052 | 3.46E-15 | 0.005175776 | 275.1934717 |
| rs11879501 | COVID-19 (severity) | 19 | 50368861 | G | A | 0.3333 | 0.081475 | 0.013436 | 1.33E-09 | 0.002960699 | 157.0692152 |
| rs12534422 | COVID-19 (severity) | 7 | 75634474 | T | C | 0.2407 | 0.078072 | 0.013712 | 1.24E-08 | 0.002563893 | 135.9639568 |
| rs12585036 | COVID-19 (severity) | 13 | 112881427 | T | C | 0.2125 | 0.1383 | 0.01564 | 9.32E-19 | 0.006401531 | 340.7861706 |
| rs12614007 | COVID-19 (severity) | 2 | 57089368 | A | G | 0.7551 | 0.088561 | 0.01555 | 1.23E-08 | 0.002900736 | 153.8788396 |
| rs17763742 | COVID-19 (severity) | 3 | 45805277 | G | A | 0.0822 | 0.71404 | 0.023095 | 1.00E-200 | 0.076963942 | 4410.396775 |
| rs2496644 | COVID-19 (severity) | 6 | 41515007 | C | A | 0.9548 | -0.33552 | 0.032946 | 2.33E-24 | 0.009716675 | 518.9998402 |
| rs3848456 | COVID-19 (severity) | 17 | 49863260 | A | C | 0.0381 | 0.2911 | 0.032584 | 4.11E-19 | 0.006211111 | 330.585799 |
| rs7664615 | COVID-19 (severity) | 4 | 25446871 | G | A | 0.8105 | -0.092768 | 0.016524 | 1.97E-08 | 0.002643557 | 140.1997509 |
| rs7949972 | COVID-19 (severity) | 11 | 34480495 | T | C | 0.3526 | -0.11794 | 0.013037 | 1.47E-19 | 0.00635049 | 338.0516393 |
| rs9636867 | COVID-19 (severity) | 21 | 33237639 | G | A | 0.3371 | 0.18763 | 0.013499 | 6.40E-44 | 0.015734079 | 845.5472847 |

Chr, chromosome; EAF, Effect allele frequency; Pos, position; SE, standard error; SNP, single nucleotide polymorphism.

| **Table S7. Characteristics of selected SNPs for epilepsy and its substypes**.**SNP** | **Trait** | **Chr** | **Pos.** | **Effect allele** | **Other allele** | **EAF** | **Beta** | **SE** | ***P value*** | **R^2^** | **F statistic** |
| --- | --- | --- | --- | --- | --- | --- | --- | --- | --- | --- | --- |
| epilepsy and its subtypes database from the FinnGen consortium | | | | | | | | | | | |
| rs117288293 | epilepsy | 7 | 11886938 | C | T | 0.0397917 | -0.206575 | 0.038846 | 1.05E-07 | 0.003260945 | 69.20771308 |
| rs12635851 | epilepsy | 3 | 45072810 | C | T | 0.282168 | -0.0790221 | 0.0159715 | 7.51E-07 | 0.002529634 | 53.64758639 |
| rs2538068 | epilepsy | 7 | 54908669 | A | G | 0.243199 | 0.0843932 | 0.0164021 | 2.67E-07 | 0.002621733 | 55.60592377 |
| rs55989371 | epilepsy | 13 | 98625727 | C | T | 0.245853 | 0.0766829 | 0.0164428 | 3.11E-06 | 0.002180512 | 46.22735014 |
| rs62371306 | epilepsy | 5 | 111459344 | T | C | 0.0099716 | -0.377533 | 0.0810661 | 3.21E-06 | 0.002814183 | 59.69923175 |
| rs6745936 | epilepsy | 2 | 16029651 | A | G | 0.712589 | 0.0795831 | 0.0159709 | 6.26E-07 | 0.002594264 | 55.02180174 |
| rs7942330 | epilepsy | 11 | 6979289 | C | T | 0.307639 | 0.0744096 | 0.0154245 | 1.41E-06 | 0.002358641 | 50.01265361 |
| rs11598336 | epilepsy | 10 | 107176484 | T | C | 0.472914 | 0.0723355 | 0.014315 | 4.35E-07 | 0.002608535 | 55.32526729 |
| rs13148012 | epilepsy | 4 | 86567165 | T | C | 0.0342863 | -0.204841 | 0.0419618 | 1.05E-06 | 0.002778643 | 58.94319612 |
| rs149672320 | epilepsy | 4 | 121399382 | A | G | 0.0218808 | 0.211837 | 0.0458701 | 3.87E-06 | 0.001920829 | 40.71141634 |
| rs185076690 | epilepsy | 2 | 142733050 | T | C | 0.00670689 | -0.485226 | 0.102639 | 2.27E-06 | 0.003137016 | 66.56926531 |
| rs11598336 | epilepsy | 10 | 107176484 | T | C | 0.472914 | 0.0723355 | 0.014315 | 4.35E-07 | 0.002608535 | 55.32526729 |
| rs149012739 | focal epilepsy | 8 | 102997459 | T | G | 0.00333927 | 1.1151 | 0.243275 | 4.57E-06 | 0.008276687 | 234.7196955 |
| rs373623042 | focal epilepsy | 10 | 12863057 | T | C | 0.00996503 | 0.76301 | 0.165233 | 3.88E-06 | 0.011487343 | 326.8292207 |
| rs4789331 | focal epilepsy | 17 | 76623045 | A | G | 0.263761 | 0.212852 | 0.0459283 | 3.58E-06 | 0.017596037 | 503.7421422 |
| rs58819425 | focal epilepsy | 17 | 28267614 | G | T | 0.0831386 | -0.409659 | 0.0847905 | 1.36E-06 | 0.025584763 | 738.4495936 |
| rs7624539 | focal epilepsy | 3 | 30252315 | T | C | 0.218406 | -0.246601 | 0.053366 | 3.82E-06 | 0.020761821 | 596.2942599 |
| rs205994 | focal epilepsy | 6 | 164029914 | A | G | 0.808697 | 0.284943 | 0.0564152 | 4.40E-07 | 0.025121964 | 724.7476687 |
| rs77185258 | focal epilepsy | 11 | 91931335 | T | C | 0.0125493 | -1.27154 | 0.273884 | 3.44E-06 | 0.040070519 | 1174.002878 |
| rs205994 | focal epilepsy | 6 | 164029914 | A | G | 0.808697 | 0.284943 | 0.0564152 | 4.40E-07 | 0.025121964 | 724.7476687 |
| rs11715870 | generalized epilepsy | 3 | 177467113 | A | G | 0.245767 | 0.168584 | 0.0360357 | 2.89E-06 | 0.010536389 | 1186.000533 |
| rs147155164 | generalized epilepsy | 3 | 20289118 | A | G | 0.00465202 | 0.829274 | 0.178243 | 3.28E-06 | 0.00636858 | 713.8553316 |
| rs6482647 | generalized epilepsy | 10 | 128028109 | G | A | 0.570375 | -0.15524 | 0.0318407 | 1.09E-06 | 0.011811017 | 1331.190476 |
| epilepsy and its subtypes database from the ILAE | | | | | | | | | | | |
| rs10873348 | epilepsy | 14 | 83243744 | T | G | 0.7095 | 0.305178 | 0.057 | 8.62E-08 | 0.038391491 | 43.67030468 |
| rs13090746 | epilepsy | 3 | 103064886 | A | G | 0.3131 | 0.2423878 | 0.0529 | 4.62E-06 | 0.02527133 | 28.35919502 |
| rs1356794 | epilepsy | 3 | 20425138 | T | C | 0.3866 | -0.196847 | 0.0427 | 4.02E-06 | 0.018377787 | 20.47851105 |
| rs1831150 | epilepsy | 1 | 207497093 | A | G | 0.71 | 0.2487852 | 0.0522 | 1.88E-06 | 0.02548798 | 28.60867592 |
| rs4491514 | epilepsy | 16 | 80492573 | A | G | 0.1989 | -0.4317012 | 0.0926 | 3.14E-06 | 0.059390642 | 69.06503963 |
| rs4817045 | epilepsy | 21 | 25637806 | T | G | 0.8601 | -0.238666 | 0.047 | 3.82E-07 | 0.013708116 | 15.20273943 |
| rs6839308 | epilepsy | 4 | 175107519 | A | C | 0.3758 | 0.2647004 | 0.0551 | 1.56E-06 | 0.032871516 | 37.17792097 |
| rs7952149 | epilepsy | 11 | 81548231 | T | C | 0.7745 | -0.2135124 | 0.0467 | 4.84E-06 | 0.015923707 | 17.69966102 |
| rs940580 | epilepsy | 4 | 57429975 | T | G | 0.2576 | -0.2139908 | 0.0446 | 1.60E-06 | 0.017514753 | 19.49968159 |
| rs9901765 | epilepsy | 17 | 58189445 | A | C | 0.9021 | 0.305696 | 0.0656 | 3.17E-06 | 0.016506192 | 18.35797623 |
| rs6938732 | epilepsy | 6 | 127423687 | A | G | 0.4318 | 0.53548 | 0.11 | 1.13E-06 | 0.140702033 | 179.1043479 |
| rs10873348 | focal epilepsy | 14 | 83243744 | T | G | 0.7085 | 0.239859 | 0.0522 | 4.32E-06 | 0.023764049 | 38.28787226 |
| rs13090746 | focal epilepsy | 3 | 103064886 | A | G | 0.3111 | 0.2232321 | 0.0481 | 3.47E-06 | 0.021359913 | 34.32986284 |
| rs1658164 | focal epilepsy | 18 | 4595501 | A | G | 0.5351 | 0.2639523 | 0.0543 | 1.17E-06 | 0.034663738 | 56.47969767 |
| rs2753616 | focal epilepsy | 14 | 85607548 | A | G | 0.5893 | -0.1370275 | 0.0295 | 3.40E-06 | 0.009088801 | 14.42671486 |
| rs4591547 | focal epilepsy | 4 | 159436725 | A | G | 0.6008 | -0.3994406 | 0.0871 | 4.52E-06 | 0.076534079 | 130.3555653 |
| rs6541281 | focal epilepsy | 1 | 231677388 | T | C | 0.4477 | 0.354996 | 0.076 | 2.99E-06 | 0.062321666 | 104.539583 |
| rs6839308 | focal epilepsy | 4 | 175107519 | A | C | 0.3718 | 0.3000636 | 0.0612 | 9.46E-07 | 0.042059484 | 69.0591118 |
| rs75374212 | focal epilepsy | 11 | 6680283 | T | C | 0.0675 | 0.292005 | 0.063 | 3.56E-06 | 0.010734039 | 17.06654826 |
| rs6938732 | focal epilepsy | 6 | 127423687 | A | G | 0.4377 | 0.5853232 | 0.1232 | 2.03E-06 | 0.168642139 | 319.0609725 |
| rs7083521 | focal epilepsy | 10 | 22817262 | T | G | 0.0848 | 0.5575812 | 0.1166 | 1.74E-06 | 0.048256754 | 79.75058772 |
| rs11639540 | generalized epilepsy | 16 | 7317140 | A | C | 0.5135 | -0.183645 | 0.0385 | 1.84E-06 | 0.01685045 | 20.45508778 |
| rs13200150 | generalized epilepsy | 6 | 127988623 | A | G | 0.6924 | 0.2048288 | 0.0352 | 5.92E-09 | 0.01787127 | 21.71682989 |
| rs133565 | generalized epilepsy | 22 | 48244181 | A | G | 0.3201 | -0.1817352 | 0.0344 | 1.27E-07 | 0.014376025 | 17.40752346 |
| rs2612767 | generalized epilepsy | 17 | 79186622 | T | C | 0.3372 | -0.1495325 | 0.0325 | 4.21E-06 | 0.009994734 | 12.04878249 |
| rs285317 | generalized epilepsy | 3 | 36210223 | T | C | 0.7842 | 0.2523755 | 0.0533 | 2.20E-06 | 0.021557712 | 26.29522432 |
| rs4817354 | generalized epilepsy | 21 | 30806600 | A | G | 0.6246 | 0.202284 | 0.036 | 1.93E-08 | 0.019188866 | 23.3492724 |
| rs4981631 | generalized epilepsy | 14 | 26843246 | A | C | 0.7394 | 0.162519 | 0.0355 | 4.70E-06 | 0.010178696 | 12.27283162 |
| rs7350429 | generalized epilepsy | 10 | 102006033 | A | G | 0.7107 | -0.1679652 | 0.0333 | 4.57E-07 | 0.011601212 | 14.00814369 |
| rs8041905 | generalized epilepsy | 15 | 61744401 | T | C | 0.5976 | -0.1836408 | 0.0357 | 2.69E-07 | 0.016219479 | 19.67651176 |
| rs9297025 | generalized epilepsy | 6 | 16974472 | A | G | 0.7857 | 0.160716 | 0.0295 | 5.09E-08 | 0.008698155 | 10.47202468 |

Chr, chromosome; EAF, Effect allele frequency; Pos, position; SE, standard error; SNP, single nucleotide polymorphism.
